# Supplementary material for: Mirror-image ligand discovery enabled by single-shot fast-flow synthesis of D-proteins
Source: Nat Commun. 2024 Feb 28;15:1813. doi: 10.1038/s41467-024-45634-z (PMC10901774; doi:10.1038/s41467-024-45634-z)
Supplement: Supplementary file 1 — Supplementary Information [file 41467_2024_45634_MOESM1_ESM.pdf]

Supplementary Information for

Mirror-Image Ligand Discovery Enabled by Single-Shot Fast-Flow Synthesis of D-Proteins

Alex J. Callahan[1#], Satish Gandhesiri[1#], Tara L. Travaline[2], Rahi M. Reja[1], Lia Lozano Salazar[1], Stephanie Hanna[1], Yen-Chun Lee[1,a], Kunhua Li[2], Olena S. Tokareva[2], Jean-Marie Swiecicki[2,b], Andrei Loas[1], Gregory L. Verdine[2,3,4,5], John H. McGee[2*], Bradley L. Pentelute[1,6,7,8*]

[1]Department of Chemistry, Massachusetts Institute of Technology, 77 Massachusetts Avenue, Cambridge, MA 02139, USA

[2]FOG Pharmaceuticals Inc., 30 Acorn Park Drive, Cambridge, MA 02140, USA

[3]Department of Stem Cell and Regenerative Biology, Harvard University, 7 Divinity Avenue, Cambridge, MA 02138, USA

[4]Department of Chemistry and Chemical Biology, Harvard University, 12 Oxford Street, Cambridge, MA 02138, USA

[5]Department of Molecular and Cellular Biology, Harvard University, 52 Oxford Street, Cambridge, MA 02138, USA

[6]The Koch Institute for Integrative Cancer Research, Massachusetts Institute of Technology, 500 Main Street, Cambridge, MA 02142, USA

[7]Center for Environmental Health Sciences, Massachusetts Institute of Technology, 77 Massachusetts Avenue, Cambridge, MA 02139, USA

[8]Broad Institute of MIT and Harvard, 415 Main Street, Cambridge, MA 02142, USA

[a]*Current address*: Department of Chemistry, National Cheng Kung University, No.1, University Road, Tainan City 701, Taiwan

[b]*Current address*: Relay Therapeutics, Inc., 399 Binney Street, 2nd Floor, Cambridge, MA 02139, USA

*Email: jmcgee@fogpharma.com, blp@mit.edu

[#]These authors contributed equally

This PDF file contains:

Supplementary Methods

Supplementary Results

Supplementary Notes

Supplementary Tables 1-6

Supplementary Figures 1-15

Supplementary References

Table of Contents

TABLE OF CONTENTS **2**

1. SUPPLEMENTARY METHODS **6**

1.1. CLEAVAGE PROTOCOLS 6
1.1.1. Protocol 1 – Reagent K *6*
1.1.2. Protocol 2 – Standard Cleavage Mix *6*
1.2. ANALYTICAL REVERSE PHASE HIGH PRESSURE LIQUID CHROMATOGRAPHY METHODS 7
1.2.1. Method 1 – 5% to 65% in 30 min on Agilent 1200 HPLC *7*
1.2.2. Method 2 – 5% to 65% in 15 min on Agilent 1200 HPLC *7*
1.2.3. Method 3 – 5% to 65% in 30 min on Agilent 1290 UHPLC *7*
1.3. REVERSE PHASE HIGH PRESSURE LIQUID CHROMATOGRAPHY MASS SPECTROMETRY METHODS 7
1.3.1. Method 1 – 6545 1-91 in 7 min *7*
1.3.2. Method 2 – 6545 1-91 in 15 min *7*
1.3.3. Method 3 – 6550-1 5-95 in 10 min *8*
1.3.4. Method 4 – 6550-2 1-91 in 15 min *8*
1.4. PREPARATIVE REVERSE PHASE PURIFICATION METHODS 8
1.4.1. Method 1 – 2-Stage Semiprep *8*
1.4.2. Method 2 – 2-Stage Prep *9*
1.4.3. Method 3 – 1-Stage Semiprep *9*
1.5. AUTOMATED FLOW PEPTIDE SYNTHESIS CONDITIONS 10
1.6. DETERMINATION OF PROTEIN CONCENTRATION 10
1.6.1. A_{280} Measurement *10*
1.6.2. Bradford Protein Assay *10*
1.7. BCL11A BINDING TO DNA VIA TR-FRET 11
1.8. CHIP BINDING TO HSP PEPTIDE VIA TR-FRET 11
1.9. MDM2 BINDING TO P53 PEPTIDE VIA TR-FRET 12
1.10. MAX-MAX AND MYC-MAX BINDING TO DNA VIA TR-FRET 12
1.11. BARNASE CATALYSIS OF RNA SUBSTRATE VIA FRET 13
1.12. ERG AND IRAK2 CIRCULAR DICHROISM 15
1.13. NEMO BINDING TO IKKB PEPTIDE WITH SPR 16
1.14. FKBP12 BINDING TO RAPAMYCIN/MTOR WITH SPR 17
1.15. YAP1 BINDING TO DENDRIN WITH SPR 18
1.16. NEMO_IZIP BINDING TO IKKB WITH SPR 19
1.17. MDM2 PROTEIN EXPRESSION 19
1.18. ERG PROTEIN EXPRESSION 20
1.19. IRAK2 PROTEIN EXPRESSION 20
1.20. CHIP PROTEIN EXPRESSION 21
1.21. NEMO PROTEIN EXPRESSION 21
1.22. FKBP12 PROTEIN EXPRESSION 22
1.23. BCL11A PROTEIN EXPRESSION 22
1.24. YAP1-WW1-WW2 PROTEIN EXPRESSION 23
1.25. NEMO_IZIP PROTEIN EXPRESSION 23
1.26. MDM2 PEPTIDE BINDERS FROM PHAGE DISPLAY 23
1.26.1. MDM2 Binder Sequences *23*
1.26.2. MDM2 Binder SPR Validation *23*
1.27. CHIP PEPTIDE BINDERS FROM PHAGE DISPLAY 27
1.27.1. CHIP Binder Sequences *27*

1.27.2. CHIP Binder FP Validation.....27
1.28. List of Protein Sequences.....29

2. SUPPLEMENTARY RESULTS.....30

2.1. Crude HPLC Data for L-Protein Targets.....30
2.2. Crude HPLC Data for D-Protein Targets.....31
2.3. MDM2 from AFPS.....32
2.3.1. L-MDM2 AFPS Results.....32
2.3.2. D-MDM2 AFPS Results.....33
2.4. ERG from AFPS.....34
2.4.1. L-ERG AFPS Results.....34
2.4.2. D-ERG AFPS Results.....35
2.5. Barnase from AFPS.....36
2.5.1. L-Barnase AFPS Results.....36
2.5.2. D-Barnase AFPS Results.....37
2.6. IRAK2 from AFPS.....38
2.6.1. L-IRAK2 AFPS Results.....38
2.6.2. D-IRAK2 AFPS Results.....39
2.7. CHIP from AFPS.....40
2.7.1. L-CHIP AFPS Results.....40
2.7.2. D-CHIP AFPS Results.....41
2.8. NEMO from AFPS.....42
2.8.1. L-NEMO AFPS Results.....42
2.8.2. D-NEMO AFPS Results.....43
2.9. FKBP12 from AFPS.....44
2.9.1. L-FKBP12 AFPS Results.....44
2.9.2. D-FKBP12 AFPS Results.....45
2.10. BCL11a from AFPS.....46
2.10.1. L-BCL11a AFPS Results.....46
2.10.2. D-BCL11a AFPS Results.....47
2.11. YAP1 from AFPS.....48
2.11.1. L-YAP1 AFPS Results.....48
2.11.2. D-YAP1 AFPS Results.....49
2.12. NEMO_iZIP from AFPS.....50
2.12.1. L-NEMO_iZIP AFPS Results.....50
2.12.2. D-NEMO_iZIP AFPS Results.....51
2.13. Max from AFPS.....52
2.13.1. L-Max AFPS Results.....52
2.13.2. D-Max AFPS Results.....53
2.14. Max-nb from AFPS.....54
2.14.1. L-Max-nb AFPS Results.....54
2.14.2. D-Max-nb AFPS Results.....55
2.15. Max Oxidative Addition Complex Synthesis.....56
2.15.1. L-Max Oxidative Addition Complex Results.....57
2.15.2. D-Max Oxidative Addition Complex Results.....58
2.16. Max-Max Conjugation.....59
2.16.1. L-Max-Max-nb Dimerization Results.....60
2.16.2. D-Max-Max-nb Dimerization Results.....61
2.17. Myc from AFPS.....62

2.17.1. L-Myc AFPS Results ... 62
2.17.2. D-Myc AFPS Results ... 63
2.18. Myc Oxidative Addition Complex Synthesis ... 64
2.18.1. L-Myc Oxidative Addition Complex Results ... 65
2.18.2. D-Myc Oxidative Addition Complex Results ... 66
2.19. Myc-Max Conjugation ... 67
2.19.1. L-Myc-Max-nb Dimerization Results ... 68
2.19.2. D-Myc-Max-nb Dimerization Results ... 69
2.20. MDM2 Folding Results ... 70
2.20.1. Dilution Folding of L-MDM2 ... 70
2.20.2. Dilution Folding of D-MDM2 ... 70
2.21. ERG Folding Results ... 71
2.21.1. SEC Folding of L-ERG ... 71
2.21.2. SEC Folding of D-ERG ... 71
2.22. Barnase Folding Results ... 72
2.22.1. SEC Folding of L-Barnase ... 72
2.22.2. SEC Folding of D-Barnase ... 72
2.23. IRAK2 Folding Results ... 73
2.23.1. SEC Folding of L-IRAK2 ... 73
2.23.2. SEC Folding of D-IRAK2 ... 74
2.24. CHIP Folding Results ... 75
2.24.1. SEC Folding of L-CHIP ... 75
2.24.2. SEC Folding of D-CHIP ... 75
2.25. NEMO Folding Results ... 76
2.25.1. SEC Folding of L-NEMO ... 76
2.25.2. SEC Folding of D-NEMO ... 76
2.26. FKBP12 Folding Results ... 77
2.26.1. SEC Folding of L-FKBP12* ... 77
2.26.2. SEC Folding of D-FKBP12 ... 78
2.27. BCL11a Folding Results ... 79
2.27.1. SEC Folding of L-BCL11a ... 79
2.27.2. SEC Folding of D-BCL11a ... 80
2.28. YAP1 Folding Results ... 81
2.28.1. SEC Folding of L-YAP1 ... 81
2.28.2. SEC Folding of D-YAP1 ... 81
2.29. NEMO_iZIP Folding Results ... 82
2.29.1. SEC Folding of L-NEMO_iZIP ... 82
2.29.2. SEC Folding of D-NEMO_iZIP ... 83
2.30. Max-Max Folding Results ... 84
2.30.1. SEC Folding of L-Max-Max ... 84
2.30.2. SEC Folding of D-Max-Max ... 84
2.31. Myc-Max Folding Results ... 85
2.31.1. SEC Folding of L-Myc-Max ... 85
2.31.2. SEC Folding of D-Myc-Max ... 85

3. SUPPLEMENTARY NOTES ... 86

3.1. Crystal Structure Collection Methods ... 86
3.2. MDM2 Cocrystal Structures ... 87
3.2.1. MDM2-H101 Data Collection and Refinement Statistics ... 87

3.2.2. MDM2-D-H102 Data Collection and Refinement Statistics *89*
3.2.3. MDM2-D-H103 Data Collection and Refinement Statistics *91*
3.2.4. MDM2-D-H103-Alt Data Collection and Refinement Statistics *93*
3.3. CHIP CO-CRYSTAL STRUCTURES 95
3.3.1. CHIP-D-H201 Data Collection and Refinement Statistics *95*
3.3.2. CHIP-D-H202 Data Collection and Refinement Statistics *97*
3.3.3. CHIP-D-H203 Data Collection and Refinement Statistics *99*
3.3.4. CHIP-D-H204 Data Collection and Refinement Statistics *101*

4. SUPPLEMENTARY REFERENCES 103

1. Supplementary Methods

1.1. Cleavage Protocols

1.1.1. Protocol 1 – Reagent K

A fresh solution of a cleavage mixture was prepared according to the following recipe and used immediately: trifluoroacetic acid (82.5% v/v), water (5% v/v), phenol (5% v/v, melted in 60 °C water bath prior to use), thioanisole (5% v/v), and 1,2-ethanedithiol (2.5% v/v). A ratio of 1 mL of cleavage mixture per 2.5 μmol of protected peptide was used. The cleavage mixture was drawn-up with the reaction syringe containing the peptidyl resin, and the assembly capped with a Luer-tip syringe tip cap. The assembly was placed on a nutating mixer and allowed to stand for 3.5 h. During this time, an aliquot of 50 mL of diethyl ether was prepared in a 50 mL Falcon tube and chilled on dry ice for at least 1 h. At the end of the incubation time, the cleavage supernatant in the reaction syringe was expelled into a fresh 50 mL Falcon tube, and the resin was retained by the internal frit of the reaction syringe. An additional aliquot of 2.5 mL of TFA was drawn up into the reaction syringe, the assembly gently mixed, and the aliquot expelled into the same Falcon tube containing the cleavage supernatant. The pre-chilled diethyl ether was added to the cleavage supernatant, and a strong white precipitate was generated. A fresh screwcap was used to seal the Falcon tube containing the cleavage mixture, and then the assembly was centrifuged at 4 °C for 4 min at 3220 × g (4000 rpm) while maintaining the temperature at 4 °C. The supernatant was decanted into the waste, and the solid pellet was retained for processing.

1.1.2. Protocol 2 – Standard Cleavage Mix

A fresh solution of a cleavage mixture was prepared according to the following recipe and used immediately: trifluoroacetic acid (94% v/v), water (2.5% v/v), 1,2-ethanedithiol (2.5% v/v), and triisopropylsilane (1% v/v). A ratio of 1 mL of cleavage mixture per 2.5 μmol of protected peptide was used. The cleavage mixture was drawn-up with the reaction syringe containing the peptidyl resin, and the assembly capped with a Luer-tip syringe tip cap. The assembly was placed on a nutating mixer and let stand for 2 h. During this time, an aliquot of 50 mL of diethyl ether was prepared in a 50 mL Falcon tube and chilled on dry ice for at least 1 h. At the end of the incubation time, the cleavage supernatant in the reaction syringe was expelled into a fresh 50 mL Falcon tube, and the resin was retained by the internal frit of the reaction syringe. An additional aliquot of 2.5 mL of trifluoroacetic acid was drawn up into the reaction syringe, the assembly gently mixed, and the aliquot expelled into the same Falcon tube containing the cleavage supernatant. The pre-chilled diethyl ether was added to the cleavage supernatant, and a strong white precipitate was generated. A fresh screwcap was used to seal the Falcon tube containing the cleavage mixture, and then the assembly was centrifuged at 4 °C for 4 min at 3220 × g (4000 rpm) while maintaining the temperature at 4 °C. The supernatant was decanted into the waste, and the solid pellet retained for processing.

1.2. Analytical Reverse Phase High Pressure Liquid Chromatography Methods

1.2.1. Method 1 – 5% to 65% in 30 min on Agilent 1200 HPLC

Analytical HPLC was carried out on an Agilent 1200 series system with UV detection at 214 nm. Column: Phenomenex Kinetex C18, (150 x 2.1 mm, 2.6 μm, 100 Å silica); flow rate 0.375 mL/minute; Solvent System: A = water with 0.1% TFA (v/v), B = acetonitrile with 0.08% TFA (v/v); Gradient: 3 min hold 5% B, 5-65% B gradient over 30 min, 3 min hold 65% B, 10 min post run 5% B.

1.2.2. Method 2 – 5% to 65% in 15 min on Agilent 1200 HPLC

Analytical HPLC was carried out on an Agilent 1200 series system with UV detection at 214 nm. Column: Phenomenex Kinetex C18, (150 x 2.1 mm, 2.6 μm, 100 Å silica); flow rate 0.375 mL/minute; Solvent System: A = water with 0.1% TFA (v/v), B = acetonitrile with 0.08% TFA (v/v); Gradient: 3 min hold 5% B, 5-65% B gradient over 15 min, 3 min hold 65% B, 10 min post run 5% B.

1.2.3. Method 3 – 5% to 65% in 30 min on Agilent 1290 UHPLC

Analytical HPLC was carried out on an Agilent 1290 series system with UV detection at 214 nm. Column: Phenomenex Kinetex C18, (150 x 2.1 mm, 2.6 μm, 100 Å silica); flow rate 0.375 mL/minute; Solvent System: A = water with 0.1% TFA (v/v), B = acetonitrile with 0.08% TFA (v/v); Gradient: 3 min hold 5% B, 5-65% B gradient over 30 min, 3 min hold 65% B, 10 min post run 5% B.

1.3. Reverse Phase High Pressure Liquid Chromatography Mass Spectrometry Methods

1.3.1. Method 1 – 6545 1-91 in 7 min

Analysis was performed on an Agilent 1290 Infinity HPLC coupled to an Agilent 6545 ESI-Q-TOF mass spectrometer. MS was run in positive ionization mode, extended dynamic range (2 GHz), and standard mass range (m/z in range 300 to 3000). The solvent mixtures used for LC-MS chromatography were: A = water + 0.1% formic acid (LC-MS-grade), B = acetonitrile + 0.1% formic acid (LC-MS-grade). The following condition was used for analysis. Column: Zorbax 300-SB C3 (5 μm, 150 x 2.1 mm, 300Å silica); Flow Rate: 0.6 mL/min; Gradient: linearly ramp from 1% B to 91% B 0 to 5 min, 91% B to 95% B 5 to 7 min. Post time is 1% B for 3 min. Flow rate is 0.8 mL/min. MS data was acquired from 1 to 5 min.

1.3.2. Method 2 – 6545 1-91 in 15 min

Analysis was performed on an Agilent 1290 Infinity HPLC coupled to an Agilent 6545 ESI-Q-TOF mass spectrometer. MS was run in positive ionization mode, extended dynamic range (2 GHz), and standard mass range (m/z in range 300 to 3000). The solvent mixtures used for LC-MS chromatography were: A = water + 0.1% formic acid (LC-MS-grade), B = acetonitrile + 0.1% formic acid (LC-MS-grade). The following condition was used for analysis. Column: Zorbax 300-SB C3 (5 μm, 150 x 2.1 mm, 300 Å silica); Flow Rate: 0.6 mL/min; Gradient: 1% B 0-2 min, linearly ramp from 1% B to 91% B 2 to 12 min, 91% B to

95% B 12 to 15 min. Post time is 1% B for 3 min. Flow rate is 0.8 mL/min. MS data was acquired from 4 to 11 min.

1.3.3. Method 3 – 6550-1 5-95 in 10 min

Analysis was performed on an Agilent 1290 Infinity HPLC coupled to an Agilent 6550 Q-TOF with Dual Jet Stream ESI ionization and iFunnel. MS was run in positive ionization mode, extended dynamic range (2 GHz), and low mass range (m/z in range 100 to 1700). The solvent mixtures used were as above. Column: Phenomenex Aeris C4 Widepore (5 μm, 150 x 2.1 mm, 300 Å silica); Flow Rate: 0.1 mL/min; Gradient: 5% B 0-2 min, linearly ramp from 5% B to 95% B 2 to 12 min, 95% B. Post time is 5% B for 3 min. MS data was acquired from 4 to 11 min.

1.3.4. Method 4 – 6550-2 1-91 in 15 min

Analysis was performed on an Agilent 1290 Infinity HPLC coupled to an Agilent 6550 ESI-Q-TOF mass spectrometer. MS was run in positive ionization mode, extended dynamic range (2 GHz), and standard mass range (*m/z* in range 300 to 3000). The solvent mixtures used for LC-MS chromatography were: A = water + 0.1% formic acid (LC-MS-grade), B = acetonitrile + 0.1% formic acid (LC-MS-grade). The following condition was used for analysis. Column: Zorbax 300-SB C3 (5 μm, 150 x 2.1 mm, 300 Å silica); Flow Rate: 0.8 mL/min; Gradient: 1% B 0-2 min, linearly ramp from 1% B to 91% B 2 to 12 min, 91% B to 95% B 12 to 15 min. Post time is 1% B for 3 min. Flow rate is 0.8 mL/min. MS data was acquired from 4 to 11 min.

1.4. Preparative Reverse Phase Purification Methods

1.4.1. Method 1 – 2-Stage Semiprep

Column: Agilent Zorbax 300 SB C3 (5 μm, 9.4 x 250 mm, 300 Å pore size) with an Agilent C3 Zorbax SB 300 guard column.
Loading: < 50 mg
Flow Rate: 4 mL/min.
Column Temperature: 60 °C
Instrument: Agilent mass directed purification system (1260 Infinity LC and 6130 Single Quad MS), affixed with a Timberline Instrument TL105 HPLC column heater. Mobile phases used for LC-MS analysis were Solution A (0.1% v/v TFA in water) and Solution B (0.1% v/v TFA in acetonitrile).

Purification consisted of two stages: gradient generation, and preparative separation.

Gradient Generation

The purification column was equilibrated to 5% B, and an aliquot of the freshly prepared filtered protein solution accounting for approximately 1 mg of crude protein was injected. The column rinsed with 5% B until the denaturing buffer components were rinsed from the column, and the absorbance at 214 nm returned to baseline. The B percentage was then linearly raised from 5% B to 65% B over 60 min. The resulting chromatogram was then analyzed to identify the mobile phase composition that began elution of the desired protein peak (% B can be calculated from the gradient and elution time) – this value is termed “C”.

Preparative Separation

The purification column was equilibrated to 5% B, and the remainder of the filtered protein sample was injected onto the column. The column rinsed with 5% B until the denaturing buffer components were rinsed from the column, and the absorbance at 214 nm returned to baseline. The B percentage was then linearly raised from 5% B to (C - 10) % B at a rate of 1% B/min. The B percentage was then linearly raised from (C - 10) % B to (C + 10) % B over 100 minutes, with 1-minute fractions. The column was then washed with a gradient from (C + 10) % B to 65% B at a rate of 1% B/min.

1.4.2. Method 2 – 2-Stage Prep

Column: Agilent Zorbax 300 SB C3 (5 μm, 21 x 250 mm, 300 Å pore size) with an Agilent C3 Zorbax SB 300 guard column.
Loading: < 250 mg
Flow Rate: 20 mL/min.
Column Temperature: 60 °C
Instrument: Agilent mass directed purification system (1260 Infinity LC and 6130 Single Quad MS), affixed with a Timberline Instrument TL105 HPLC column heater. Mobile phases used for LC-MS analysis were Solution A (0.1% v/v TFA in water) and Solution B (0.1% v/v TFA in acetonitrile).

Purification consisted of two stages: gradient generation, and preparative separation.

Gradient Generation

The purification column was equilibrated to 5% B, and an aliquot of the freshly prepared filtered protein solution accounting for approximately 5 mg of crude protein was injected. The column rinsed with 5% B until the denaturing buffer components were rinsed from the column, and the absorbance at 214 nm retuned to baseline. The B percentage was then linearly raised from 5% B to 65% B over 60 min The resulting chromatogram was then analyzed to identify the mobile phase composition that began elution of the desired protein peak (% B can be calculated from the gradient and elution time) – this value is termed “C”.

Preparative Separation

The purification column was equilibrated to 5% B, and the remainder of the filtered protein sample was injected onto the column. The column rinsed with 5% B until the denaturing buffer components were rinsed from the column, and the absorbance at 214 nm retuned to baseline. The B percentage was then linearly raised from 5% B to (C - 10) % B at a rate of 1% B/min. The B percentage was then linearly raised from (C - 10) % B to (C + 10) % B over 100 min, with 0.75-min fractions. The column was then washed with a gradient from (C + 10) % B to 65% B at a rate of 1% B/min.

1.4.3. Method 3 – 1-Stage Semiprep

Column: Agilent Zorbax 300 SB C3 (5 μm, 9.4 x 150 mm, 300 Å pore size) with an Agilent C3 Zorbax SB 300 guard column.
Loading: < 15 mg
Flow Rate: 8 mL/min.
Column Temperature: 60 °C

Instrument: Agilent mass-directed purification system (1260 Infinity LC and 6130 Single Quad MS), affixed with a Timberline Instrument TL105 HPLC column heater. Mobile phases used for LC-MS analysis were Solution A (0.1% v/v TFA in water) and Solution B (0.1% v/v TFA in acetonitrile).

The purification column was equilibrated to 15% B, and the freshly prepared filtered crude protein solution was injected. The column rinsed with 5% B until the denaturing buffer components were rinsed from the column, and the absorbance at 214 nm retuned to baseline. The B percentage was then linearly raised from 15% B to 65% B over 80 min with 1-min fractions.

1.5. Automated Flow Peptide Synthesis Conditions

Synthesis variables are summarized in the following table:[1]

Parameter	Conditions
Temperature	85-90 °C in reactor, 60 °C in 5' activation loop (for C and H), and 90 °C in 10; activation loop (for all other amino acids)
Flow Rate	40 mL / min
Coupling Step	0.40 M amino acid stock in DMF<br>0.38 M activator stock in DMF<br>Coupling conditions: HATU (13 pump strokes) except S and A with HATU (26 strokes) and C, H, N, Q, V, R, T with PyAOP (26 strokes)
Deprotection Step	40% piperidine in DMF with 2% FA (13 pump strokes)
Washing Steps	DMF (40 pump strokes)

1.6. Determination of Protein Concentration

1.6.1. A_{280} Measurement

All measurements were recorded on a BioTec Epoch instrument with a Take3 Micro-Volume Plate. For each sample or buffer blank to be measured, 2 μL was added to each read position. The sample buffer baseline was recorded as an average of at least 8 spots recorded simultaneously. Solution containing the protein of interest was added to no fewer than 4 spots as 2 μL aliquots, and the absorbance at 280 nm recorded. The molar extinction coefficient of each protein sequence was estimated based on the sequence of the protein via ExPASy Swiss Institute of Bioinformatics Bioinformatics Resource Portal. Protein concentration was calculated using Beer's law.

1.6.2. Bradford Protein Assay

For proteins where the calculated extinction coefficient was too low to provide acceptable signal intensity at the expected protein concentrations, the Quick Start™ Bradford protein assay from BioRad (Catalog number 500-0201) was used. Bovine Serum Albumin standards were prepared according to manufacturer directions. To the bottom of a black, flat bottomed, 96 plate was added 5 μL of the calibrant or solution to be titrated. Each sample was prepared three times on the same plate (3 technical replicates). Working quickly, to each sample was added 250 μL of the working dye solution equilibrated at room temperature. The plate was incubated for 5 min before the absorbance at 495 nm was measured using a BioTec Epoch instrument.

1.7. BCL11a Binding to DNA via TR-FRET

[DNA] (M)	Recombinant BCL11a to D-DNA		Synthetic L-BCL11a to D-DNA		Synthetic D-BCL11a to L-DNA	
	Replicate 1	Replicate 2	Replicate 1	Replicate 2	Replicate 1	Replicate 2
1.00E-06	0.957	1.04	1.09	0.91	0.99	1.01
3.33E-07	0.769	0.778	0.773	0.778	0.817	0.856
1.11E-07	0.495	0.496	0.542	0.555	0.603	0.651
3.70E-08	0.156	0.158	0.219	0.217	0.378	0.362
1.20E-08	0.0275	0.0362	0.151	0.141	0.153	0.155
4.00E-09	0.00618	0.00831	0.0412	0.0404	0.0565	0.052
1.00E-09	-0.00178	0.00209	0.0111	0.0113	0.0172	0.0137
0.00E+00	-0.00051	0.00051	0.00112	-0.00112	0.00087	-0.00087

Supplementary Table 1. Normalized TR-FRET signal for BCL11a binding to DNA.

For TR-FRET measurement of BCL11a binding to DNA, biotinylated, L, D or recombinant BCL11a was diluted to 100 nM, Alexa Fluor™ 488 labeled L- and D-DNA was diluted to 2 µM and Terbium-labeled streptavidin (Cis-Bio) was diluted to 20 nM in assay buffer (10 mM HEPES, 2.5 mM $MgCl_2$, 0.05 mM EDTA, 10% glycerol, 50 mM NaCl, 50 ng/mL poly(dI-dC), 0.1% Triton X-100, 1 mM dithiothreitol, pH 7.5). DNA was serially diluted in assay buffer and 20 µL was added to a black 384-well plate (Costar) containing 20 µL BCL11a protein (final DNA top concentration of 1 µM). Samples were incubated for 30 min at room temperature. TR-FRET signal was determined using a LanthaScreen™ filter on a PheraStar (BMG Biotech) plate reader (Ex: 337 nm; Em1: 490 nM; Em2: 520 nM). The ratio of Em520 to Em490 was calculated, multiplied by 10,000, and plotted against compound concentration. Resulting data was normalized and fit to a 1:1 binding curve.

1.8. CHIP Binding to HSP Peptide via TR-FRET

[HSP Peptide] (M)	Recombinant CHIP to L-P53 Peptide		Synthetic L-CHIP to L-HSP Peptide		Synthetic D-CHIP to D-HSP Peptide	
	Replicate 1	Replicate 2	Replicate 1	Replicate 2	Replicate 1	Replicate 2
1.00E-05	1.03	0.974	1.05	0.947	1.04	0.956
3.33E-06	0.331	0.308	0.385	0.368	0.411	0.43
1.11E-06	0.113	0.0987	0.152	0.155	0.173	0.157
3.70E-07	0.0406	0.037	0.0651	0.0665	0.0782	0.0759
1.23E-07	0.0136	0.0132	0.0285	0.0275	0.0337	0.0303
4.10E-08	0.00441	0.00468	0.012	0.012	0.0118	0.0141
1.40E-08	0.00137	0.00371	0.00471	0.00345	0.00365	0.00656
0.00E+00	-3.34E-06	0.00000334	0.000057	-0.000057	0.0000161	-0.0000161

Supplementary Table 2. Normalized TR-FRET signal for CHIP binding to HSP peptide.

For TR-FRET measurement of CHIP binding to HSP peptide, biotinylated, L, D or recombinant CHIP was diluted to 20 nM, L- and D-FAM-labeled HSP peptide was diluted to 20 µM and Terbium-labeled streptavidin (Cis-Bio) was diluted to 20 nM in assay buffer (10 mM HEPES pH 7.5, 150 mM NaCl, 0.05% Tween 20, 1 mM DTT). HSP peptides were serially diluted in assay buffer (final top concentration of 10 µM) and 20 µL was added to a black 384-well plate (Costar) containing 20 µL of CHIP protein. Samples were incubated for 30 min at room temperature. TR-FRET signal was determined using a LanthaScreen™ filter on a PheraStar (BMG Biotech) plate reader (Ex: 337 nm; Em1: 490 nM; Em2: 520 nM). The ratio of Em520 to Em490 was calculated, multiplied by 10,000, and plotted against compound concentration. Resulting data was normalized and fit to a 1:1 binding curve.

1.9. MDM2 Binding to P53 Peptide via TR-FRET

[P53 Peptide] (M)	Recombinant MDM2 to L-P53 Peptide		Synthetic L-MDM2 to L-P53 Peptide		Synthetic D-MDM2 to D-P53 Peptide	
	Replicate 1	Replicate 2	Replicate 1	Replicate 2	Replicate 1	Replicate 2
1.00E-06	1.01	0.989	0.837	0.856	1.06	0.944
3.33E-07	0.848	0.879	1.01	0.988	0.949	0.94
1.11E-07	0.707	0.769	0.893	0.89	0.77	0.84
3.70E-08	0.682	0.672	0.82	0.825	0.727	0.734
1.20E-08	0.565	0.558	0.705	0.718	0.578	0.561
4.00E-09	0.378	0.381	0.319	0.295	0.213	0.19
1.00E-09	0.191	0.19	0.0852	0.0842	0.0527	0.0478
0.00E+00	-0.00208	0.00208	0.000229	-0.000229	0.000353	-0.000353

Supplementary Table 3. Normalized TR-FRET signal for MDM2 binding to P53 peptide.

For TR-FRET measurement of MDM2 binding to P53 peptide, biotinylated, L, D or recombinant MDM2 was diluted to 20 nM, L- and D-FAM-labeled P53 peptide was diluted to 2 µM and Terbium-labeled streptavidin (Cis-Bio) was diluted to 20 nM in assay buffer (10 mM HEPES pH 7.5, 150 mM NaCl, 0.05% Tween 20, 1 mM DTT). P53 peptides were serially diluted in assay buffer (final top concentration of 1 µM) and 20 µL was added to a black 384-well plate (Costar) containing 20 µL of MDM2 protein. Samples were incubated for 30 minutes at room temperature. TR-FRET signal was determined using a LanthaScreen™ filter on a PheraStar (BMG Biotech) plate reader (Ex: 337 nm; Em1: 490 nM; Em2: 520 nM). The ratio of Em520 to Em490 was calculated, multiplied by 10,000, and plotted against compound concentration. Resulting data was normalized and fit to a 1:1 binding curve.

1.10. Max-Max and Myc-Max Binding to DNA via TR-FRET

[DNA] (M)	Recombinant-Myc-Max to D-DNA		Synthetic L-Myc-Max to D-DNA		Synthetic D-Myc-Max to L-DNA	
	Replicate 1	Replicate 2	Replicate 1	Replicate 2	Replicate 1	Replicate 2
3.00E-06	0.928	1.07	0.927	1.07	0.928	1.07
1.00E-06	0.987	0.892	0.727	0.671	0.791	0.76
3.33E-07	0.67	0.708	0.534	0.578	0.561	0.601
1.11E-07	0.479	0.466	0.422	0.349	0.43	0.373
3.70E-08	0.226	0.245	0.203	0.197	0.21	0.201
1.20E-08	0.0834	0.0992	0.0842	0.0884	0.0825	0.0883
4.00E-09	0.0266	0.0335	0.0292	0.0328	0.0308	0.0342
0.00E+00	0.0022	-0.0022	-0.000192	0.000192	0.000293	-0.000293

Supplementary Table 4. Normalized TR-FRET signal for Myc-Max binding to DNA.

[DNA] (M)	Recombinant Max-Max to D-DNA		Synthetic L-Max-Max to D-DNA		Synthetic D-Max-Max to L-DNA	
	Replicate 1	Replicate 2	Replicate 1	Replicate 2	Replicate 1	Replicate 2
1.00E-06	1.09	0.911	0.992	1.01	1.03	0.974
3.33E-07	0.543	0.603	0.708	0.687	0.674	0.69
1.11E-07	0.282	0.285	0.464	0.454	0.383	0.394
3.70E-08	0.139	0.174	0.257	0.246	0.19	0.2
1.23E-08	0.0588	0.0724	0.124	0.0875	0.0948	0.0747
4.12E-09	0.0217	0.0206	0.052	0.0475	0.0374	0.0422
1.37E-09	0.0313	0.00322	0.0253	0.0138	0.0176	0.00563
0.00E+00	-0.00766	0.00766	0.0018	-0.0018	-0.00931	0.00931

Supplementary Table 5. Normalized TR-FRET signal for Max-Max binding to DNA.

For TR-FRET measurement of Myc-Max and Max-Max binding to E-box DNA, biotinylated, L, D or recombinant Myc-Max or Max-Max was diluted to 20 nM, Alexa Fluor™ 488 labeled L- and D-DNA was diluted to 6 µM and Terbium-labeled streptavidin (Cis-Bio) was diluted to 20 nM in assay buffer (10 mM HEPES, 2.5 mM $MgCl_2$, 0.05 mM EDTA, 10% glycerol, 50 mM NaCl, 50 ng/mL poly(dI-dC), 0.1% Triton X-100, 1 mM dithiothreitol, pH 7.5). DNA was serially diluted in assay buffer and 20 µL was added to a black 384-well plate (Costar) containing 20 µL Myc/Max or Max/Max protein (final DNA top concentration of 3 µM). Samples were incubated for 30 minutes at room temperature. TR-FRET signal was determined using a LanthaScreen™ filter on a PheraStar (BMG Biotech) plate reader (Ex: 337 nm; Em1: 490 nM; Em2: 520 nM). The ratio of Em520 to Em490 was calculated, multiplied by 10,000, and plotted against compound concentration. Resulting data was normalized and fit to a 1:1 binding curve.

1.11. Barnase Catalysis of RNA Substrate via FRET

The relative catalytic activity of synthetic L- and D-Barnase was measured in a similar manner to previous work.[2] A fluorogenic substrate of the appropriate chirality (6-FAM-$dA^{D}rG^{D}dA^{D}dA^{D}$-6-TAMRA for L-barnase, and 6-FAM-$dA^{L}rG^{L}dA^{L}dA^{L}$-6-TAMRA for D-barnase) undergoes an increase in fluorescence intensity upon cleavage at the rG-dA junction by barnase. The resulting time course of fluorescence growth can be fit to a first order rate equation to extract observed k_{cat}/K_M values. Both fluorogenic substrates (6-FAM-$dA^{D}rG^{D}dA^{D}dA^{D}$-6-TAMRA for L-barnase, and 6-FAM-$dA^{L}rG^{L}dA^{L}dA^{L}$-6-TAMRA for D-barnase) for the assay was purchased from ChemGenes as HPLC-purified solid and dissolved in assay buffer solution (100 mM MES, 100 mM NaCl, pH 6.0). Fluorescence measurements were recorded on a Tecan Spark® plate reader with excitation at 495 nm and monitoring emission at 515 nm. Assays were carried out in flat bottomed, black 96-well plates. The fluorogenic substrate of the appropriate chirality was diluted in assay buffer, and the baseline absorbance recorded for at least 150 sec. To initiate hydrolysis, L or D barnase was added in a single aliquot and the fluorescence monitored every 15 s until sufficient data was collected for analysis. Reactions were carried out in a total of 250 µL with 200 nM substrate, and 2 nM barnase. The experiment was performed in duplicate. Blank runs were performed in parallel with the experimental runs.

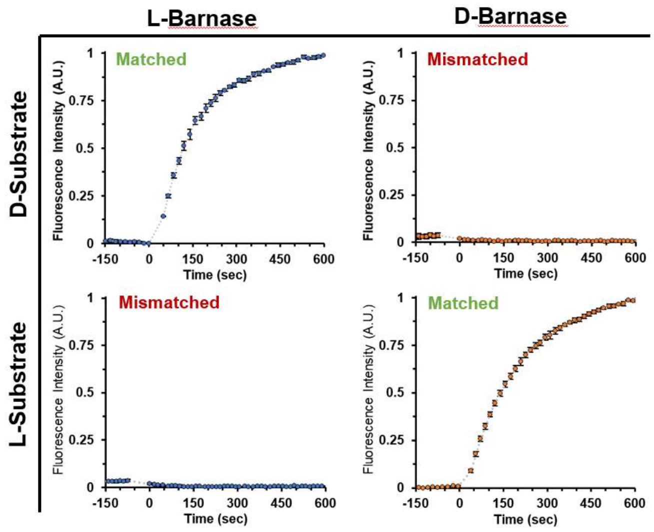

Supplementary Figure 1. Raw time course measurement data for the fluorogenic barnase activity assays. Error bars are plotted as SD.

**L-Barnase**						**D-Barnase**					
**L-Substrate**			**D-Substrate**			**L-Substrate**			**D-Substrate**		
**t (s)**	**Mean**	**SD**	**T (s)**	**Mean**	**SD**	**t (s)**	**Mean**	**SD**	**t (s)**	**Mean**	**SD**
-426.468	0.027	0.001	-183.389	0.030	0.002	-221.057	0.014	0.015	-426.468	0.118	0.018
-411.632	0.026	0.001	-165.393	0.021	0.003	-204.640	0.005	0.010	-411.632	0.028	0.019
-396.771	0.029	0.002	-149.154	0.013	0.003	-188.408	0.005	0.004	-396.771	0.032	0.015
-383.786	0.032	0.006	-132.930	0.014	0.010	-172.179	0.004	0.005	-383.786	0.030	0.015
-370.817	0.027	0.004	-113.866	0.010	0.011	-155.798	0.001	0.002	-370.817	0.027	0.017
-357.828	0.028	0.006	-97.482	0.010	0.004	-139.567	0.001	0.003	-357.828	0.027	0.017
-344.823	0.027	0.003	-81.188	0.008	0.006	-123.370	0.000	0.002	-344.823	0.027	0.015
-331.795	0.028	0.004	-64.988	0.006	0.009	-107.156	0.001	0.001	-331.795	0.027	0.012
-316.803	0.030	0.002	-48.775	0.009	0.005	-90.951	0.005	0.002	-316.803	0.030	0.014
-303.813	0.032	0.003	-32.544	0.006	0.003	-74.680	0.002	0.004	-303.813	0.028	0.015
-288.706	0.029	0.003	-16.251	0.000	0.003	-58.414	0.005	0.003	-288.706	0.029	0.016
-273.731	0.032	0.005	0.000	0.000	0.002	-40.000	0.006	0.003	-273.731	0.032	0.019
-260.788	0.032	0.003	49.496	0.141	0.002	-21.653	0.010	0.002	-260.788	0.034	0.017
-243.888	0.028	0.004	66.586	0.250	0.010	-5.471	0.007	0.005	-243.888	0.027	0.012
-230.361	0.031	0.004	85.235	0.357	0.014	38.839	0.089	0.008	-230.361	0.029	0.015
-217.364	0.032	0.006	103.611	0.434	0.017	56.000	0.179	0.013	-217.364	0.030	0.013
-204.364	0.031	0.004	119.893	0.513	0.022	72.234	0.259	0.014	-204.364	0.030	0.010
-191.399	0.031	0.005	140.626	0.573	0.026	88.447	0.324	0.015	-191.399	0.033	0.016
-178.367	0.032	0.004	158.709	0.646	0.021	104.696	0.386	0.013	-178.367	0.031	0.017
-165.328	0.032	0.003	179.439	0.668	0.021	121.051	0.445	0.015	-165.328	0.032	0.015
-152.332	0.033	0.005	195.666	0.710	0.023	139.015	0.497	0.016	-152.332	0.034	0.013
-139.326	0.031	0.004	211.910	0.738	0.017	157.058	0.546	0.017	-139.326	0.032	0.016
-126.278	0.032	0.003	228.227	0.764	0.020	175.101	0.586	0.016	-126.278	0.030	0.010
-113.248	0.033	0.007	244.502	0.790	0.014	191.346	0.628	0.015	-113.248	0.031	0.014
-100.233	0.034	0.007	260.808	0.804	0.007	209.863	0.664	0.020	-100.233	0.038	0.012
-87.215	0.033	0.005	277.115	0.822	0.009	226.206	0.699	0.015	-87.215	0.032	0.012
-74.172	0.035	0.006	293.451	0.832	0.012	242.451	0.725	0.013	-74.172	0.038	0.013
0.000	0.018	0.005	309.667	0.857	0.010	260.865	0.748	0.015	0.000	0.019	0.002
16.636	0.015	0.004	326.059	0.854	0.012	277.202	0.762	0.013	16.636	0.013	0.006
29.823	0.013	0.004	342.317	0.866	0.012	293.592	0.792	0.016	29.823	0.013	0.008
45.792	0.012	0.003	358.705	0.889	0.012	309.923	0.804	0.020	45.792	0.012	0.005
63.259	0.007	0.002	377.513	0.892	0.011	328.054	0.827	0.017	63.259	0.010	0.004
76.362	0.010	0.005	393.915	0.904	0.004	344.402	0.841	0.013	76.362	0.014	0.003
89.470	0.008	0.003	410.330	0.908	0.008	360.814	0.857	0.008	89.470	0.011	0.005
102.718	0.008	0.005	426.644	0.928	0.004	377.078	0.870	0.008	102.718	0.011	0.002
117.900	0.004	0.003	442.988	0.936	0.012	393.460	0.881	0.013	117.900	0.004	0.001
131.872	0.004	0.003	459.364	0.940	0.007	409.958	0.886	0.012	131.872	0.010	0.000
145.105	0.003	0.002	475.823	0.948	0.006	426.566	0.902	0.012	145.105	0.005	0.003
158.836	0.006	0.003	494.625	0.953	0.010	442.926	0.913	0.011	158.836	0.008	0.003
171.955	0.005	0.002	512.269	0.961	0.008	459.566	0.925	0.009	171.955	0.006	0.003
188.720	0.004	0.002	531.111	0.979	0.009	475.912	0.935	0.008	188.720	0.009	0.006
201.843	0.004	0.003	547.465	0.972	0.005	492.350	0.945	0.006	201.843	0.010	0.002
214.917	0.004	0.002	566.153	0.976	0.009	511.040	0.956	0.012	214.917	0.007	0.000
228.057	0.003	0.002	582.623	0.980	0.009	527.681	0.957	0.009	228.057	0.012	0.003
241.318	0.004	0.002	599.020	0.986	0.003	544.146	0.965	0.004	241.318	0.009	0.003
254.527	0.005	0.001	615.545	0.992	0.002	560.545	0.969	0.010	254.527	0.004	0.003
267.665	0.006	0.000	632.306	0.991	0.013	576.936	0.986	0.003	267.665	0.006	0.002
280.981	0.007	0.001	648.728	1.000	0.003	593.302	0.984	0.004	280.981	0.006	0.003
294.056	0.002	0.002	665.120	1.000	0.007	609.689	0.987	0.011	294.056	0.006	0.002
307.213	0.003	0.002	681.651	0.999	0.011	626.580	1.000	0.005	307.213	0.003	0.003
324.741	0.005	0.002	698.066	1.003	0.013	643.081	1.006	0.004	324.741	0.012	0.003
337.942	0.004	0.002	714.529	1.009	0.013	659.607	1.010	0.006	337.942	0.010	0.002
353.025	0.003	0.003	731.121	1.017	0.005	676.232	1.020	0.002	353.025	0.009	0.003
369.192	0.006	0.003	n.d.	n.d.	n.d.	n.d.	n.d.	n.d.	369.192	0.009	0.002
382.349	0.005	0.003	n.d.	n.d.	n.d.	n.d.	n.d.	n.d.	382.349	0.009	0.004
395.516	0.004	0.002	n.d.	n.d.	n.d.	n.d.	n.d.	n.d.	395.516	0.007	0.002

412.313	0.004	0.003	n.d.	n.d.	n.d.	n.d.	n.d.	n.d.	412.313	0.005	0.002
425.436	0.006	0.003	n.d.	n.d.	n.d.	n.d.	n.d.	n.d.	425.436	0.006	0.001
440.949	0.005	0.002	n.d.	n.d.	n.d.	n.d.	n.d.	n.d.	440.949	0.008	0.003
454.055	0.006	0.004	n.d.	n.d.	n.d.	n.d.	n.d.	n.d.	454.055	0.005	0.003
467.178	0.004	0.001	n.d.	n.d.	n.d.	n.d.	n.d.	n.d.	467.178	0.006	0.003
480.358	0.004	0.001	n.d.	n.d.	n.d.	n.d.	n.d.	n.d.	480.358	0.010	0.001
493.561	0.008	0.003	n.d.	n.d.	n.d.	n.d.	n.d.	n.d.	493.561	0.006	0.004
506.672	0.005	0.001	n.d.	n.d.	n.d.	n.d.	n.d.	n.d.	506.672	0.005	0.002
519.870	0.006	0.000	n.d.	n.d.	n.d.	n.d.	n.d.	n.d.	519.870	0.011	0.000
533.089	0.005	0.002	n.d.	n.d.	n.d.	n.d.	n.d.	n.d.	533.089	0.008	0.001
546.211	0.003	0.001	n.d.	n.d.	n.d.	n.d.	n.d.	n.d.	546.211	0.007	0.006
559.438	0.005	0.002	n.d.	n.d.	n.d.	n.d.	n.d.	n.d.	559.438	0.008	0.003
572.716	0.006	0.001	n.d.	n.d.	n.d.	n.d.	n.d.	n.d.	572.716	0.009	0.003
587.317	0.005	0.001	n.d.	n.d.	n.d.	n.d.	n.d.	n.d.	587.317	0.005	0.001
600.570	0.008	0.001	n.d.	n.d.	n.d.	n.d.	n.d.	n.d.	600.570	0.010	0.003
615.698	0.008	0.003	n.d.	n.d.	n.d.	n.d.	n.d.	n.d.	615.698	0.005	0.001
631.188	0.008	0.002	n.d.	n.d.	n.d.	n.d.	n.d.	n.d.	631.188	0.008	0.002
648.861	0.005	0.004	n.d.	n.d.	n.d.	n.d.	n.d.	n.d.	648.861	0.009	0.002
666.454	0.010	0.001	n.d.	n.d.	n.d.	n.d.	n.d.	n.d.	666.454	0.008	0.002
679.545	0.010	0.003	n.d.	n.d.	n.d.	n.d.	n.d.	n.d.	679.545	0.007	0.001
692.819	0.008	0.002	n.d.	n.d.	n.d.	n.d.	n.d.	n.d.	692.819	0.009	0.003
705.939	0.010	0.001	n.d.	n.d.	n.d.	n.d.	n.d.	n.d.	705.939	0.008	0.004
719.131	0.007	0.003	n.d.	n.d.	n.d.	n.d.	n.d.	n.d.	719.131	0.006	0.000
732.281	0.007	0.002	n.d.	n.d.	n.d.	n.d.	n.d.	n.d.	732.281	0.009	0.002
745.434	0.007	0.003	n.d.	n.d.	n.d.	n.d.	n.d.	n.d.	745.434	0.007	0.002
758.543	0.008	0.001	n.d.	n.d.	n.d.	n.d.	n.d.	n.d.	758.543	0.007	0.004
771.719	0.008	0.003	n.d.	n.d.	n.d.	n.d.	n.d.	n.d.	771.719	0.006	0.003
785.040	0.006	0.001	n.d.	n.d.	n.d.	n.d.	n.d.	n.d.	785.040	0.009	0.002
800.502	0.006	0.002	n.d.	n.d.	n.d.	n.d.	n.d.	n.d.	800.502	0.004	0.001
813.681	0.005	0.003	n.d.	n.d.	n.d.	n.d.	n.d.	n.d.	813.681	0.010	0.003
826.847	0.008	0.001	n.d.	n.d.	n.d.	n.d.	n.d.	n.d.	826.847	0.011	0.003

Supplementary Table 6. Averaged data from fluorogenic RNase activity assay was used for the determination of k_{cat}/K_M values.

1.12. ERG and IRAK2 Circular Dichroism

Before analysis, all proteins were exchanged into CD buffer (10 mM KP*i*, 5% glycerol v/v, pH 7.4). Fast protein liquid chromatography (FPLC) purification was performed using GE Life Sciences columns on an ÄKTA Pure FPLC system. The ÄKTA Pure, FPLC columns, and all buffers were stored and subsequently used in a cold room maintained at 4 °C. HiTrap™ Desalting 5 mL: Before desalting, the column was equilibrated with 20 mL of the CD buffer at 2 mL/min or until the conductance reading from the ÄKTA Pure stabilizes, whichever is the greater volume. Up to 1.5 mL of sample was loaded into the column using a syringe fitted with a compatible male-end screw tip, and then the loaded column was attached to the ÄKTA Pure. An isocratic flow of buffer (2 CV) at 1 mL/min provided the desalted, buffer-exchanged protein. The concentration of the resulting protein solution was determined with ***Method 1.6.1***. Protein concentration was adjusted to 0.1 mg/mL with additions of CD buffer. All CD spectra were recorded on an AVIV Model 420 Circular Dichroism Spectrometer, with a 1 mm quartz cuvette (Hellma Analytics, Article No.: 100-1-40). Before all readings, cuvettes were cleaned with Hellmanex™ 3 concentrate according to manufacturer directions. Far circular dichroism spectra were recorded as the average of three scans between 190 nm to 260 nm with 1 nm steps, 2 nm scanning windows, and a 3 s averaging time for each scan at 25 °C. For variable temperature CD, CD signal was monitored at 225 nm with 2 nm bandwidth and 8 second averaging time. Spectra were recorded between 0 and 110 °C with 5 °C steps and 2-minute incubation time at each step.

1.13. NEMO binding to IKKb peptide with SPR

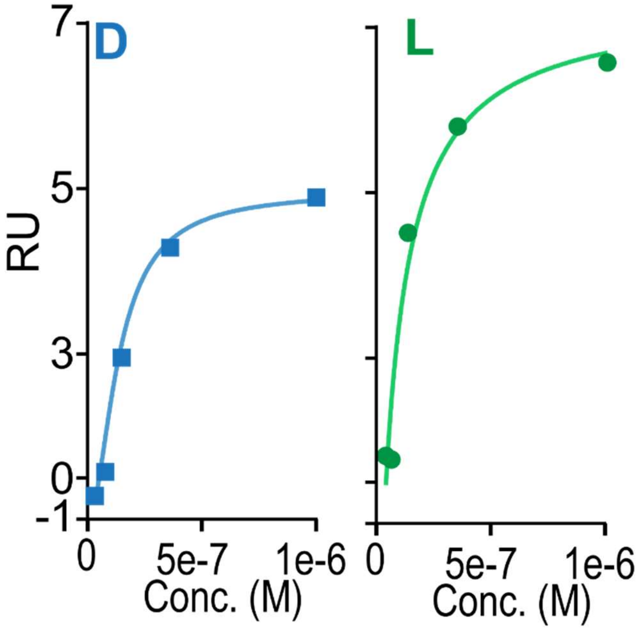

Supplementary Figure 2. SPR of Synthetic L, D and Recombinant NEMO to IKKB peptide. Sensorgrams of IKKB peptide binding to immobilized NEMO are shown.

SPR experiments were performed on a Biacore S200 (Cytiva) instrument at 25 °C in 1x HBS-P+ buffer (Cytiva) with 1% DMSO and 0.5 mM TCEP. A CAP Series S sensor chip was docked and rehydrated overnight with water. On the day of the experiment, the instrument was primed with running buffer. CAP reagent was diluted 5X in water and injected over the CAP surface at 2 µL/min for 120 seconds for a final capture level of ~200 RU. Synthetic L, D, or recombinant NEMO was diluted to 200 nM in running buffer and captured to channels 2, 3, or 4, respectively, at 5 µL/min for 60 seconds for a final immobilization level of ~30 RU. The iKKB peptide was diluted to 1 µM in running buffer then serially diluted 3-fold for a total of 8 concentrations with one blank. Compounds were injected over the immobilized and reference surfaces at 30 µL/min for 180 seconds and then allowed to dissociate for 360 seconds. The surface was regenerated with CAP regeneration solution at 10 µL/min for 120 seconds. Sensorgrams were double-referenced and fit to a 1:1 steady state affinity model.

1.14. FKBP12 binding to Rapamycin/mTor with SPR

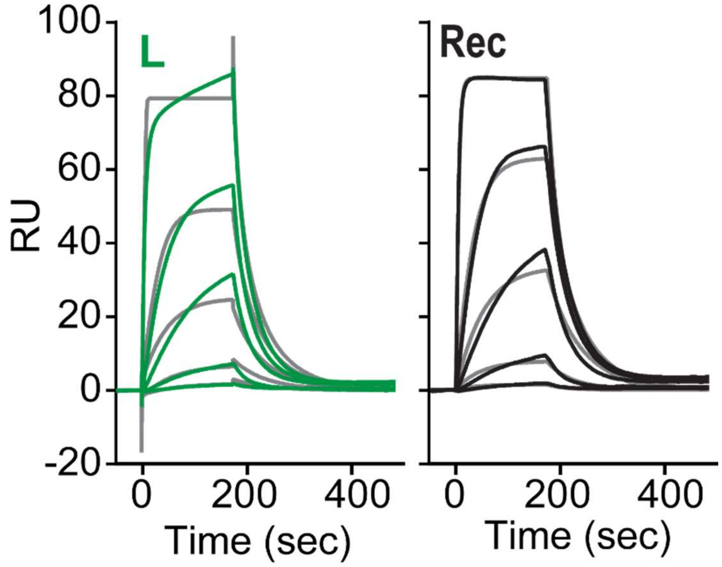

Supplementary Figure 3. SPR of Synthetic L and Recombinant FKBP12 to Rapamycin/mTOR. Sensorgrams of Rapamycin/mTOR binding to immobilized FKBP12 are shown.

SPR experiments were performed on a Biacore S200 (Cytiva) instrument at 25 °C in 1x HBS-P+ buffer (Cytiva) with 1% DMSO and 0.5 mM TCEP. A CAP Series S sensor chip was docked and rehydrated overnight with water. On the day of the experiment, the instrument was primed with running buffer. CAP reagent was diluted 5X in water and injected over the CAP surface at 2 µL/min for 120 seconds for a final capture level of ~200 RU. Synthetic L- and recombinant FKBP12 were diluted to 200 nM in running buffer and captured to channels 2, or 4, respectively, at 5 µL/min for 60 seconds for a final immobilization level of ~200 RU. The Rapamycin and mTOR were each diluted to a final concentration of 500 nM in running buffer then serially diluted 3-fold for a total of 8 concentrations with one blank. The complex was injected over the immobilized and reference surfaces at 30 µL/min for 180 seconds and then allowed to dissociate for 360 seconds. The surface was regenerated with CAP regeneration solution at 10 µL/min for 120 seconds. Sensorgrams were double-referenced and fit to a 1:1 binding model.

1.15. YAP1 binding to Dendrin with SPR

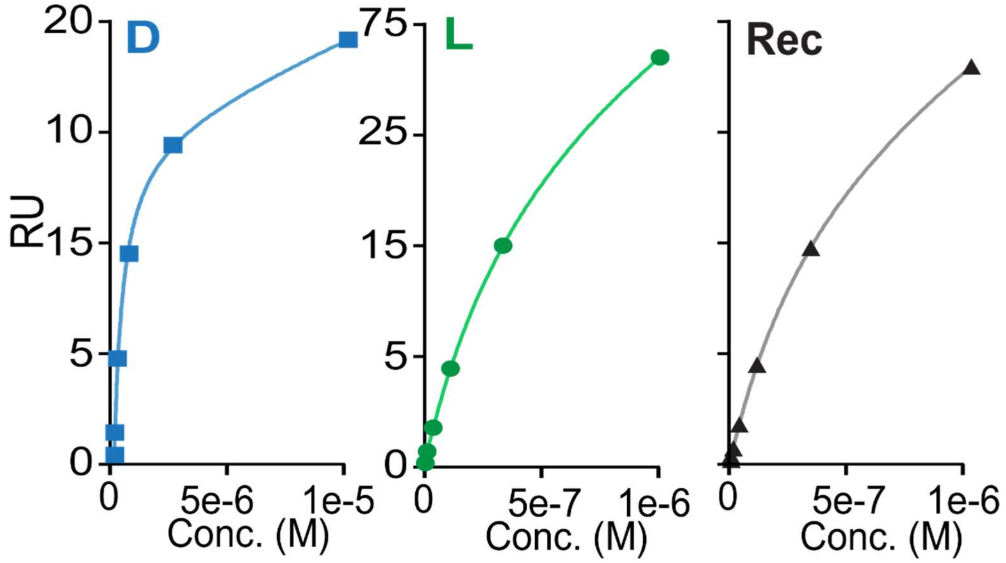

Supplementary Figure 4. SPR of Synthetic L, D and Recombinant YAP1 to Dendrin peptide. Sensorgrams of Dendrin peptide binding to immobilized YAP1 are shown.

SPR experiments were performed on a Biacore S200 or 8K (Cytiva) instrument at 25 °C in 1x HBS-P+ buffer (Cytiva) with 1% DMSO and 0.5 mM TCEP using the CAP system (D-YAP1) or SA capture (L and recombinant YAP1). For CAP experiment, a CAP Series S sensor chip was docked and rehydrated overnight with water. On the day of the experiment, the instrument was primed with running buffer. CAP reagent was diluted 5X in water and injected over the CAP surface at 2 µL/min for 120 seconds for a final capture level of ~200 RU. Synthetic D- and recombinant YAP1 were diluted to 400 nM in running buffer and captured to channels 2, and 4, respectively, at 5 µL/min for 60 seconds for a final immobilization level of ~170 RU. L- and D-Dendrin peptide were each diluted to 10 µM in running buffer then serially diluted 4-fold for a total of 6 concentrations with one blank. The peptides were injected over the immobilized and reference surfaces at 30 µL/min for 180 seconds and then allowed to dissociate for 360 seconds. The surface was regenerated with CAP regeneration solution at 10 µL/min for 120 seconds. Sensorgrams were double-referenced and fit to a 1:1 steady state affinity model. For SA experiments, a SA Series S sensor chip was docked and pre-conditioned with 3 injections of 50 mM NaOH/1 M NaCl to remove unbound streptavidin from the surface. Synthetic L, or recombinant YAP1 was diluted to 1 µg/mL in running buffer and immobilized to channels 1 and 2, respectively, at 1 µL/min for 300 seconds for a final immobilization level of ~1500 RU. L-Dendrin peptide was diluted to 10 µM in running buffer then serially diluted 3-fold for a total of 7 concentrations with one blank. The peptide was injected over the immobilized and reference surfaces at 30 µL/min for 180 seconds and then allowed to dissociate for 360 seconds. The surface was regenerated with a 15 second injection of 1 M sodium chloride. Sensorgrams were double-referenced and fit to a 1:1 steady state affinity model.

1.16. NEMO_iZIP binding to IKKb with SPR

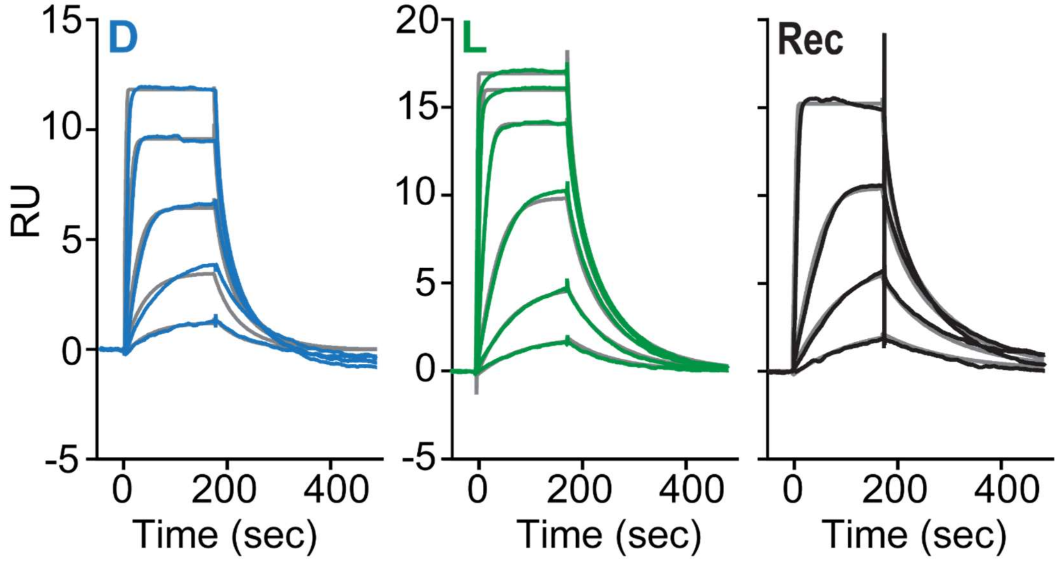

Supplementary Figure 5. SPR of Synthetic L, D and Recombinant NEMO_iZIP SPR to IKKB peptide. Sensorgrams of IKKB peptide binding to immobilized NEMO_iZIP are shown.

SPR experiments were performed on a Biacore S200 (Cytiva) instrument at 25 °C in 1x HBS-P+ buffer (Cytiva) with 1% DMSO and 0.5 mM TCEP. A CAP Series S sensor chip was docked and rehydrated overnight with water. On the day of the experiment, the instrument was primed with running buffer. CAP reagent was diluted 5X in water and injected over the CAP surface at 2 µL/min for 120 seconds for a final capture level of ~200 RU. Synthetic L- and recombinant FKBP12 were diluted to 200 nM in running buffer and captured to channels 2, 3 and 4, respectively, at 5 µL/min for 60 seconds for a final immobilization level of ~60 RU. L- and D-IKKB peptides were diluted to 500 nM in running buffer then serially diluted 3-fold for a total of 8 concentrations with one blank. The peptides were injected over the immobilized and reference surfaces at 30 µL/min for 180 seconds and then allowed to dissociate for 360 seconds. The surface was regenerated with CAP regeneration solution at 10 µL/min for 120 seconds. Sensorgrams were double-referenced and fit to a 1:1 binding model.

1.17. MDM2 Protein Expression

For protein used in phage screens and biochemical assays: MDM2 (residues 25-109) with an N-terminal 6xHis–yBBr–TEV tag was recombinantly expressed in *E. coli* BL21 CodonPlus cells (Agilent) from pET-derived expression vectors (Novagen). The cells were induced at OD600 = 0.6 with 1 mM isopropyl β-D-1-thiogalactopyranoside (IPTG) for 4 h at 37 °C, then harvested and resuspended in 25 mM Tris-HCl pH 7.5, 300 mM NaCl, 10% glycerol, 1 mM PMSF. For purification, the pellet was lysed with a tip sonicator, and pelleted at 22,000 x g for 30 min at 4 °C. The pellets were washed with 20 mM Tris-HCl pH 8.0, 150 mM NaCl, 1 M urea, 1.0% triton X-100 three times, and the inclusion body was dissolved in in 20 mM Tris-HCl pH 8.0, 150 mM NaCl, 8 M urea, and 2 mM ß-mercaptoethanol (ß-me). The supernatant was purified with Ni-NTA resin (Qiagen), eluting with 20 mM Tris-HCl pH 8.0, 150 mM NaCl, 8 M urea, 2 mM ß-me, and 250 mM imidazole. Protein elutes were diluted to ~ 0.1 mg/mL and dialyzed into buffers containing 10 mM

Tris-HCl pH 8.0, 150 mM NaCl, 2 mM β-me, with 4, 2 ,1, or 0 M urea, at 4 °C for 8 h for each urea gradient. Urea-free proteins were concentrated with Amicon spin filters (Millipore Sigma) to ~1 mg/mL and biotinylated via the yBBr reaction according to standard procedures.[3] Biotinylated proteins were pooled, concentrated, and loaded onto a Superdex™ 10/300 75 pg (Cytiva) SEC column and eluted in 20 mM HEPES pH 7.0, 200 mM NaCl, 5% glycerol, 0.5 mM tris(2-carboxyethyl) phosphine (TCEP). Fractions containing pure protein were collected, pooled, concentrated to ~1 mg/mL and stored at −80°C.

For protein used in crystallography: MDM2 (residues 17-111, C17S) with an N-terminal 6xHis–TEV tag was recombinantly expressed in *E. coli* BL21 (DE3) cells (Agilent) from pET-derived expression vectors (Novagen). The cells were induced at OD600 = 0.6 with 0.15 mM IPTG for 16 h at 37 °C, then harvested and resuspended in 50 mM Tris, pH 8.0, 200 mM NaCl, 10% glycerol, 1 mM TCEP, and 20 mM imidazole. For purification, the pellet was lysed with a tip sonicator with power level set at 200 W, 3 seconds on and 3 seconds off for 20 min, pelleted at 22,000 x g for 30 min at 4 °C. The supernatant was purified with Ni-NTA resin (Qiagen), eluting with 50 mM Tris, pH 8.0, 200 mM NaCl, 10 % glycerol, 1 mM TCEP, and 250 mM imidazole. Eluted proteins were pooled, concentrated, and TEV-cleaved by adding TEV protease at a ratio of 1:10 protease to protein and incubated overnight at 4 °C. TEV-cleaved proteins were loaded onto a Superdex™ 10/300 75 pg (Cytiva) SEC column and eluted in 50 mM Tris, pH 8.0, 200 mM NaCl, 10% glycerol, and 1 mM TCEP. Fractions containing pure protein were collected, pooled, concentrated to 8 mg/mL and stored at −80 °C.

1.18. ERG Protein Expression

ERG (115-208) with an N-terminal 6xhis-TEV-yBBR-Thrombin tag was recombinantly expressed in One Shot™ BL21 Star™ (DE3) Chemically Competent *E. coli* (ThermoFisher) from pET-derived expression vectors (ThermoFisher) in Luria broth supplemented with 0.1 mg/mL ampicillin. The cells were induced at OD600 = 0.8 with 1 mM isopropyl β-D-1-thiogalactopyranoside (IPTG) for 8 h at 25 °C then overnight at 15 °C, then harvested and resuspended in lysis buffer (50 mM HEPES, 200 mM NaCl, 10 mM imidazole, 0.5 mM TCEP, 2 mM ATP, 10 mM MgCl2, 1% NP40, 5% glycerol, Roche complete inhibitors, pH 7.5). For purification, the pellet was lysed with a tip sonicator then pelleted at 20,000 x g for 30 min at 4 °C. Supernatant was collected and purified with cOmplete™ His-Tag Purification Columns (Roche) eluting with 300 mM imidazole. Protein was TEV cleaved by adding TEV protease at a ratio of 1:10 protease to protein and incubated overnight at 25 °C. For biochemical assays and phage screening, cleaved protein was then biotin labeled via yBBR according to established methods.[3] Cleaved protein was then concentrated and injected onto a Superdex® 75 10/300 GL gel filtration column equilibrated with formulation buffer (50 mM HEPES, 200 mM NaCl, 0.5 mM TCEP, 5% glycerol, pH 7.5). Protein fractions were collected, concentrated, aliquoted and frozen.

1.19. IRAK2 Protein Expression

IRAK2 Death domain (residues 2-96, C3S) with an N-terminal 6xHis–MBP–3C–YBBR–TEV tag was recombinantly expressed in *E. coli* BL21 CodonPlus RIPL cells (Agilent) from pET-derived expression vectors (Novagen). The cells were induced at OD600 = 0.6 with 0.15 mM IPTG for 16 hours at 16°C, then harvested and resuspended in 20 mM Tris pH 8.0, 200 mM NaCl, 5% glycerol, and 20 mM imidazole. For purification, the pellet was lysed with a tip sonicator and pelleted at 22,000 x g for 30 minutes at 4 °C. The supernatant was purified with Ni-NTA resin (Qiagen), eluted with 20 mM Tris, pH 8.0, 200 mM NaCl, 5 % glycerol, and 250 mM imidazole. Protein was

3C-cleaved by adding Pierce™ HRV 3C protease (ThermoFisher) at a ratio of 1:10 protease to protein and incubated for 4 hours at 4 °C, and biotinylated via the yBBr reaction according to standard procedures.[3] Biotinylated proteins were diluted into buffer 5 mM Tris, pH 8.0, 10% glycerol, and 1 mM DTT, and loaded onto a Q-HP (Cytiva) column. Proteins were eluted with a gradient from 50 mM to 1000 mM NaCl. Protein elutes were pooled, concentrated, and loaded onto a Superdex™ 10/300 75 pg (Cytiva) SEC column, and eluted in 25 mM Tris pH 8.0, 200 mM NaCl, 5% glycerol, and 1mM TCEP. Fractions containing pure protein were collected, pooled, concentrated to ~8 mg/mL and stored at −80 °C.

1.20. CHIP Protein Expression

For protein used in phage screen and biochemical assays: CHIP (STUB1, residues 23-154) with an N-terminal 6xHis–yBBr–TEV tag was recombinantly expressed in *E. coli* BL21 CodonPlus cells (Agilent) from pET-derived expression vectors (Novagen). The cells were induced at OD600 = 0.6 with 1 mM IPTG for 4 hours at 37 °C, then harvested and resuspended in 50 mM Tris-HCl pH 8.0, 500 mM NaCl ,10 mM imidazole, 10% glycerol, and 10 mM ß-me. For purification, the pellet was lysed with a tip sonicator and pelleted at 22,000 x g for 30 minutes at 4 °C. The supernatant was purified with Ni-NTA resin (Qiagen), eluted with 50 mM Tris-HCl pH 8.0, 500 mM NaCl, 10% glycerol, 10 mM ß-me and 250 mM imidazole, and biotinylated via the yBBr reaction according to standard procedures.[3] Biotinylated proteins were pooled, concentrated, and loaded onto a Superdex™ 10/300 75 pg (Cytiva) SEC column, and eluted in 20 mM HEPES pH 7.0, 150 mM NaCl, 10% glycerol, 2 mM DTT. Fractions containing pure protein were collected, pooled, concentrated to ~1.2 mg/mL and stored at −80°C.

For protein used in competition FP and crystallography: CHIP (residues 23-303 for competition FP and 23–154 for crystallography) with an N-terminal 6xHis–TEV tag was recombinantly expressed in *E. coli* BL21 CodonPlus cells (Agilent) from a pET21b-derived expression vector (Novagen). The cells were induced at OD600 = 0.6 with 1 mM IPTG for 4 h at 37 °C, then harvested and resuspended in 50 mM Tris-HCl pH 8.0, 500 mM NaCl,10 mM imidazole, 10% glycerol, and 10 mM ß-me. For purification, the pellet was lysed with a tip sonicator with power level set at 400 W, 3 seconds on and 3 seconds off for 20 min, pelleted at 22,000 x g for 30 min at 4 °C. The supernatant was purified with Ni-NTA resin (Qiagen), eluting with 250 mM imidazole. Eluted proteins were pooled, concentrated, and TEV-cleaved by adding TEV protease at a ratio of 1:10 protease to protein and incubated overnight at 4 °C. TEV-cleaved proteins were loaded onto a Superdex™ 10/300 75 pg (Cytiva) SEC column, and eluted in 50 mM HEPES, pH 8.0, 150 mM NaCl, 10% glycerol, 2 mM DTT. Fractions containing pure protein were collected, pooled, concentrated to 30 mg/mL and stored at −80 °C.

1.21. NEMO Protein Expression

NEMO (IKBKG, residues 48-112) with an N-terminal 6xHis–yBBr–TEV tag was recombinantly expressed in *E. coli* BL21 CodonPlus RIPL cells (Agilent) from pET-derived expression vectors (Novagen). The cells were induced at OD600 = 0.6 with 1 mM IPTG for 4 h at 37 °C, then harvested and resuspended in 20 mM Tris-HCl pH 8.0, 150 mM NaCl, 1M urea, 1.0% Triton X-100. For purification, the pellet was lysed with a tip sonicator, and pelleted at 22,000 x g for 30 min at 4 °C. The pellets were washed three times and the inclusion body was dissolved in 20 mM Tris-HCl pH 8.0, 150 mM NaCl, and 8 M urea. The supernatant was purified with Ni-NTA resin (Qiagen), eluting with 20 mM Tris-HCl pH 8.0, 150 mM NaCl, 8 M urea, and 250 mM imidazole. Protein elutes were diluted dropwise diluted into 50 mM Tris-HCl pH 8.0, 200 mM NaCl, 100 mM

$MgCl_2$, 100 mM $CaCl_2$, 200 mM L-Arginine, 1 mM DTT, 0.5 mM reduced glutathione (GSH), 0.05 mM oxidized glutathione (GSSG), and 10% glycerol, and then dialyzed into buffer 20 mM Tris-HCl pH 8.0, 150 mM NaCl, 2 mM DTT, 0.5 mM GSH, 0.05 mM GSSG, and 10% glycerol. Proteins were biotinylated via the yBBr reaction according to standard procedures.[3] Biotinylated proteins were pooled, concentrated, and loaded onto a Superdex™ 10/300 200pg (Cytiva) SEC column and eluted in 50 mM Tris pH 7.5, 200 mM NaCl, 10% glycerol, and 1 mM DTT. Fractions containing pure protein were collected, pooled, concentrated to ~2.5 mg/mL and stored at −80 °C.

1.22. FKBP12 Protein Expression

FKBP12 (residues 2-108) with an N-terminal 6xHis–yBBr–TEV tag was recombinantly expressed in *E. coli* BL21 CodonPlus RIPL cells (Agilent) from pET-derived expression vectors (Novagen). The cells were induced at OD600 = 0.6 with 0.15 mM IPTG for 16 h at 16 °C, then harvested and resuspended in 10 mM Tris-HCl pH 8.8, 200 mM NaCl, 10% glycerol, and 1 mM phenylmethylsulfonyl fluoride (PMSF). For purification, the pellet was lysed with a tip sonicator and pelleted at 22,000 x g for 30 min at 4 °C. The supernatant was purified with Ni-NTA resin (Qiagen), eluted with 20 mM Tris-HCl pH 8.8, 200 mM NaCl, 10% glycerol, 50 mM and 250 mM imidazole, and biotinylated via the yBBr reaction according to standard procedures.[3] Biotinylated proteins were diluted into buffer 20 mM Tris-HCl pH 8.8, 10% glycerol, 1 mM DTT, and loaded onto a Q-HP (Cytiva) column. Proteins were eluted with a gradient from 50 mM to 1000 mM NaCl. Protein elutes were pooled, concentrated, and loaded onto a Superdex™ 10/300 75 pg (Cytiva) SEC column, and eluted in 20 mM Tris-HCl pH 7.4, 200 mM NaCl, 10% glycerol, and 1 mM DTT. Fractions containing pure protein were collected, pooled, concentrated to ~5.4 mg/mL and stored at −80°C.

1.23. BCL11a Protein Expression

BCL11a (740-835) with an N-terminal GST-3C-yBBR-TEV tag was recombinantly expressed in One Shot™ BL21 Star™ (DE3) Chemically Competent *E. coli* (ThermoFisher) from pET-derived expression vectors (ThermoFisher) in Luria broth supplemented with 0.1 mg/mL ampicillin and 100 µM zinc chloride. The cells were induced at OD600 = 0.2 with 0.2 mM isopropyl β-D-1-thiogalactopyranoside (IPTG) for 16 h at 18 °C, then harvested and resuspended in lysis buffer (50 mM HEPES, 500 mM NaCl, 1 mM DTT, 2 mM ATP, 10 mM $MgCl_2$, 1% NP40, 5% glycerol, Roche complete inhibitors, 100 µM $ZnCl_2$, pH 7.5). For purification, the pellet was lysed with a tip sonicator then pelleted at 20,000 x g for 30 min at 4 °C. Supernatant was collected and purified with GST resin (Cytiva), eluting with 10 mM GSH. Protein was cleaved by adding HRV 3C protease at a ratio of 1:10 protease to protein and incubated overnight at 25 °C. Cleaved protein was then concentrated and diluted into formulation buffer (50 mM HEPES, 200 mM NaCl, 5% glycerol, 1 mM DTT, pH 7.5) and labeled with biotin via yBBR according to established methods.[3] Labeled protein was injected onto a Superdex® 75 10/300 GL gel filtration column equilibrated with formulation buffer. Protein fractions were collected, concentrated, aliquoted and frozen.

1.24. YAP1-WW1-WW2 Protein Expression

YAP1-WW1-WW2 (residues 164-266) with an N-terminal 6xhis-SUMO-yBBR tag was recombinantly expressed in One Shot™ BL21 Star™ (DE3) Chemically Competent *E. coli* (ThermoFisher) from pET-derived expression vectors (ThermoFisher) in Luria broth supplemented with 0.1 mg/mL ampicillin. The cells were induced at OD600 = 0.5 with 0.5 mM isopropyl β-D-1-thiogalactopyranoside (IPTG) for 8 h at 25 °C then overnight at 15 °C, then harvested and resuspended in lysis buffer (50 mM HEPES, 300 mM NaCl, 2 mM ATP, 10 mM $MgCl_2$, 1% NP40, 5% glycerol, Roche complete inhibitors, pH 7.5). For purification, the pellet was lysed with a tip sonicator then pelleted at 20,000 x g for 30 min at 4 °C. Supernatant was collected and purified with cOmplete™ His-Tag Purification Columns (Roche), eluting with 300 mM imidazole. Protein was SUMO cleaved by adding SUMO protease at a ratio of 1:100 protease to protein and incubated overnight at 25 °C. Cleaved protein was then concentrated and diluted into formulation buffer (50 mM HEPES, 150 mM NaCl, 5% glycerol, pH 7.5) and labeled with biotin via yBBR according to established methods.[3] Labeled protein was injected onto a Superdex® 75 10/300 GL gel filtration column equilibrated with formulation buffer. Protein fractions were collected, concentrated, aliquoted and frozen.

1.25. NEMO_iZIP Protein Expression

NEMO_iZIP (IKBKG, residues 51-112 with both N- and C-terminal coiled-coil based on GCN4[4] with an N-terminal 6xHis–yBBr–TEV tag was recombinantly expressed and refolded identical to NEMO, with pure protein collected, pooled, concentrated to ~1.0 mg/mL and stored at −80°C. Peptide Clusters from Phage Display

1.26. MDM2 Peptide Binders from Phage Display

1.26.1. MDM2 Binder Sequences

Cluster	ID	Sequence	Measured K_D
MDM2.C1	H101‡	`Ac-dpahwycdfaaqvcnfs-NH2`	5.5* μM
MDM2.C2	H102‡	`Ac-dpawnhceyaafvcsev-NH2`	2.7 μM
MDM2.C3	H103‡	`Ac-dpawyecmeaammcqqv-NH2`	0.88 μM
MDM2.C4	H104	`Ac-dpalvecnlaadmchfy-NH2`	1.8 μM
MDM2.C5	H105	`Ac-dpaantciwaaiecsmy-NH2`	16* μM
MDM2.C6	H106	`Ac-dpawatcmdaalnclqm-NH2`	1.8 μM
MDM2.C7	H107	`Ac-dpasrncqwaahlceyw-NH2`	1.1 μM
MDM2.C8	H108	`Ac-dpalaqcrwaawrcdfe-NH2`	12* μM
MDM2.C9	H109	`Ac-dpaswqcvmaamdcvld-NH2`	2.4 μM

Listed sequences were cyclized between C7 and C14 with *N,N'*-(1,4-phenylene)bis(2-bromoacetamide). *Listed K_D values are beyond tested concentrations. ‡Sequences were selected for co-crystallization with MDM2.

1.26.2. MDM2 Binder SPR Validation

SPR experiments were performed on a Biacore 8K (Cytiva) instrument at 25 °C in 1x HBS-P+ buffer (Cytiva) with 1% DMSO. An SA Series S sensor chip was docked and pre-conditioned with three injections of 50 mM NaOH/1 M NaCl to remove unbound streptavidin from the surface. Biotinylated MDM2[25-109] was diluted to 2 μg/mL in running buffer and immobilized to channels 1 through 8 at 5 μL/min for 71 s for a final immobilization level of ~390 RU. Peptides were diluted

to 5 µM in running buffer then serially diluted 2-fold for a total of seven concentrations with one blank (7-point two-fold peptide dilution series with top concentration = 5 µM and bottom concentration = 78 nM). Compounds were injected over the immobilized and reference surfaces at 30 µL/min for 60 s and then allowed to dissociate for 180 s without surface regeneration (N = 1). Data was analyzed using Biacore Insight Evaluation software (Cytiva). Sensorgrams were double referenced, with most of them fitted to a 1:1 steady state affinity model, with a few fitted with both steady state affinity model and kinetics model.

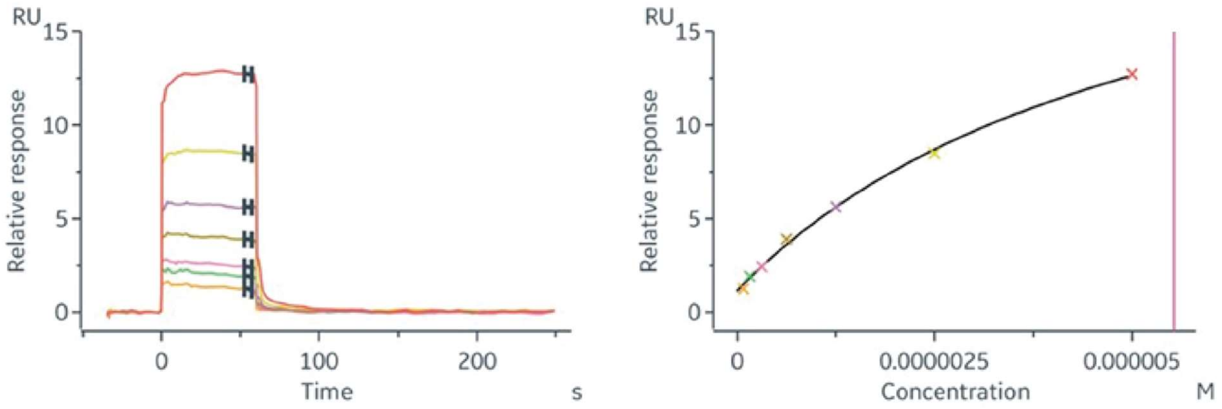

Supplementary Figure 6. D-H101 SPR to Recombinant MDM2. Sensorgrams and steady state affinity plots of D-H101 binding to recombinant MDM2 are shown.

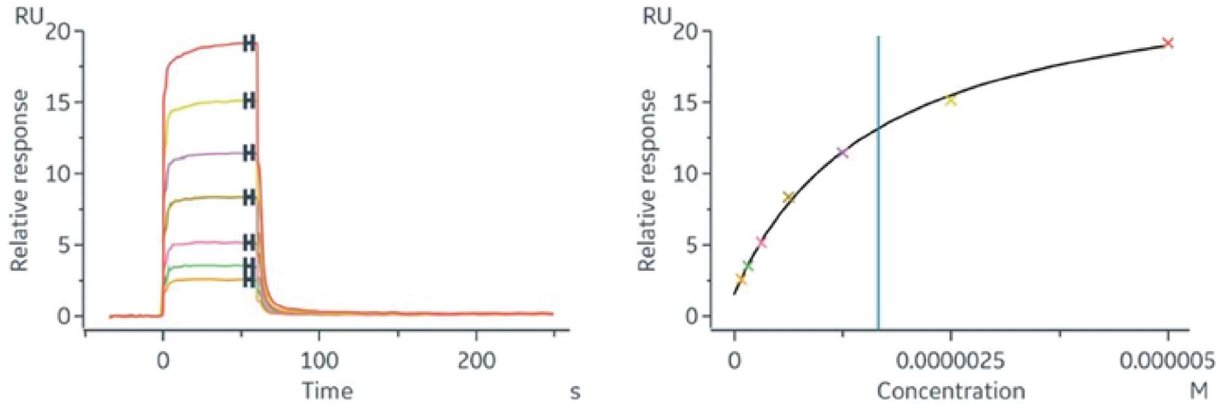

Supplementary Figure 7. D-H102 SPR to Recombinant MDM2. Sensorgrams and steady state affinity plots of D-H102 binding to recombinant MDM2 are shown.

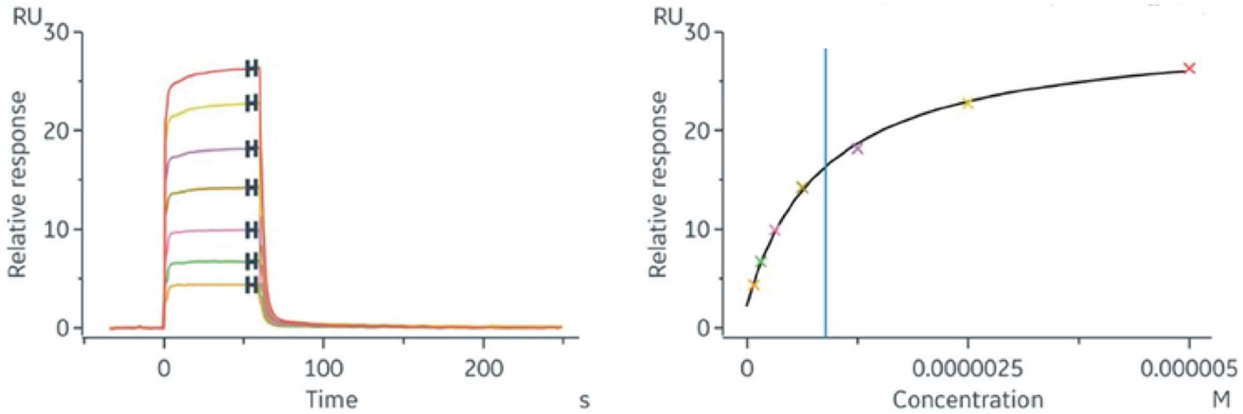

Supplementary Figure 8. D-H103 SPR to Recombinant MDM2. Sensorgrams and steady state affinity plots of D-H103 binding to recombinant MDM2 are shown.

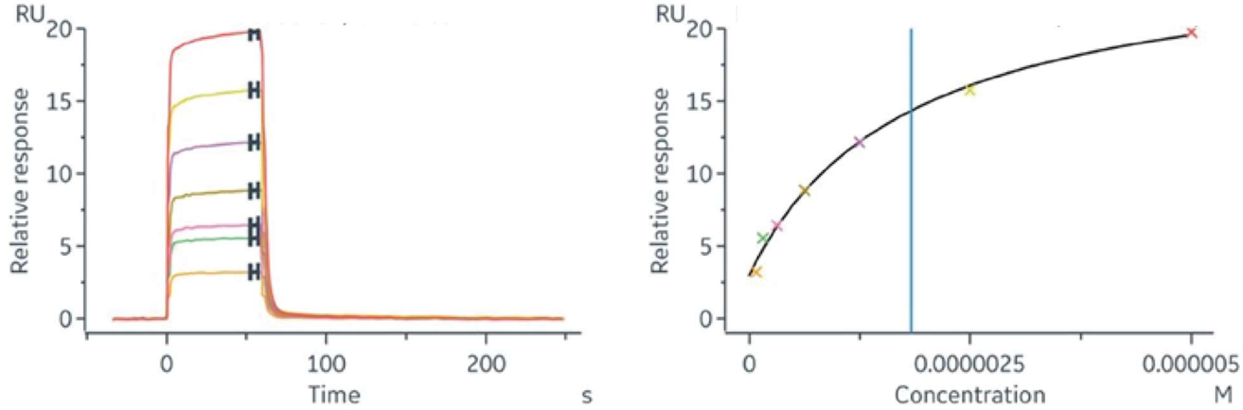

Supplementary Figure 9. D-H104 SPR to Recombinant MDM2. Sensorgrams and steady state affinity plots of D-H104 binding to recombinant MDM2 are shown.

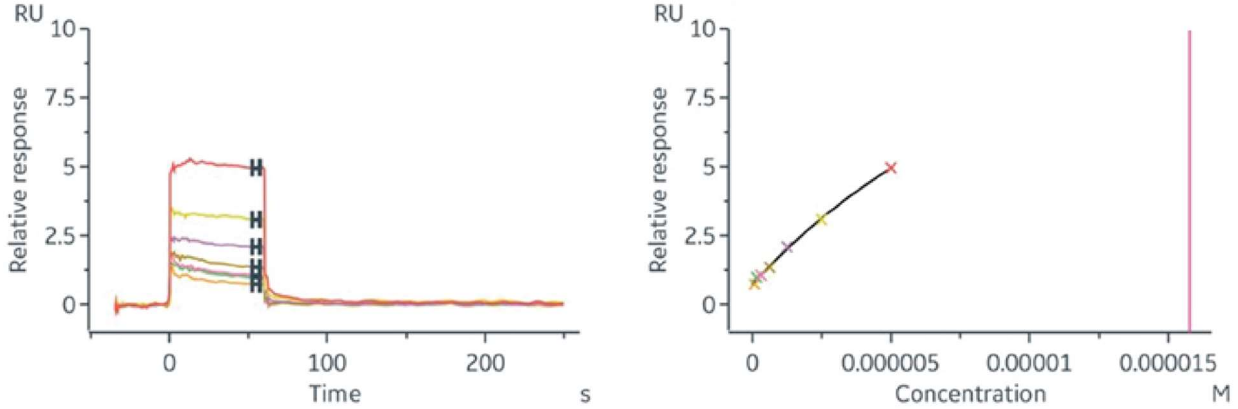

Supplementary Figure 10. D-H105 SPR to Recombinant MDM2. Sensorgrams and steady state affinity plots of D-H105 binding to recombinant MDM2 are shown.

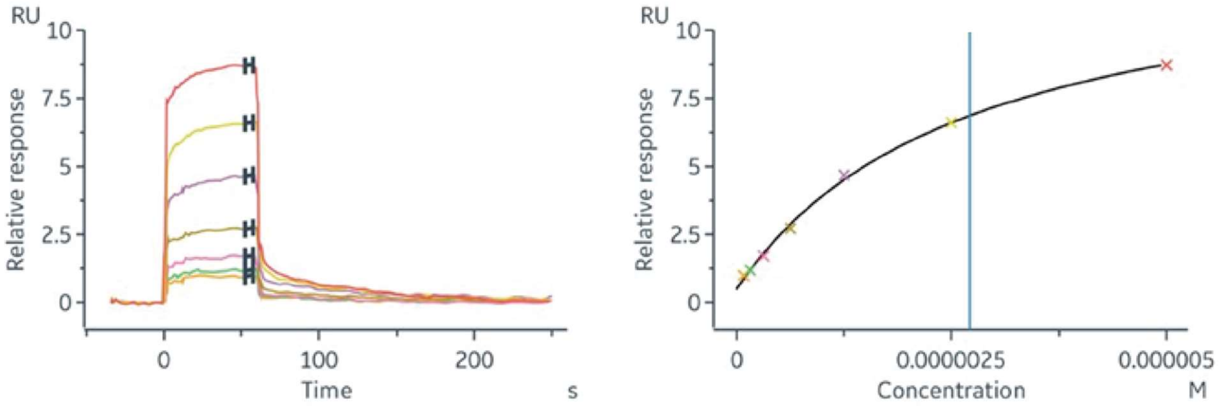

Supplementary Figure 11. D-H106 SPR to Recombinant MDM2. Sensorgrams and steady state affinity plots of D-H106 binding to recombinant MDM2 are shown.

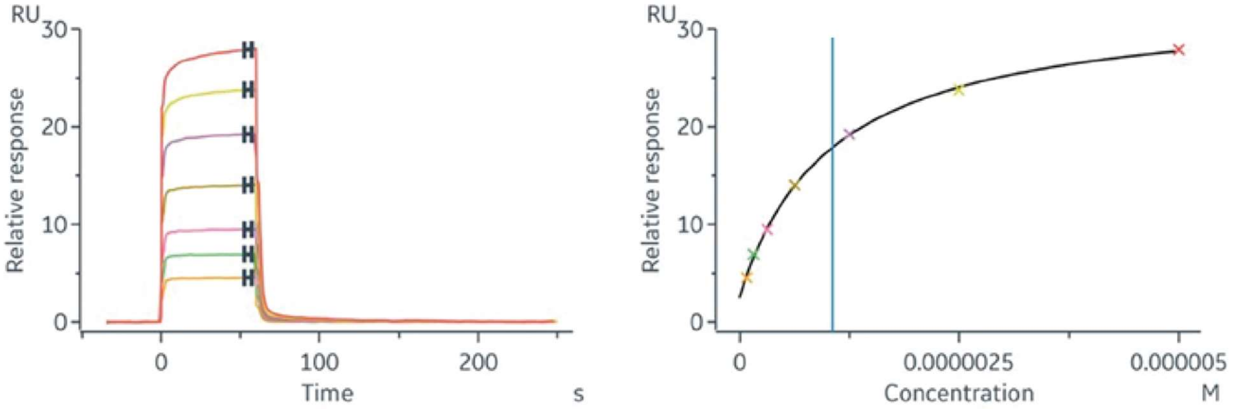

Supplementary Figure 12. D-H107 SPR to Recombinant MDM2. Sensorgrams and steady state affinity plots of D-H107 binding to recombinant MDM2 are shown.

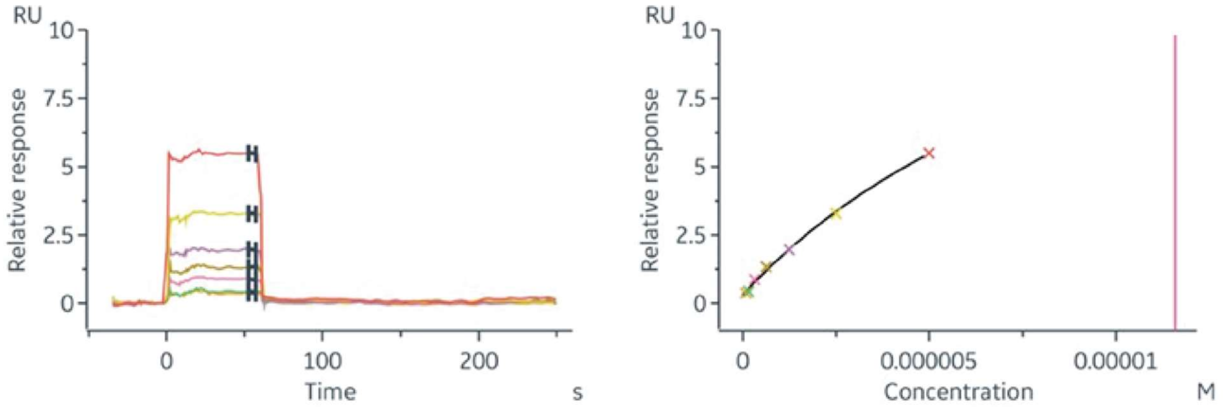

Supplementary Figure 13. D-H108 SPR to Recombinant MDM2. Sensorgrams and steady state affinity plots of D-H108 binding to recombinant MDM2 are shown.

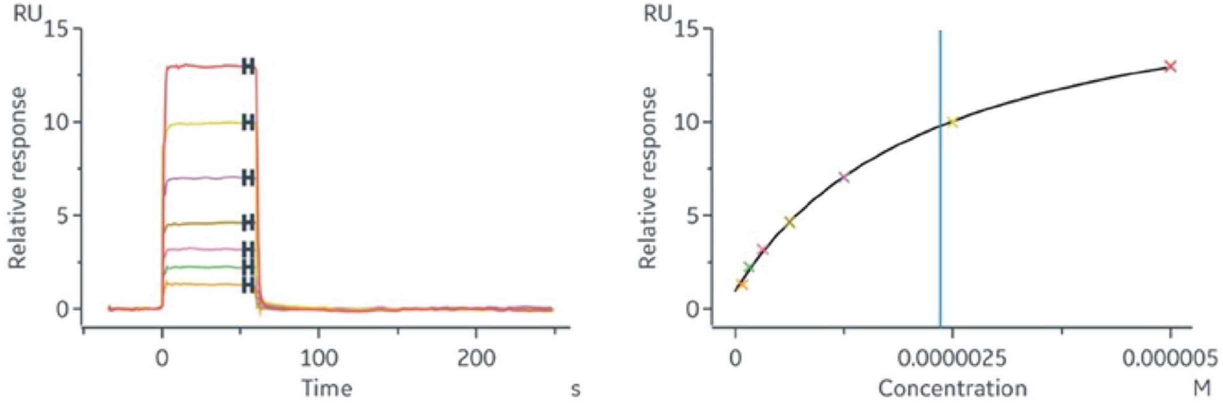

Supplementary Figure 14. D-H109 SPR to Recombinant MDM2. Sensorgrams and steady state affinity plots of D-H109 binding to recombinant MDM2 are shown.

1.27. CHIP Peptide Binders from Phage Display

1.27.1. CHIP Binder Sequences

Cluster	ID	Sequence	Measured IC$_{50}$
CHIP.C1	H201[‡]	`Ac-pwedcawfawacyni-NH`$_2$	470 nM
CHIP.C3	H202[‡]	`Ac-pwweclsqaddcdfr-NH`$_2$	1230 M
CHIP.C2	H203[‡]	`Ac-phemcywadaycrys-NH`$_2$	550 nM
CHIP.C2	H204[‡]	`Ac-pldlcywaslhcivs-NH`$_2$	770 nM

Listed sequences were cyclized between C5 and C12 with *N,N'*-(1,4-phenylene)bis(2-bromoacetamide). [‡]Sequences were selected for co-crystallization with CHIP.

1.27.2. CHIP Binder FP Validation

For the competition FP of CHIP, Helicons at 10 mM in DMSO were serially diluted 1:3 in DMSO for a total of 11 concentrations using a Mosquito LV (SPT Labtech), then diluted 1000-fold in buffer (1 x HBS-P+, Cytiva) in duplicate by the Mosquito LV (SPT Labtech) into a black polystyrene 384-well plate (Corning) (11-point three-fold peptide dilution series with top concentration = 10 µM). The assay was performed with 400 nM CHIP23-303 recombinant protein as target, and 20 nM CHIP-binding peptide as probe (5FAM-bAla-SSGPTIEEVD, derived from HSP70). The plate was incubated and protected from light for 1 hour at room temperature prior to reading. Reads were performed on a CLARIOstar plate reader (BMG Labtech) with excitation at 485 nm, emission at 525 nm, and cutoff at 504 nm. Data were fitted to a 1:1 binding model with Hill slope using an in-house script.

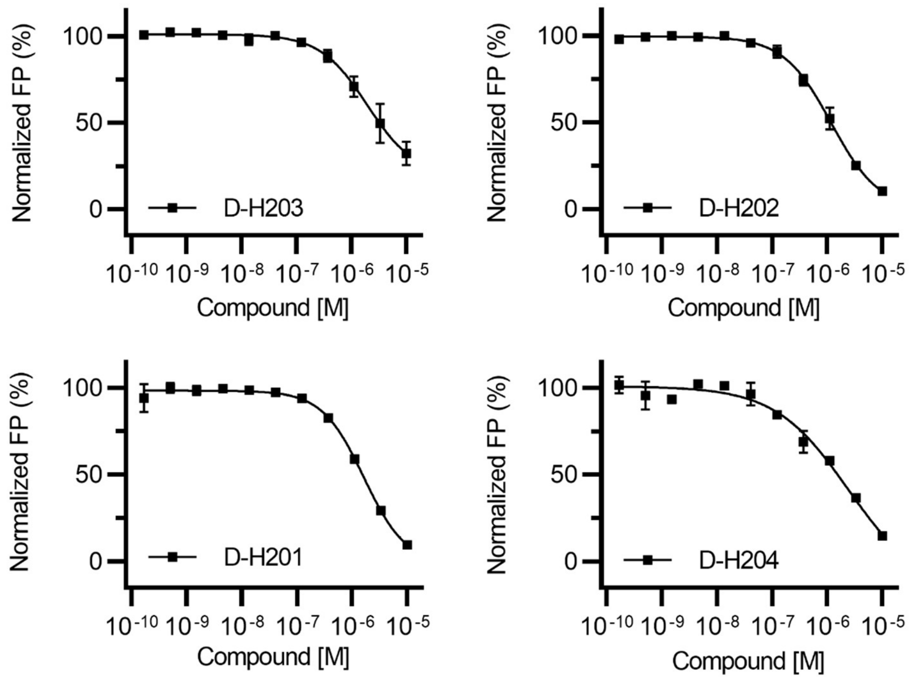

Supplementary Figure 15. FP validation of D-Helicons to CHIP. Normalized FP signal of synthetic D-helicon binders to recombinant CHIP are shown. Experiments were performed with n = 2 technical replicates.

1.28. List of Protein Sequences

Protein	Uniprot ID	Start	End	Length	Modifications
MDM2	Q00987	26	109	84	N-Term PEG_{12}Biotin, C-Term carboxamide
TLVRPKPLLL KLLKSVGAQK DTYTMKEVLF YLGQYIMTKR LYDEKQQHIV YCSNDLLGDL FGVPSFSVKE HRKIYTMIYR NLVV					
ERG	P11308	108	201	94	N-Term PEG_{12}Biotin, C-Term carboxamide
MEEKHMPPPN MTTNERRVIV PADPTLWSTD HVRQWLEWAV KEYGLPDVNI LLFQNIDGKE LCKMTKDDFQ RLTPSYNADI LLSHLHYLRE TPLP					
Barnase	P00648	48	157	110	N-Term PEG_{12}Biotin, C-Term carboxamide
AQVINTFDGV ADYLQTYHKL PDNYITKSEA QALGWVASKG NLADVAPGKS IGGDIFSNRE GKLPGKSGRT WREADINYTS GFRNSDRILY SSDWLIYKTT DHYQTFTKIR					
IRAK2	O43187	2	112	111	N-Term PEG_{12}Biotin, C-Term carboxamide
ACYIYQLPSW VLDDLCRNMD ALSEWDWMEF ASYVITDLTQ LRKIKSMERV QGVSITRELL WWWGMRQATV QQLVDLLCRL ELYRAAQIIL NWKPAPEIRC PIPAFPDSVK P					
CHIP	Q9UNE7	23	153	132	N-Term PEG_{12}Biotin, C-Term carboxamide
SPSAQELKEQ GNRLFVGRKY PEAAACYGRA ITRNPLVAVY YTNRALCYLK MQQHEQALAD CRRALELDGQ SVKAHFFLGQ CQLEMESYDE AIANLQRAYS LAKEQRLNFG DDIPSALRIA KKKRWNSIEE RR					
NEMO	Q9Y6K9	44	112	70	N-Term PEG_{12}Biotin, C-Term carboxamide, Nle in place of Met
EQGAPETLQR CLEENQELRD AIRQSNQILR ERCEELLHFQ ASQREEKEFL MCKFQEARKL VERLGLEKLE					
FKBP12	P62942	2	108	107	N-Term PEG_{12}Biotin, C-Term carboxamide
GVQVETISPG DGRTFPKRGQ TCVVHYTGML EDGKKFDSSR DRNKPFKFML GKQEVIRGWE EGVAQMSVGQ RAKLTISPDY AYGATGHPGI IPPHATLVFD VELLKLE					
BCL11a	Q9H165	740	835	96	N-Term PEG_{12}Biotin, C-Term carboxamide
RSDTCEYCGK VFKNCSNLTV HRRSHTGERP YKCELCNYAC AQSSKLTRHM KTHGQVGKDV YKCEICKMPF SVYSTLEKHM KKWHSDRVLN NDIKTE					
Yap1	P46937	163	276	114	N-Term PEG_{12}Biotin, C-Term carboxamide, Nle in place of Met
SSFEIPDDVP LPAGWEMAKT SSGQRYFLNH IDQTTTWQDP RKAMLSQMNV TAPTSPPVQQ NMMNSASGPL PDGWEQAMTQ DGEIYYINHK NKTTSWLDPR LDPRFAMNQR ISQS					
Nemo_iZip	*6MI4	4	124	121	N-Term PEG_{12}Biotin, C-Term carboxamide, Nle in place of Met
SVKELEDKNE ELLSEIAHLK NEVARLKKLL QRCLEENQEL RDAIRQSNQI LRERCEELLH FQASQREEKE FLMCKFQEAR KLVERLGLEK LELEDKNEEL LSEIAHLKNE VARLKKLVGE R					
Max	P61244	23	102	83	N-Term PEG_{12}Biotin, C-Term +GGC + carboxamide, Nle in place of Met
DKRAHHNALE RKRRDHIKDS FHSLRDSVPS LQGEKASRAQ ILDKATEYIQ YMRRKNHTHQ QDIDDLKRQN ALLEQQVRAL GGC					
Myc	P01106	353	427	85	N-Term PEG_{12}Biotin, C-Term +GGC + carboxamide
NVKRRTHNVL ERQRRNELKR SFFALRDQIP ELENNEKAPK VVILKKATAY ILSVQAEEQK LISEEDLLRK RREQLKHKLE QLGGC					
Max-nb	P61244	23	102	83	C-Term +GGC + carboxamide, Nle in place of Met
DKRAHHNALE RKRRDHIKDS FHSLRDSVPS LQGEKASRAQ ILDKATEYIQ YMRRKNHTHQ QDIDDLKRQN ALLEQQVRAL GGC					

*PDB ID referenced in place of Uniprot ID.

2. Supplementary Results

2.1. Crude HPLC Data for L-Protein Targets

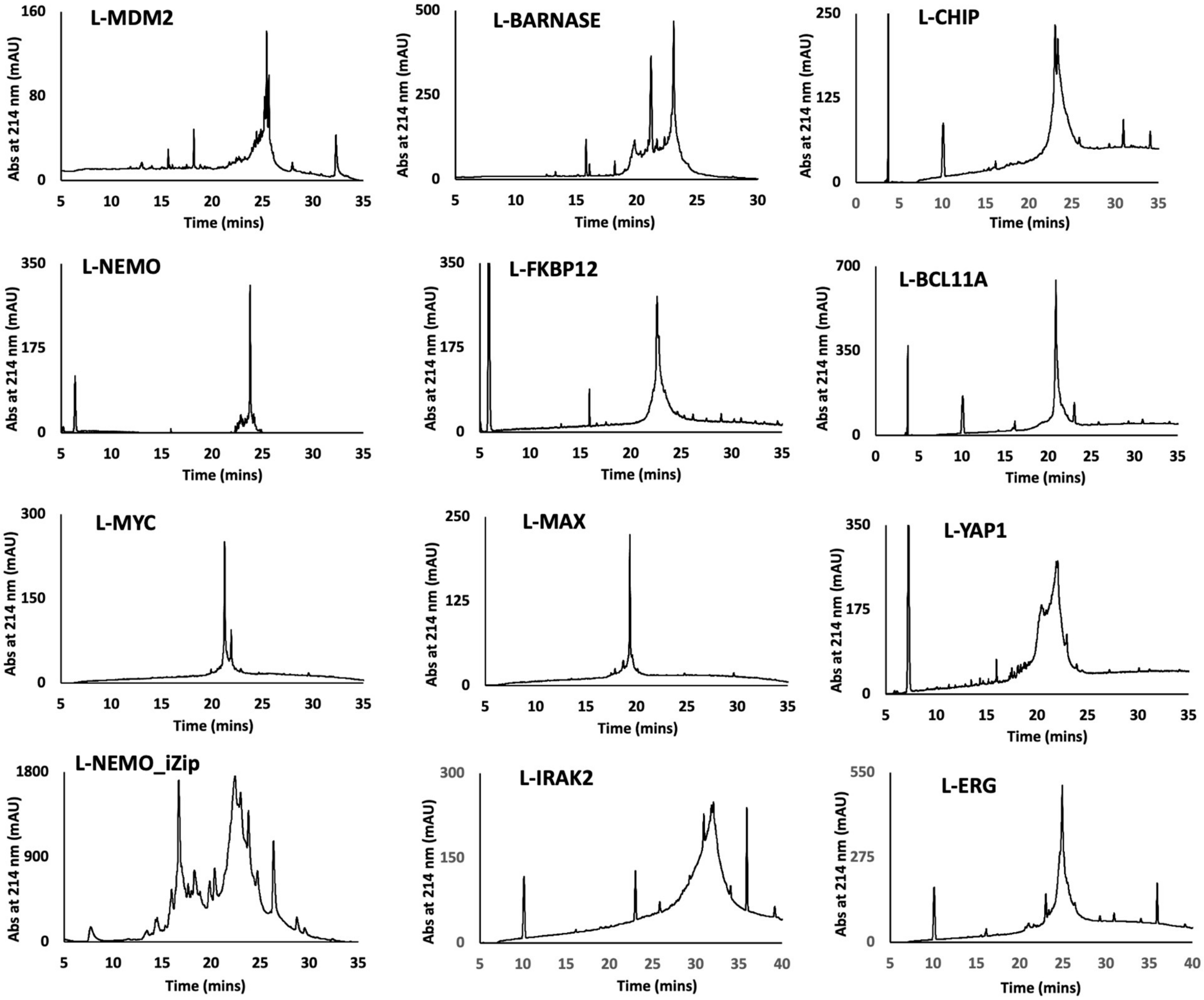

2.2. Crude HPLC Data for D-Protein Targets

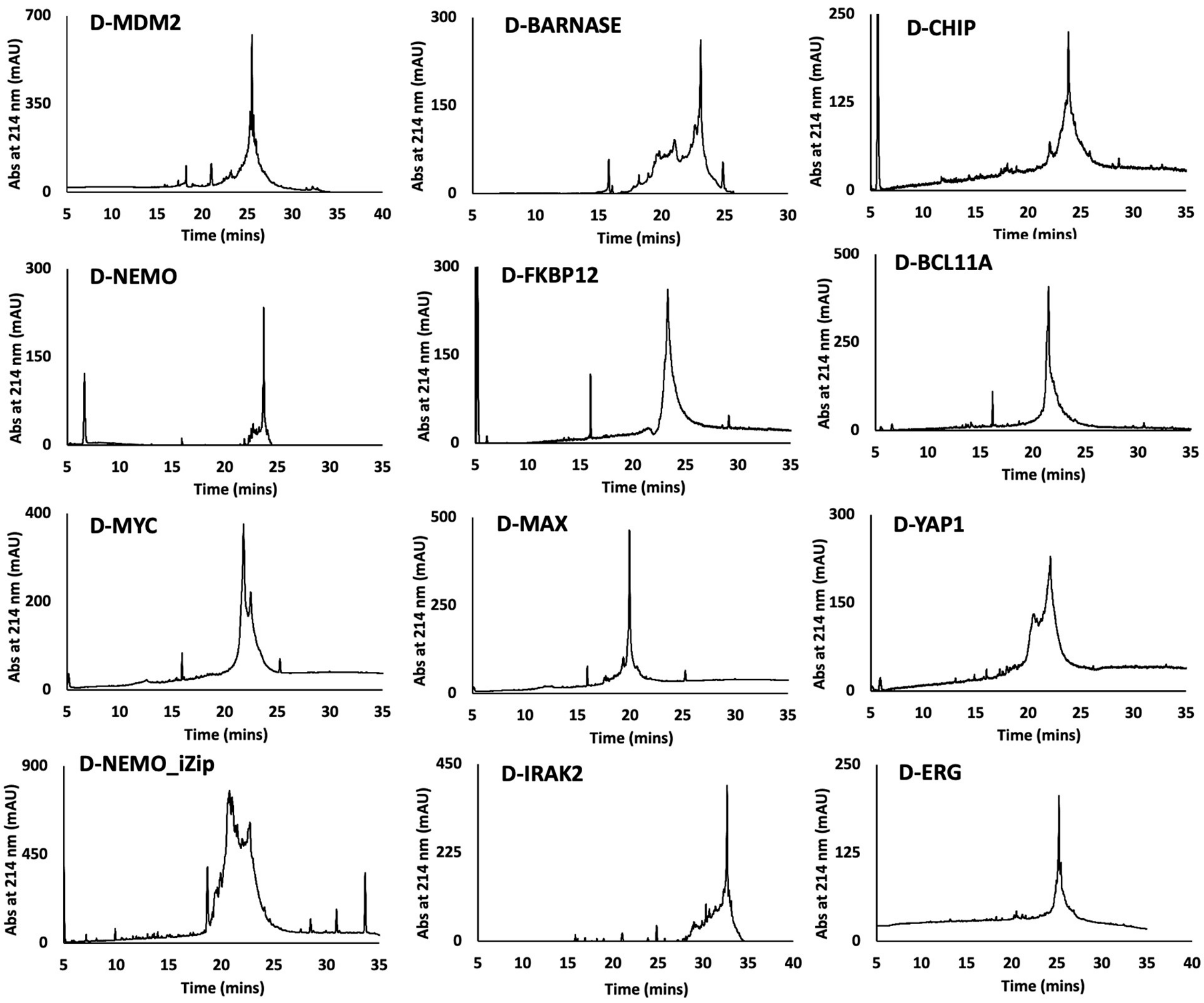

2.3. MDM2 from AFPS

Protein	Uniprot ID	Start	End	Length	Modifications
MDM2	Q00987	26	109	84	N-Term PEG_{12}Biotin, C-Term carboxamide
TLVRPKPLLL KLLKSVGAQK DTYTMKEVLF YLGQYIMTKR LYDEKQQHIV YCSNDLLGDL FGVPSFSVKE HRKIYTMIYR NLVV					

2.3.1. L-MDM2 AFPS Results

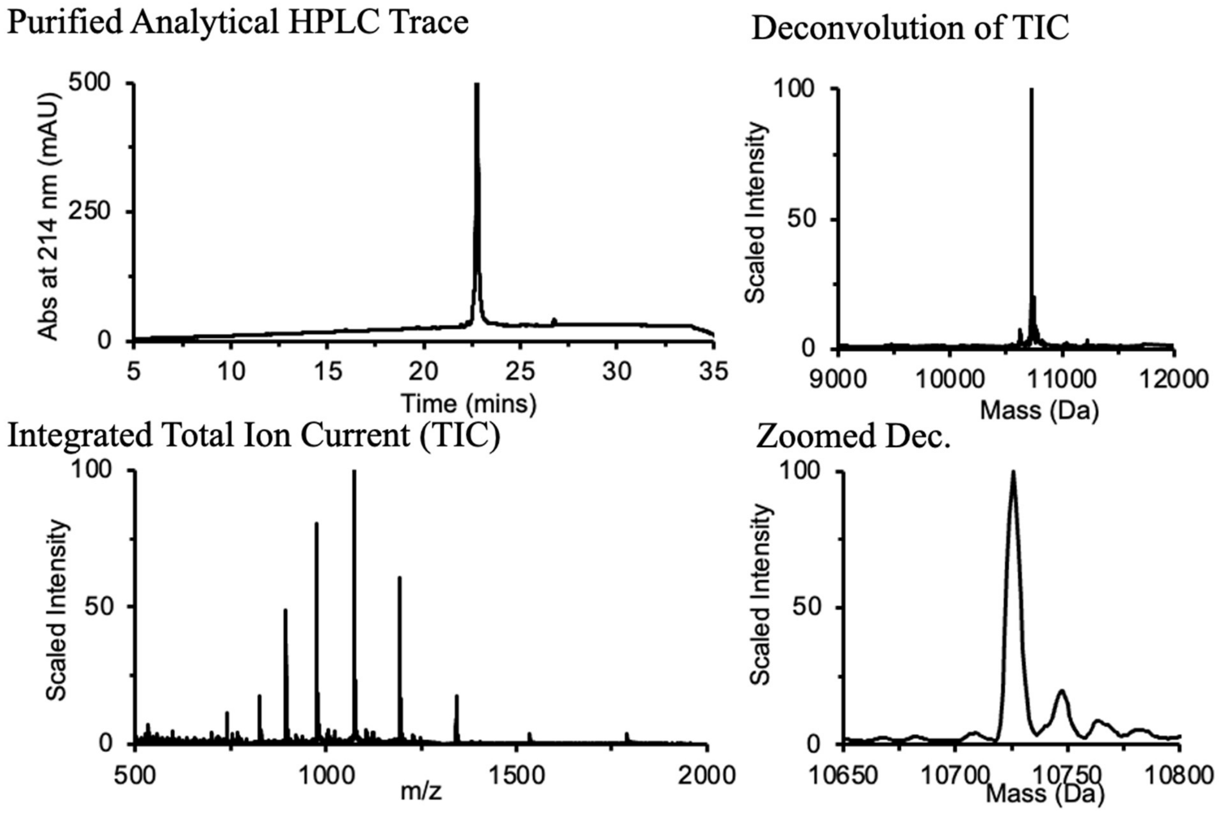

Starting Resin	Crude Resin	Cleaved Resin	Isolated Mass	Theoretical Mass	Purified Mass	Isolated after Purification	Extrapolated Yield
130 mg	428.8 mg	213.7 mg	65.4 mg	137.0 mg	35.0 mg	1.3 mg	4.9 mg
0.18 mmol / g (23.4 μmol)	50% of total resin		48% Yield		54% of Isolated Peptide	4% Purification Yield	1.8% Overall Yield

Cleavage Method: Method 1.1.1 - Reagent K
Purification Method: Method 1.4.1 - 2 Stage Semi-Prep
Purified HPLC Method: Method 1.2.1 - 5% to 65% in 30 min on Agilent 1200
Purified LCMS Method: Method 1.3.1 - 6545 1-91 in 8 min
Calculated Mass: 10727 Da
Observed Mass: 10727 Da

2.3.2. D-MDM2 AFPS Results

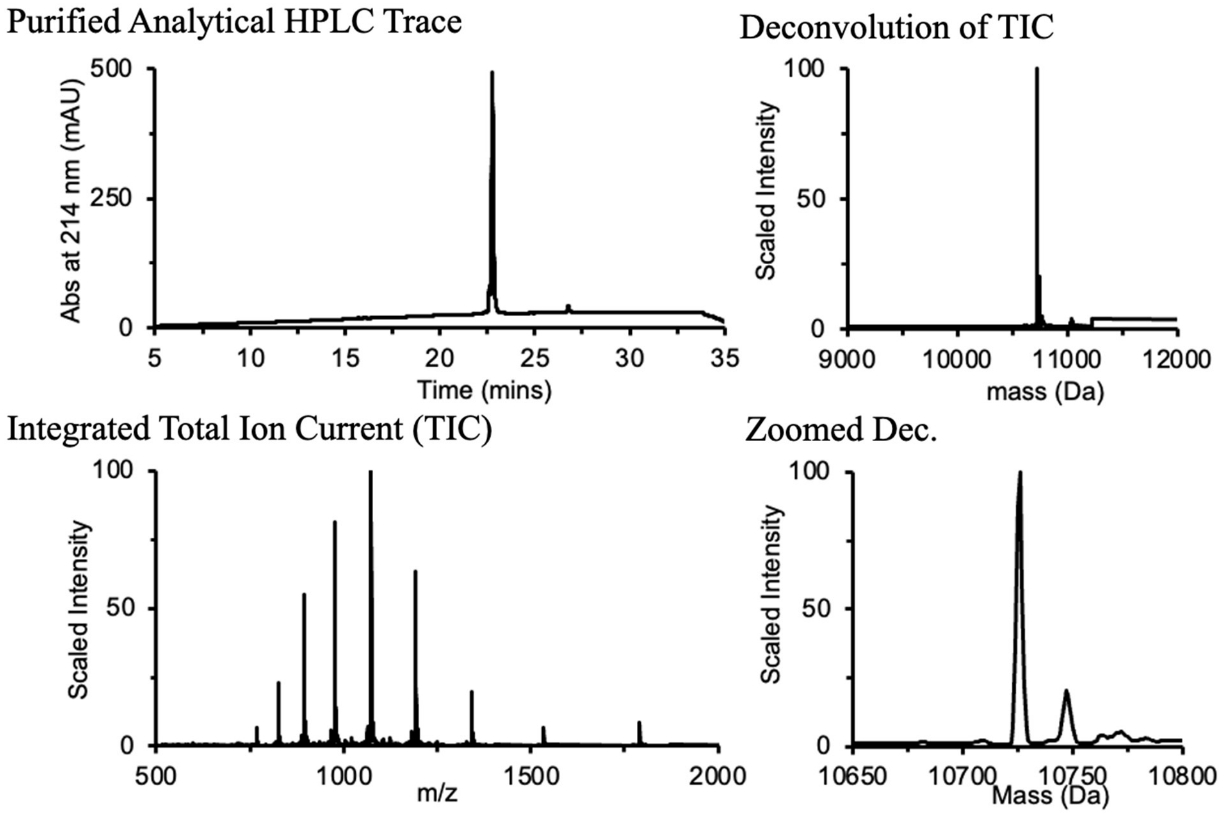

Starting Resin	Crude Resin	Cleaved Resin	Isolated Mass	Theoretical Mass	Purified Mass	Isolated after Purification	Extrapolated Yield
130 mg	402.3 mg	202.7 mg	53.0 mg	138.5 mg	26.5 mg	2.5 mg	9.9 mg
0.18 mmol / g (23.4 μmol)	50% of total resin		38% Yield		50% of Isolated Peptide	9% Purification Yield	3.6% Overall Yield

<u>*Cleavage Method*</u>: Method 1.1.1 - Reagent K
<u>*Purification Method*</u>: Method 1.4.1 - 2 Stage Semi-Prep
<u>*Purified HPLC Method*</u>: Method 1.2.2 - 5% to 65% in 15 min on Agilent 1200
<u>*Purified LCMS Method*</u>: Method 1.3.1 - 6545 1-91 in 8 min
<u>*Calculated Mass*</u>: 10727 Da
<u>*Observed Mass*</u>: 10727 Da

2.4. ERG from AFPS

Protein	Uniprot ID	Start	End	Length	Modifications
ERG	P11308	108	201	94	N-Term PEG_{12}Biotin, C-Term carboxamide
MEEKHMPPPN MTTNERRVIV PADPTLWSTD HVRQWLEWAV KEYGLPDVNI LLFQNIDGKE LCKMTKDDFQ RLTPSYNADI LLSHLHYLRE TPLP					

2.4.1. L-ERG AFPS Results

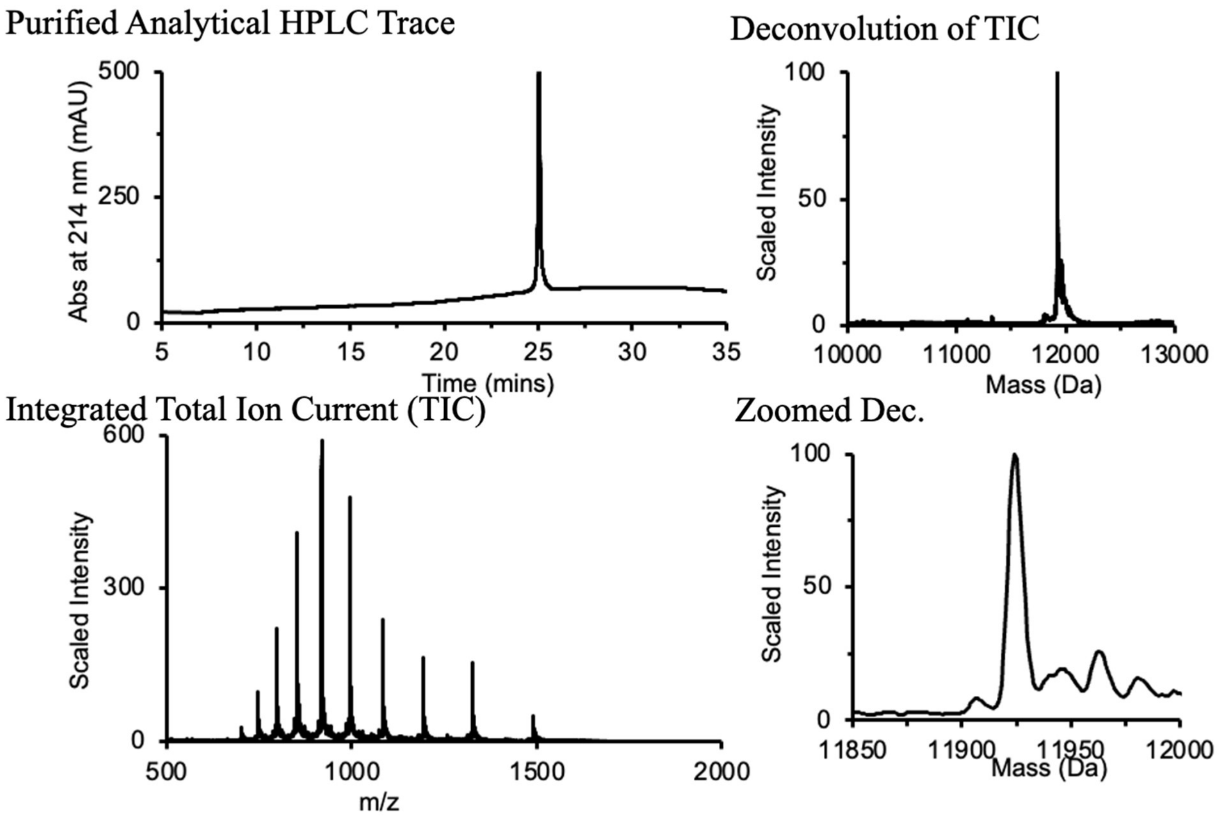

Starting Resin	Crude Resin	Cleaved Resin	Isolated Mass	Theoretical Mass	Purified Mass	Isolated after Purification	Extrapolated Yield
130 mg	377.4 mg	188.7 mg	60.2 mg	149.4 mg	60.2 mg	6.3 mg	12.6 mg
0.18 mmol / g (23.4 μmol)	50% of total resin		40% Yield		100% of Isolated Peptide	10% Purification Yield	4.2% Overall Yield

<u>Cleavage Method</u>: Method 1.1.1 - Reagent K
<u>Purification Method</u>: Method 1.4.1 - 2 Stage Semi-Prep
<u>Purified HPLC Method</u>: Method 1.2.1 - 5% to 65% in 30 min on Agilent 1200
<u>Purified LCMS Method</u>: Method 1.3.1 - 6545 1-91 in 8 min
<u>Calculated Mass</u>: 11926 Da
<u>Observed Mass</u>: 11926 Da

2.4.2. D-ERG AFPS Results

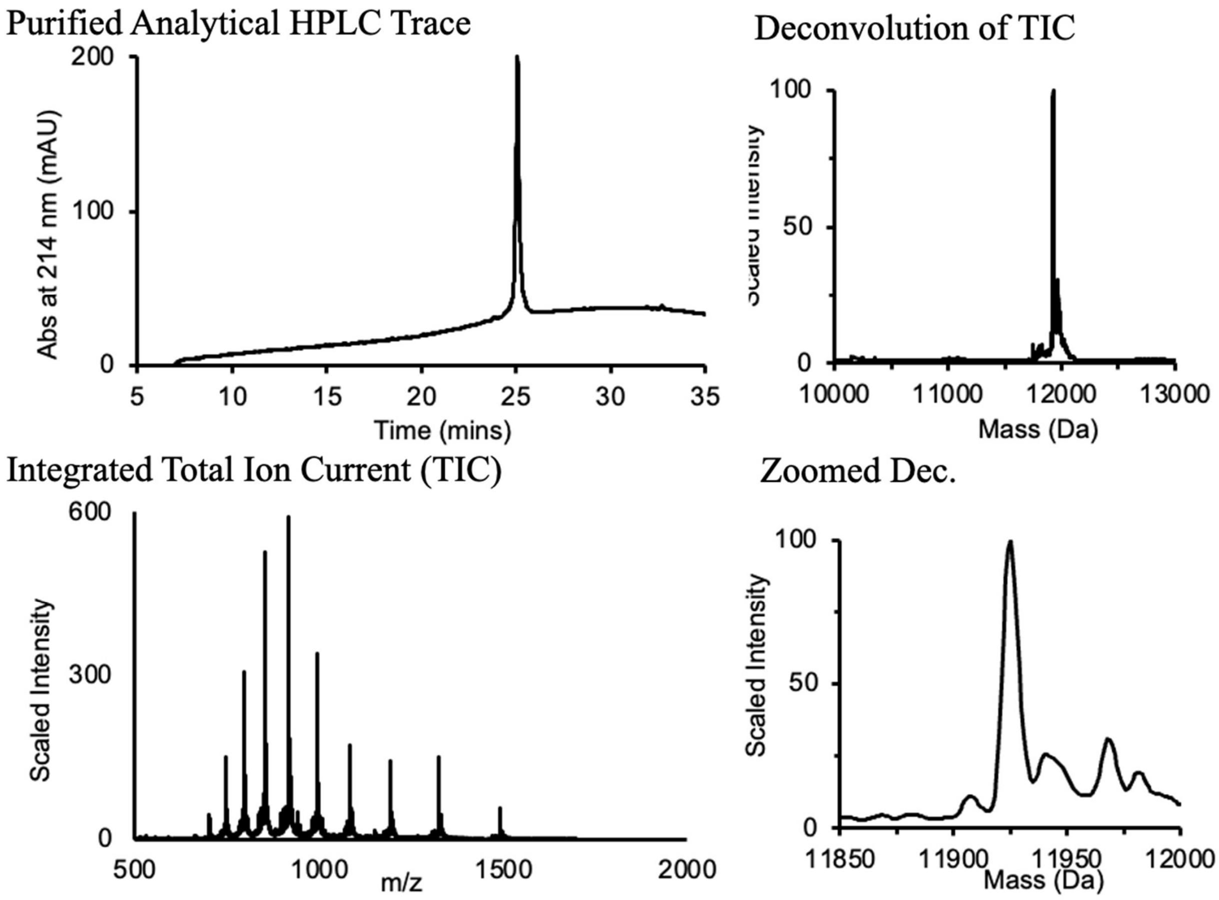

Starting Resin	Crude Resin	Cleaved Resin	Isolated Mass	Theoretical Mass	Purified Mass	Isolated after Purification	Extrapolated Yield
130 mg	384.8 mg	192.8 mg	73.2 mg	149.7 mg	73.2 mg	6.9 mg	13.9 mg
0.18 mmol / g (23.4 μmol)	50% of total resin		49% Yield		100% of Isolated Peptide	9% Purification Yield	4.6% Overall Yield

Cleavage Method: Method 1.1.1 - Reagent K
Purification Method: Method 1.4.1 - 2 Stage Semi-Prep
Purified HPLC Method: Method 1.2.1 - 5% to 65% in 30 min on Agilent 1200
Purified LCMS Method: Method 1.3.1 - 6545 1-91 in 8 min
Calculated Mass: 11926 Da
Observed Mass: 11926 Da

2.5. Barnase from AFPS

Protein	Uniprot ID	Start	End	Length	Modifications
Barnase	P00648	48	157	110	N-Term PEG_{12}Biotin, C-Term carboxamide
AQVINTFDGV ADYLQTYHKL PDNYITKSEA QALGWVASKG NLADVAPGKS IGGDIFSNRE GKLPGKSGRT WREADINYTS GFRNSDRILY SSDWLIYKTT DHYQTFTKIR					

2.5.1. L-Barnase AFPS Results

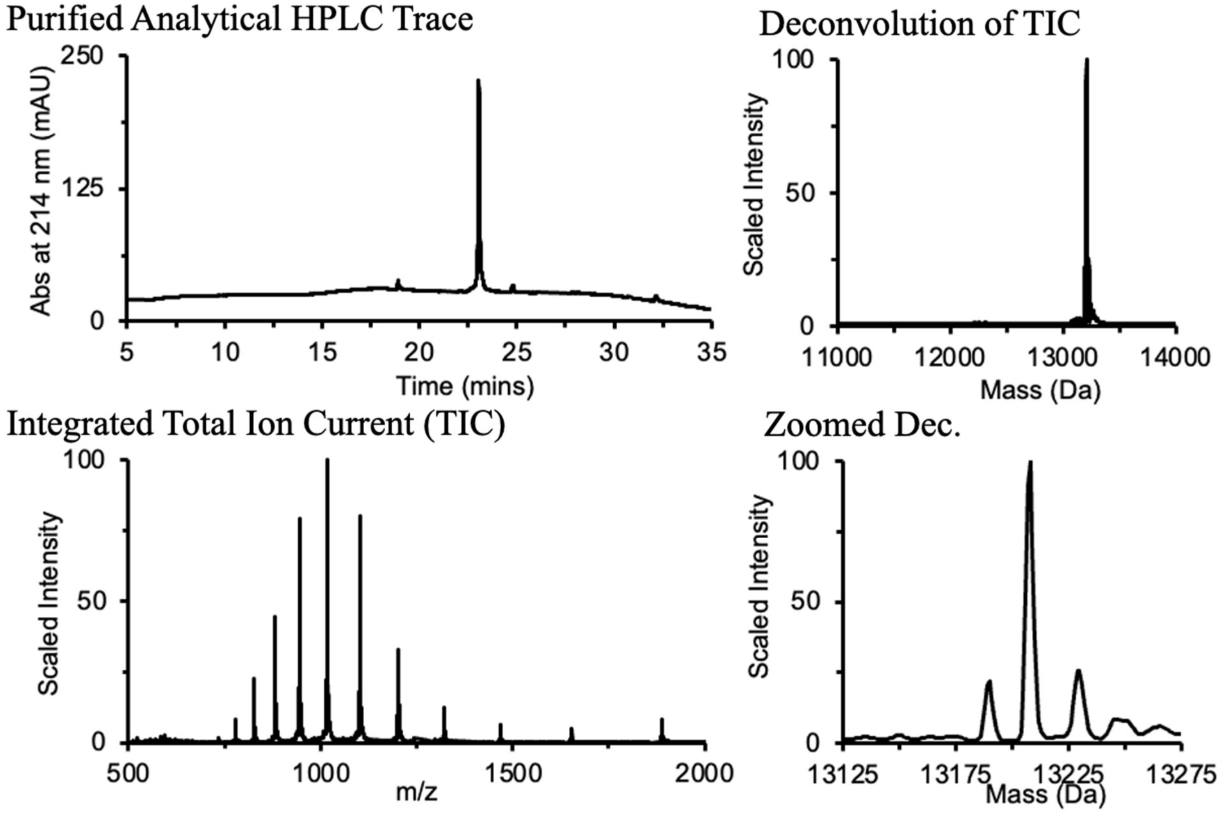

Starting Resin	Crude Resin	Cleaved Resin	Isolated Mass	Theoretical Mass	Purified Mass	Isolated after Purification	Extrapolated Yield
130 mg	407.2 mg	196.4 mg	31.1 mg	160.2 mg	31.1 mg	1.6 mg	3.3 mg
0.18 mmol / g (23.4 μmol)	48% of total resin		19% Yield		100% of Isolated Peptide	5% Purification Yield	1.0% Overall Yield

<u>*Cleavage Method*</u>: Method 1.1.1 - Reagent K
<u>*Purification Method*</u>: Method 1.4.1 - 2 Stage Semi-Prep
<u>*Purified HPLC Method*</u>: Method 1.2.1 - 5% to 65% in 30 min on Agilent 1200
<u>*Purified LCMS Method*</u>: Method 1.3.1 - 6545 1-91 in 8 min
<u>*Calculated Mass*</u>: 13209 Da
<u>*Observed Mass*</u>: 13209 Da

2.5.2. D-Barnase AFPS Results

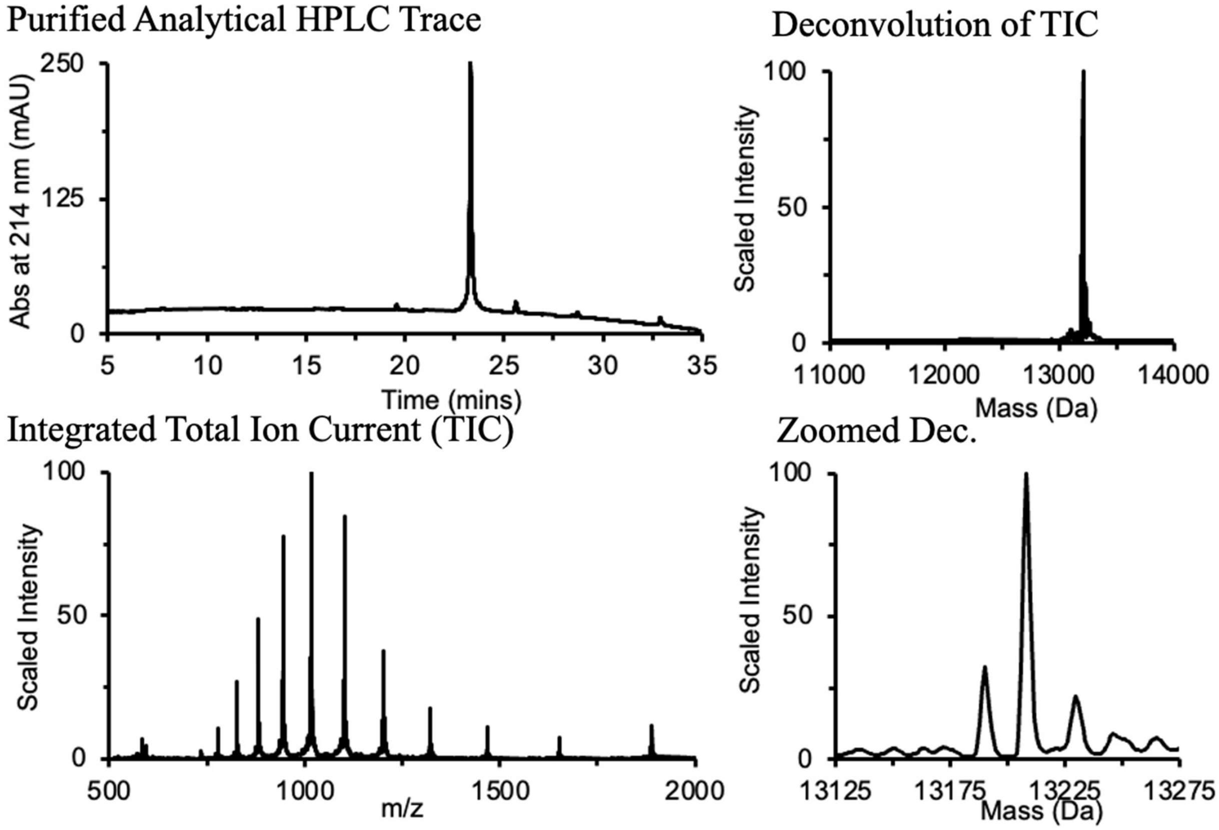

Starting Resin	Crude Resin	Cleaved Resin	Isolated Mass	Theoretical Mass	Purified Mass	Isolated after Purification	Extrapolated Yield
130 mg	384.4 mg	190.9 mg	32.0 mg	165.0 mg	32.0 mg	2.3 mg	4.6 mg
0.18 mmol / g (23.4 μmol)	50% of total resin		19% Yield		100% of Isolated Peptide	7% Purification Yield	1.4% Overall Yield

Cleavage Method: Method 1.1.1 - Reagent K
Purification Method: Method 1.4.1 - 2 Stage Semi-Prep
Purified HPLC Method: Method 1.2.1 - 5% to 65% in 30 min on Agilent 1200
Purified LCMS Method: Method 1.3.1 - 6545 1-91 in 8 min
Calculated Mass: 13209 Da
Observed Mass: 13209 Da

2.6. IRAK2 from AFPS

Protein	Uniprot ID	Start	End	Length	Modifications
IRAK2	O43187	2	112	111	N-Term PEG_{12}Biotin, C-Term carboxamide
ACYIYQLPSW VLDDLCRNMD ALSEWDWMEF ASYVITDLTQ LRKIKSMERV QGVSITRELL WWWGMRQATV QQLVDLLCRL ELYRAAQIIL NWKPAPEIRC PIPAFPDSVK P					

2.6.1. L-IRAK2 AFPS Results

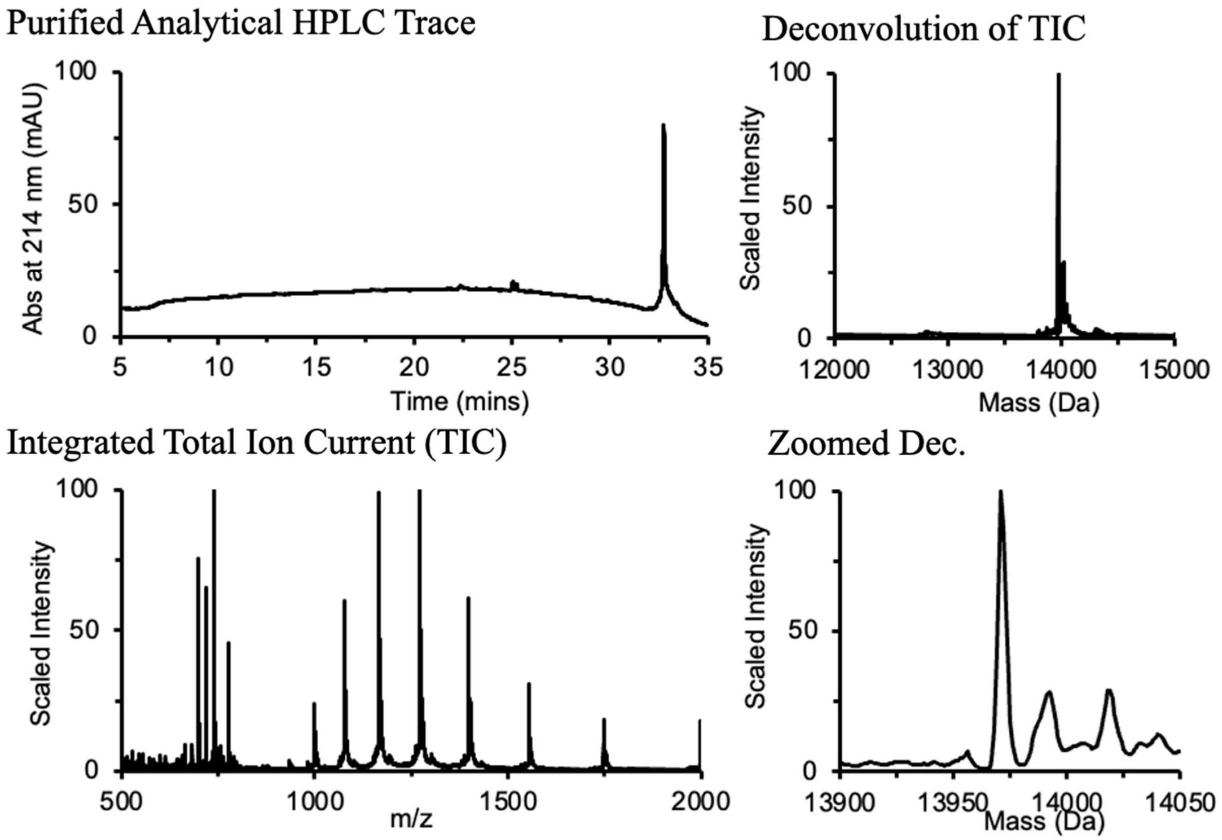

Starting Resin	Crude Resin	Cleaved Resin	Isolated Mass	Theoretical Mass	Purified Mass	Isolated after Purification	Extrapolated Yield
130 mg	402.0 mg	201.0 mg	22.4 mg	169.5 mg	22.4 mg	2.1 mg	4.1 mg
0.18 mmol / g (23.4 μmol)	50% of total resin		26% Yield		100% of Isolated Peptide	9% Purification Yield	1.2% Overall Yield

<u>*Cleavage Method*</u>: Method 1.1.1 - Reagent K
<u>*Purification Method*</u>: Method 1.4.1 - 2 Stage Semi-Prep
<u>*Purified HPLC Method*</u>: Method 1.2.1 - 5% to 65% in 30 min on Agilent 1200
<u>*Purified LCMS Method*</u>: Method 1.3.1 - 6545 1-91 in 8 min
<u>*Calculated Mass*</u>: 13972 Da
<u>*Observed Mass*</u>: 13972 Da

2.6.2. D-IRAK2 AFPS Results

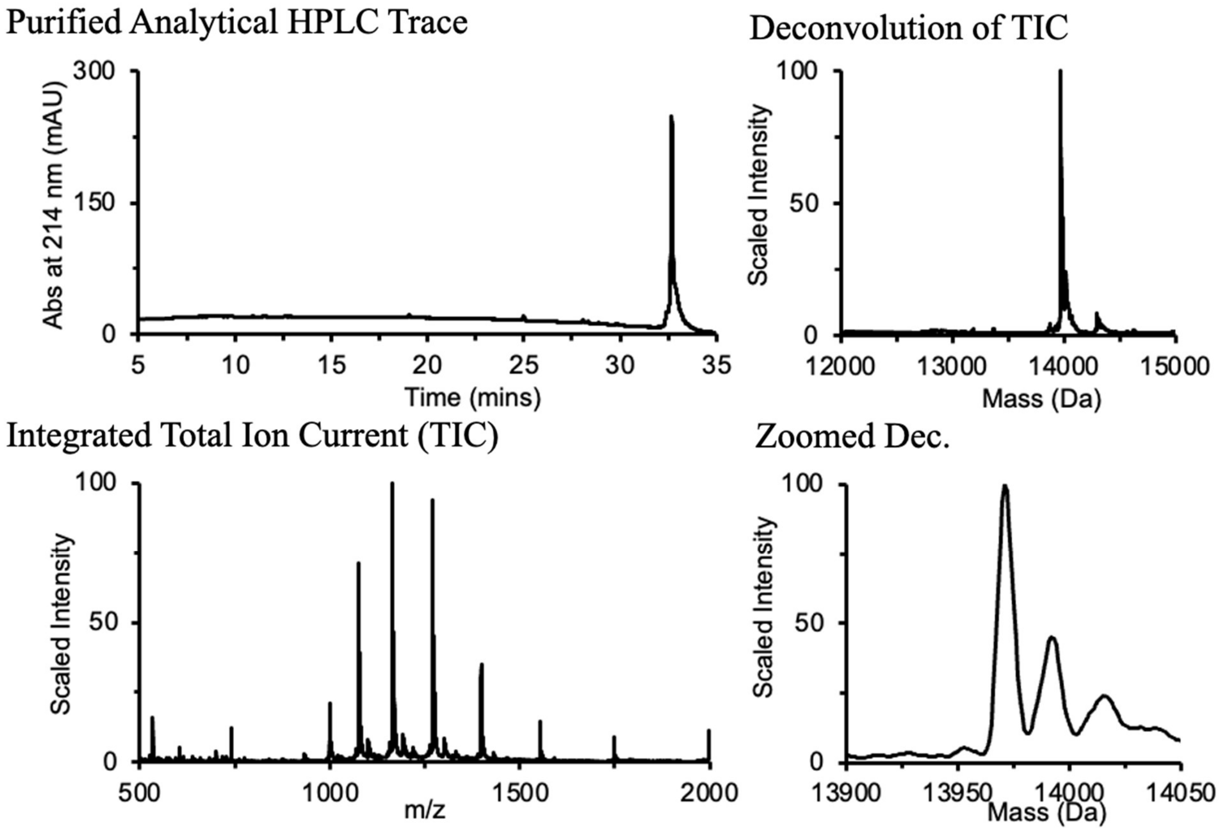

Starting Resin	Crude Resin	Cleaved Resin	Isolated Mass	Theoretical Mass	Purified Mass	Isolated after Purification	Extrapolated Yield
130 mg	410.3 mg	204.3 mg	73.0 mg	169.5 mg	38.4 mg	2 mg	7.6 mg
0.18 mmol / g (23.4 μmol)	50% of total resin		13% Yield		53% of Isolated Peptide	5% Purification Yield	2.1% Overall Yield

<u>*Cleavage Method*</u>: Method 1.1.1 - Reagent K
<u>*Purification Method*</u>: Method 1.4.1 - 2 Stage Semi-Prep
<u>*Purified HPLC Method*</u>: Method 1.2.1 - 5% to 65% in 30 min on Agilent 1200
<u>*Purified LCMS Method*</u>: Method 1.3.1 - 6545 1-91 in 8 min
<u>*Calculated Mass*</u>: 13972 Da
<u>*Observed Mass*</u>: 13972 Da

2.7. CHIP from AFPS

Protein	Uniprot ID	Start	End	Length	Modifications
CHIP	Q9UNE7	23	153	132	N-Term PEG_{12}Biotin, C-Term carboxamide
SPSAQELKEQ GNRLFVGRKY PEAAACYGRA ITRNPLVAVY YTNRALCYLK MQQHEQALAD CRRALELDGQ SVKAHFFLGQ CQLEMESYDE AIANLQRAYS LAKEQRLNFG DDIPSALRIA KKKRWNSIEE RR					

2.7.1. L-CHIP AFPS Results

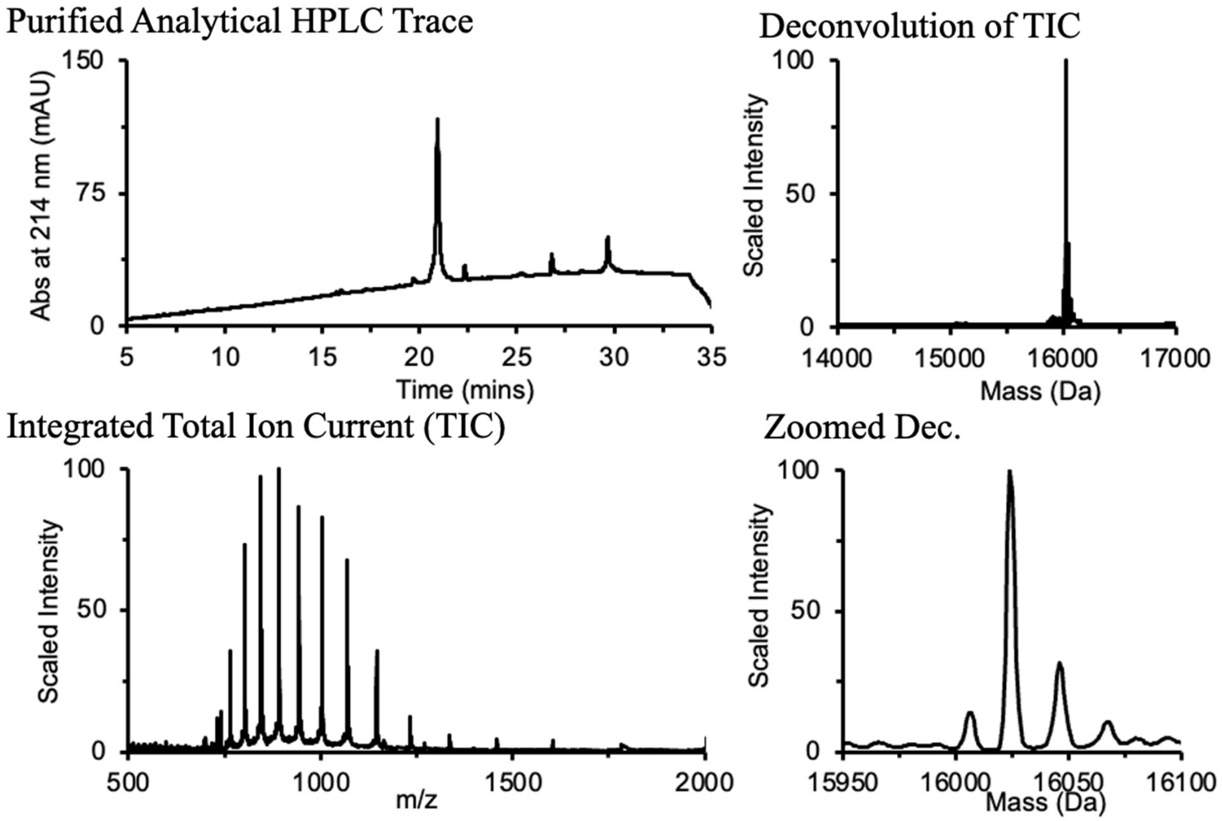

Starting Resin	Crude Resin	Cleaved Resin	Isolated Mass	Theoretical Mass	Purified Mass	Isolated after Purification	Extrapolated Yield
130 mg	587.0 mg	295.6 mg	45.4 mg	207.5 mg	29.4 mg	0.6 mg	1.8 mg
0.18 mmol / g (23.4 μmol)	100% of total resin		22% Yield		65% of Isolated Peptide	2% Purification Yield	0.4% Overall Yield

Cleavage Method: Method 1.1.1 - Reagent K
Purification Method: Method 1.4.1 - 2 Stage Semi-Prep
Purified HPLC Method: Method 1.2.1 - 5% to 65% in 30 min on Agilent 1200
Purified LCMS Method: Method 1.3.1 - 6545 1-91 in 8 min
Calculated Mass: 16025 Da
Observed Mass: 16025 Da

2.7.2. D-CHIP AFPS Results

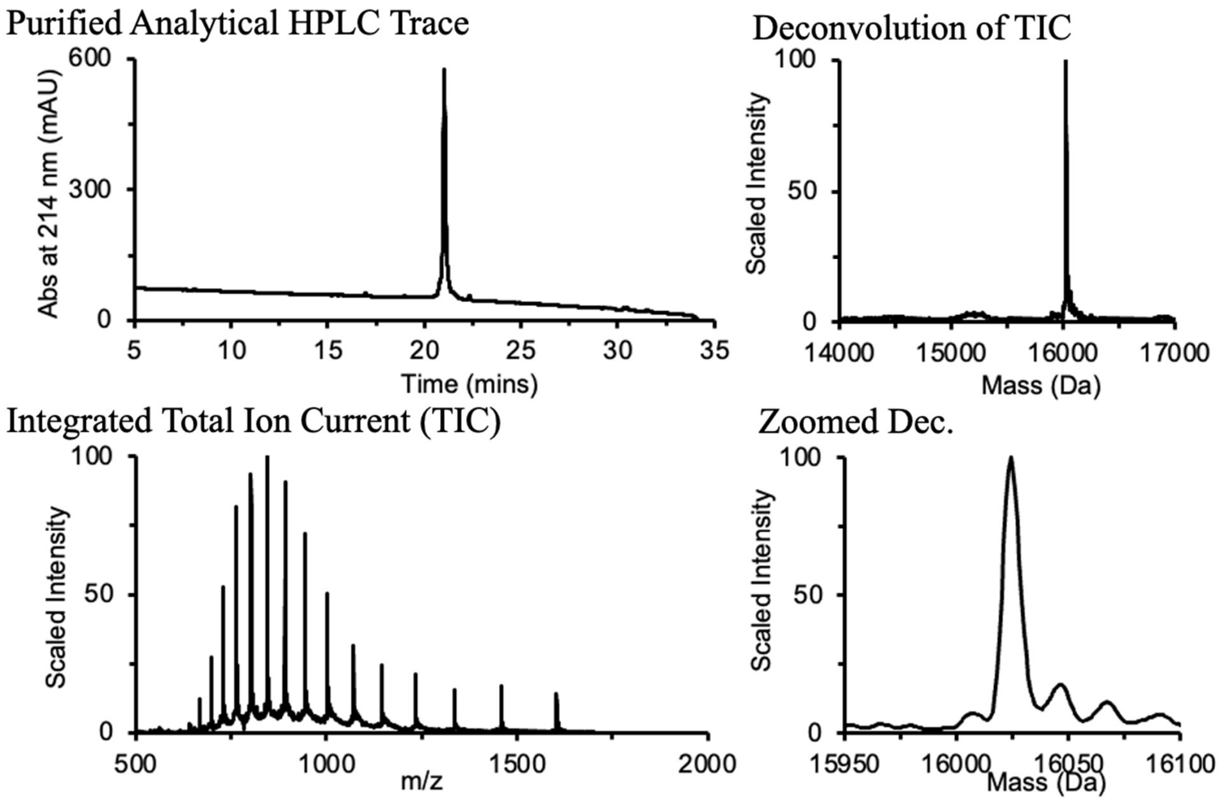

Starting Resin	Crude Resin	Cleaved Resin	Isolated Mass	Theoretical Mass	Purified Mass	Isolated after Purification	Extrapolated Yield
130 mg	n.d.	n.d.	63.1 mg	412.1 mg	63.1 mg	1.6 mg	1.6 mg
0.18 mmol / g (23.4 μmol)	100% of total resin		15% Yield		100% of Isolated Peptide	3% Purification Yield	0.4% Overall Yield

<u>*Cleavage Method*</u>: Method 1.1.1 - Reagent K
<u>*Purification Method*</u>: Method 1.4.1 - 2 Stage Semi-Prep
<u>*Purified HPLC Method*</u>: Method 1.2.1 - 5% to 65% in 30 min on Agilent 1200
<u>*Purified LCMS Method*</u>: Method 1.3.1 - 6545 1-91 in 8 min
<u>*Calculated Mass*</u>: 16025 Da
<u>*Observed Mass*</u>: 16025 Da

2.8. NEMO from AFPS

Protein	Uniprot ID	Start	End	Length	Modifications
NEMO	Q9Y6K9	44	112	70	N-Term PEG_{12}Biotin, C-Term carboxamide, Nle in place of Met
EQGAPETLQR CLEENQELRD AIRQSNQILR ERCEELLHFQ ASQREEKEFL vCKFQEARKL VERLGLEKLE					

2.8.1. L-NEMO AFPS Results

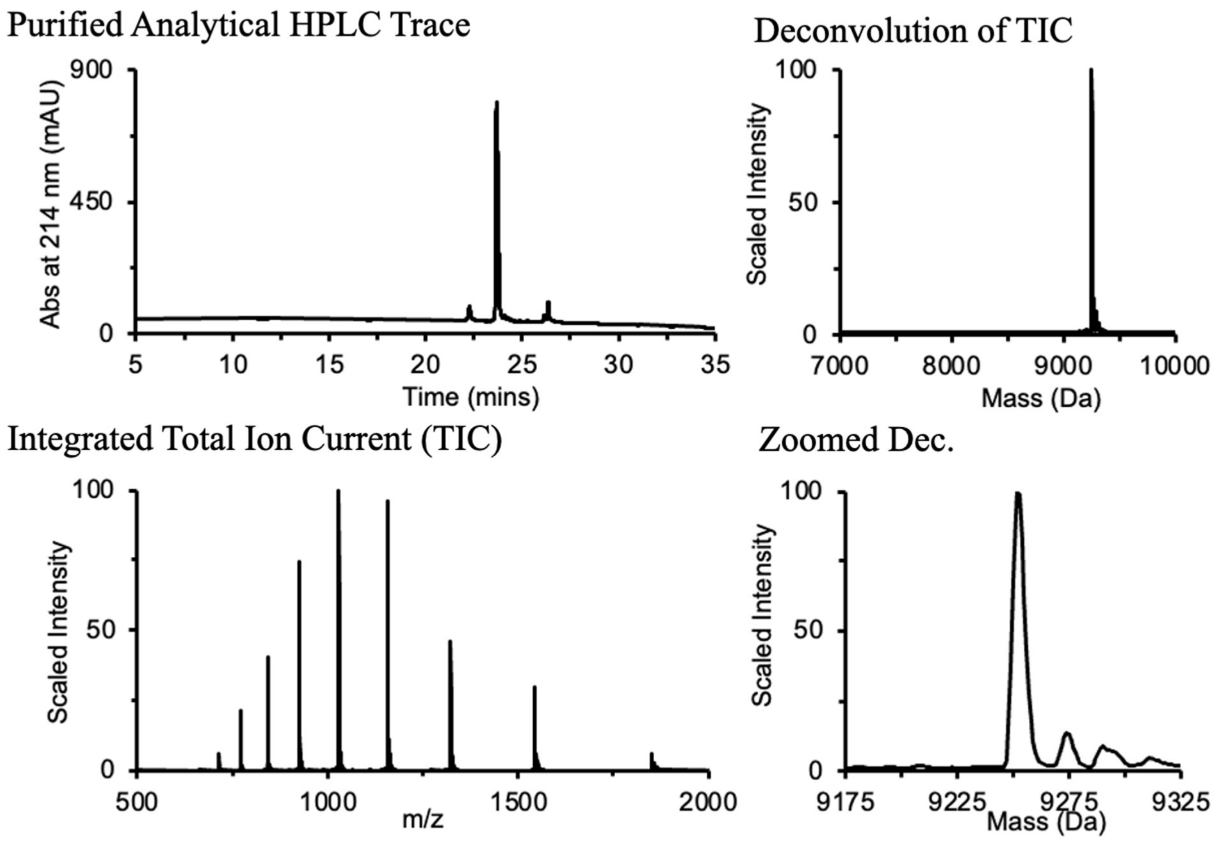

Starting Resin	Crude Resin	Cleaved Resin	Isolated Mass	Theoretical Mass	Purified Mass	Isolated after Purification	Extrapolated Yield
130 mg	n.d.	n.d.	70.5 mg	237.3 mg	20.0 mg	1.6 mg	5.6 mg
0.18 mmol / g (23.4 μmol)	100% of total resin		30% Yield		28% of Isolated Peptide	8% Purification Yield	2.4% Overall Yield

Cleavage Method: Method 1.1.1 - Reagent K
Purification Method: Method 1.4.1 - 2 Stage Semi-Prep
Purified HPLC Method: Method 1.2.1 - 5% to 65% in 30 min on Agilent 1200
Purified LCMS Method: Method 1.3.1 - 6545 1-91 in 8 min
Calculated Mass: 9254 Da
Observed Mass: 9254 Da

2.8.2. D-NEMO AFPS Results

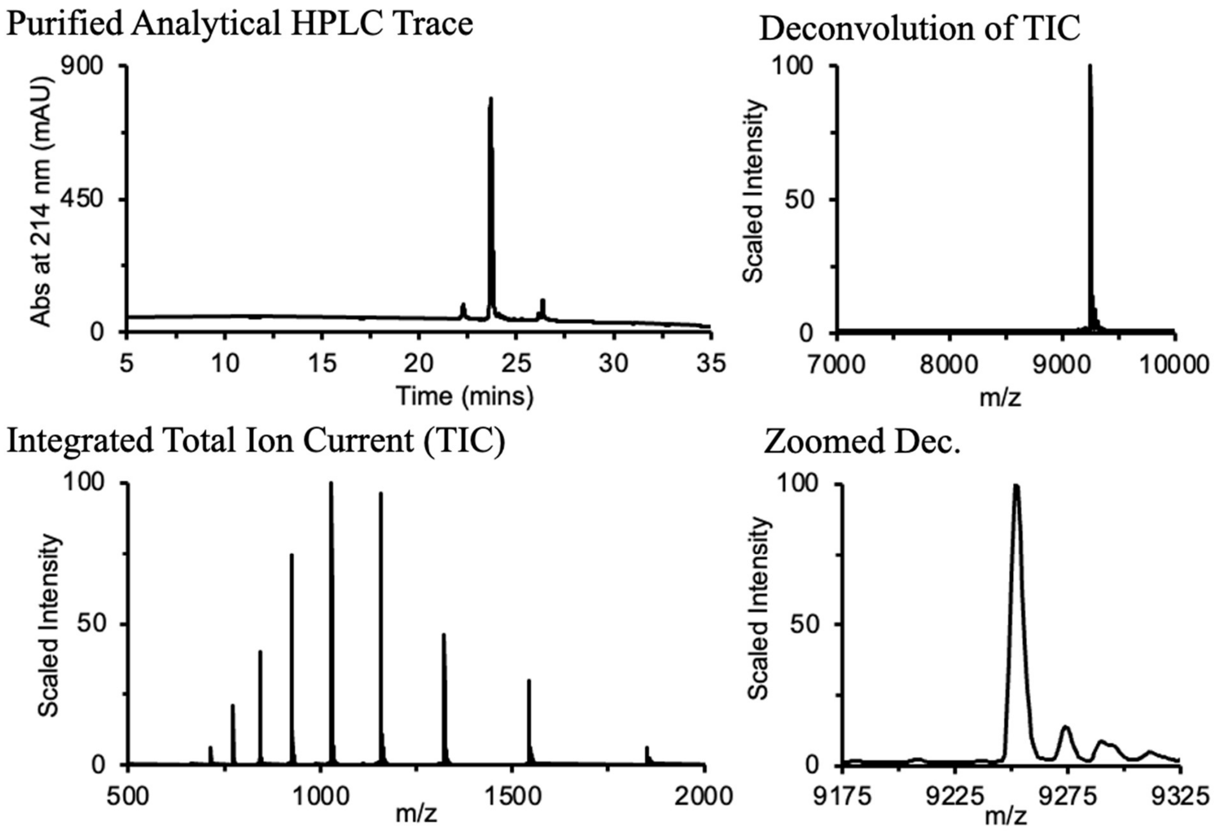

Starting Resin	Crude Resin	Cleaved Resin	Isolated Mass	Theoretical Mass	Purified Mass	Isolated after Purification	Extrapolated Yield
130 mg	n.d.	n.d.	29.7 mg	118.6 mg	29.7 mg	6.5 mg	13.0 mg
0.18 mmol / g (23.4 μmol)	50% of total resin		25% Yield		100% of Isolated Peptide	22% Purification Yield	5.5% Overall Yield

<u>Cleavage Method</u>: Method 1.1.1 - Reagent K
<u>Purification Method</u>: Method 1.4.1 - 2 Stage Semi-Prep
<u>Purified HPLC Method</u>: Method 1.2.1 - 5% to 65% in 30 min on Agilent 1200
<u>Purified LCMS Method</u>: Method 1.3.4 – 6550-2 1-91 in 15 min
<u>Calculated Mass</u>: 9254 Da
<u>Observed Mass</u>: 9254 Da

2.9. FKBP12 from AFPS

Protein	Uniprot ID	Start	End	Length	Modifications
FKBP12	P62942	2	108	107	N-Term PEG_{12}Biotin, C-Term carboxamide
GVQVETISPG DGRTFPKRGQ TCVVHYTGML EDGKKFDSSR DRNKPFKFML GKQEVIRGWE EGVAQMSVGQ RAKLTISPDY AYGATGHPGI IPPHATLVFD VELLKLE					

2.9.1. L-FKBP12 AFPS Results

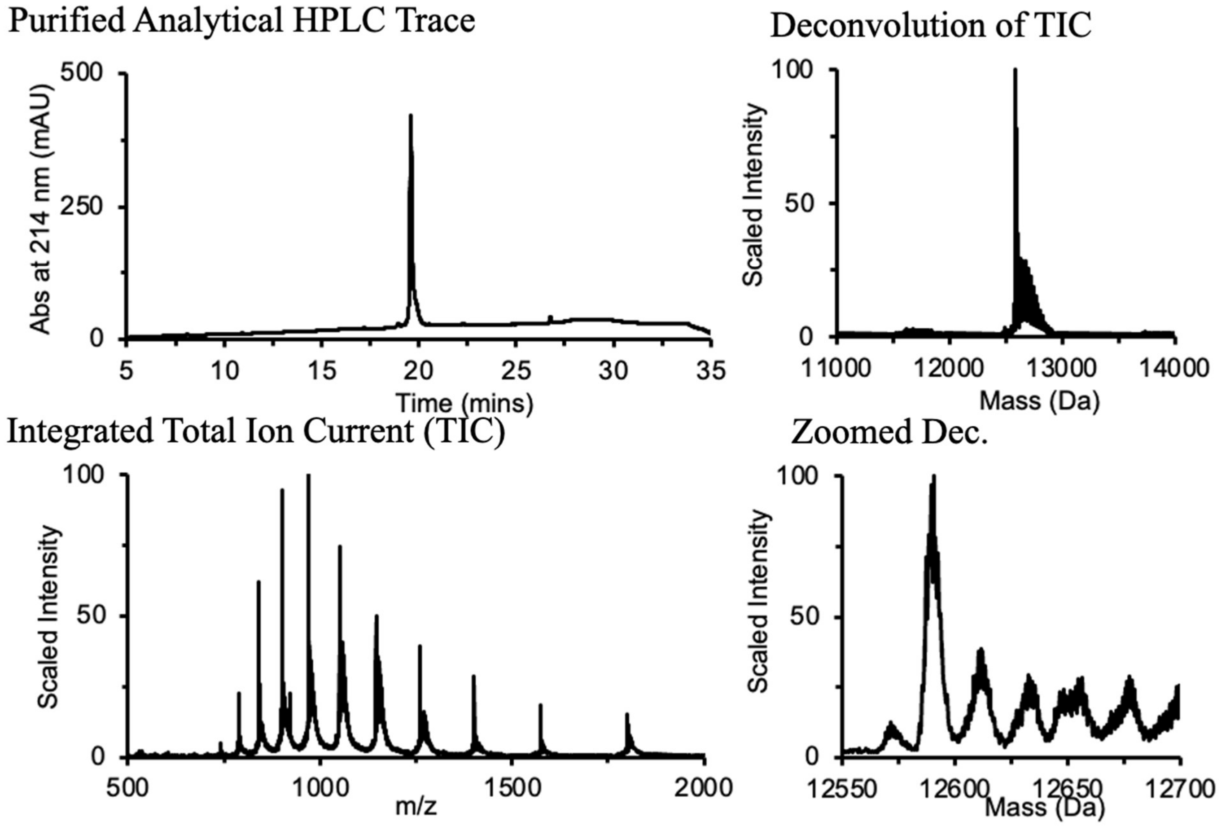

Starting Resin	Crude Resin	Cleaved Resin	Isolated Mass	Theoretical Mass	Purified Mass	Isolated after Purification	Extrapolated Yield
130 mg	n.d.	n.d.	95.3 mg	321.1 mg	30.6 mg	2.4 mg	7.5 mg
0.18 mmol / g (23.4 μmol)	100% of total resin		30% Yield		32% of Isolated Peptide	8% Purification Yield	2.3% Overall Yield

<u>*Cleavage Method*</u>: Method 1.1.1 - Reagent K
<u>*Purification Method*</u>: Method 1.4.1 - 2 Stage Semi-Prep
<u>*Purified HPLC Method*</u>: Method 1.2.1 - 5% to 65% in 30 min on Agilent 1200
<u>*Purified LCMS Method*</u>: Method 1.3.4 – 6550-2 1-91 in 15 min
<u>*Calculated Mass*</u>: 12593 Da
<u>*Observed Mass*</u>: 12593 Da

2.9.2. D-FKBP12 AFPS Results

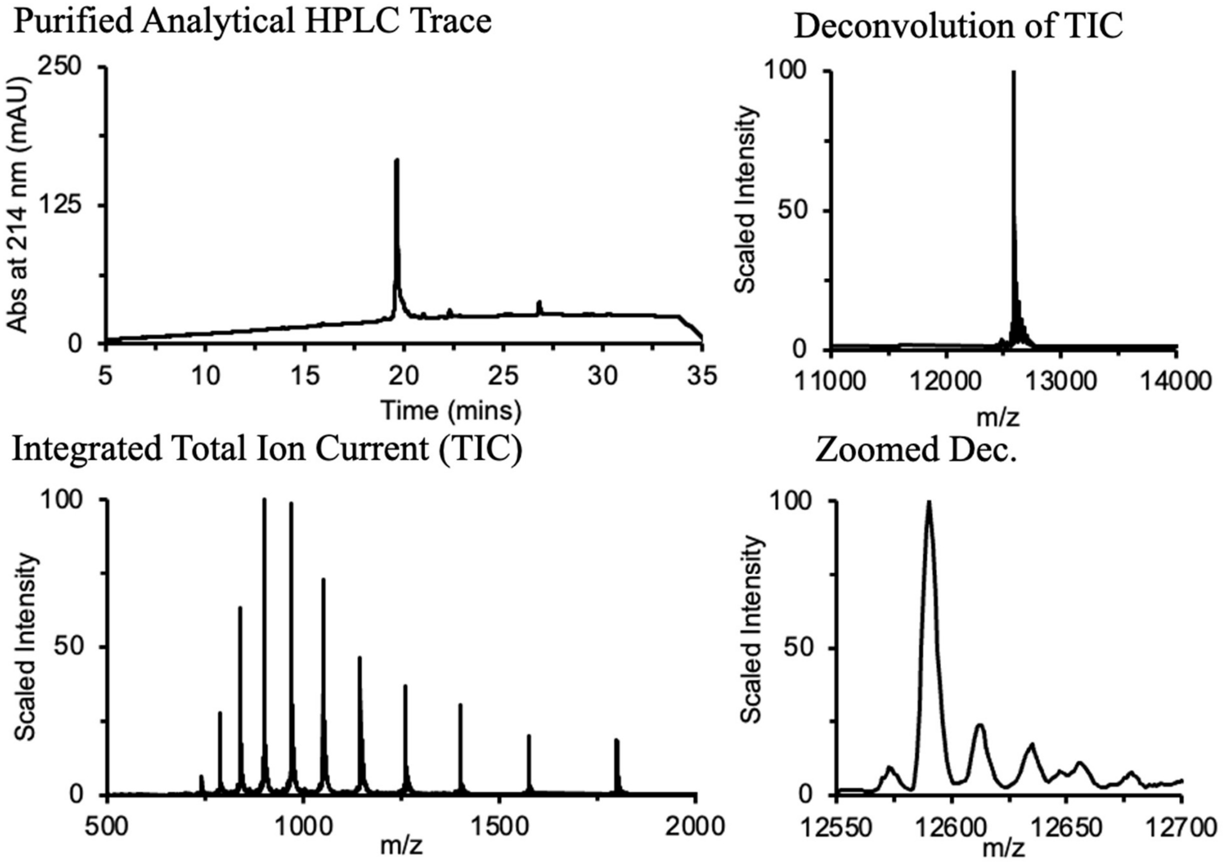

Starting Resin	Crude Resin	Cleaved Resin	Isolated Mass	Theoretical Mass	Purified Mass	Isolated after Purification	Extrapolated Yield
130 mg	n.d.	n.d.	89.5 mg	321.1 mg	35.8 mg	2.2 mg	6 mg
0.18 mmol / g (23.4 μmol)	100% of total resin		28% Yield		40% of Isolated Peptide	6% Purification Yield	1.7% Overall Yield

<u>*Cleavage Method*</u>: Method 1.1.1 - Reagent K
<u>*Purification Method*</u>: Method 1.4.1 - 2 Stage Semi-Prep
<u>*Purified HPLC Method*</u>: Method 1.2.1 - 5% to 65% in 30 min on Agilent 1200
<u>*Purified LCMS Method*</u>: Method 1.3.1 - 6545 1-91 in 8 min
<u>*Calculated Mass*</u>: 12593 Da
<u>*Observed Mass*</u>: 12593 Da

2.10. BCL11a from AFPS

Protein	Uniprot ID	Start	End	Length	Modifications
BCL11a	Q9H165	740	835	96	N-Term PEG_{12}Biotin, C-Term carboxamide, Nle in place of Met
RSDTCEYCGK VFKNCSNLTV HRRSHTGERP YKCELCNYAC AQSSKLTRHM KTHGQVGKDV YKCEICKMPF SVYSTLEKHM KKWHSDRVLN NDIKTE					

2.10.1. L-BCL11a AFPS Results

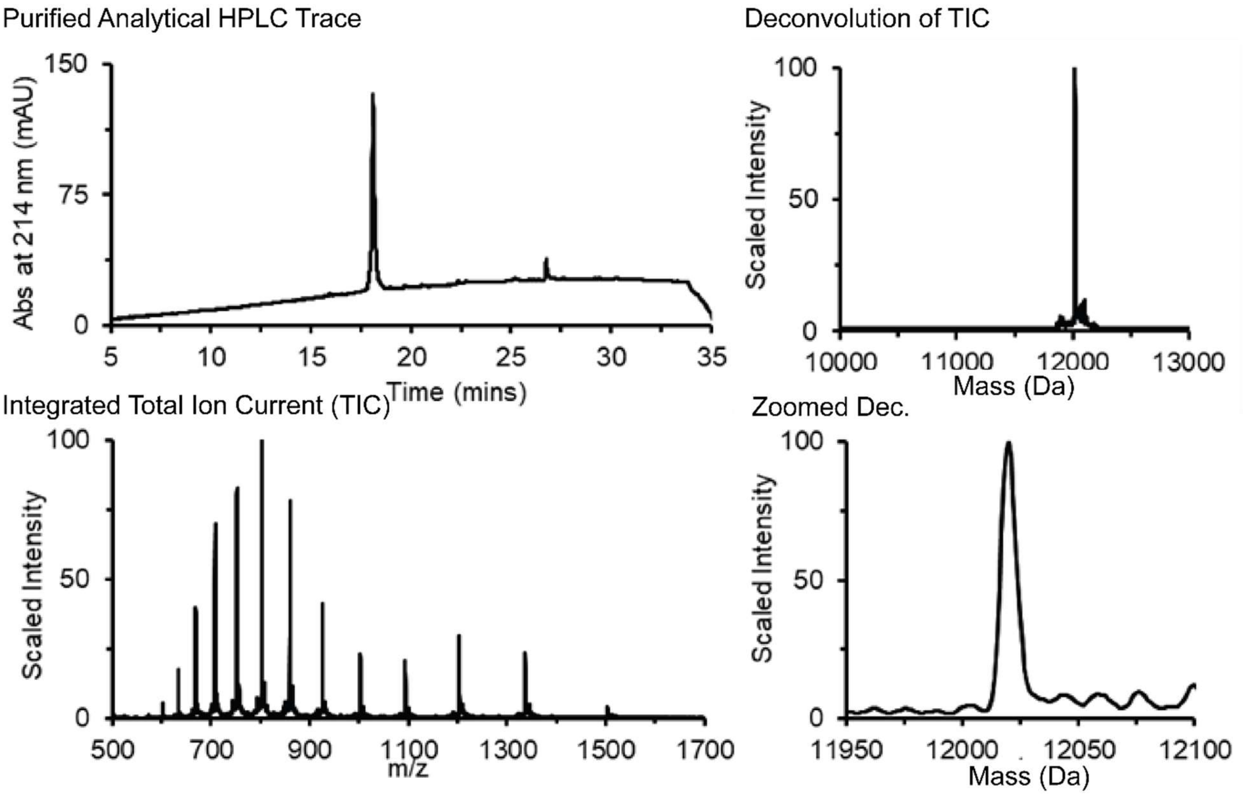

Starting Resin	Crude Resin	Cleaved Resin	Isolated Mass	Theoretical Mass	Purified Mass	Isolated after Purification	Extrapolated Yield
130 mg	n.d.	n.d.	31.0 mg	163.1 mg	31.0 mg	5.2 mg	10.4 mg
0.18 mmol / g (23.4 μmol)	50% of total resin		19% Yield		100% of Isolated Peptide	17% Purification Yield	3.2% Overall Yield

<u>*Cleavage Method*</u>: Method 1.1.1 - Reagent K
<u>*Purification Method*</u>: Method 1.4.1 - 2 Stage Semi-Prep
<u>*Purified HPLC Method*</u>: Method 1.2.1 - 5% to 65% in 30 min on Agilent 1200
<u>*Purified LCMS Method*</u>: Method 1.3.3 - 6550-1 5-95 in 10 min
<u>*Calculated Mass*</u>: 12022 Da
<u>*Observed Mass*</u>: 12022 Da

2.10.2. D-BCL11a AFPS Results

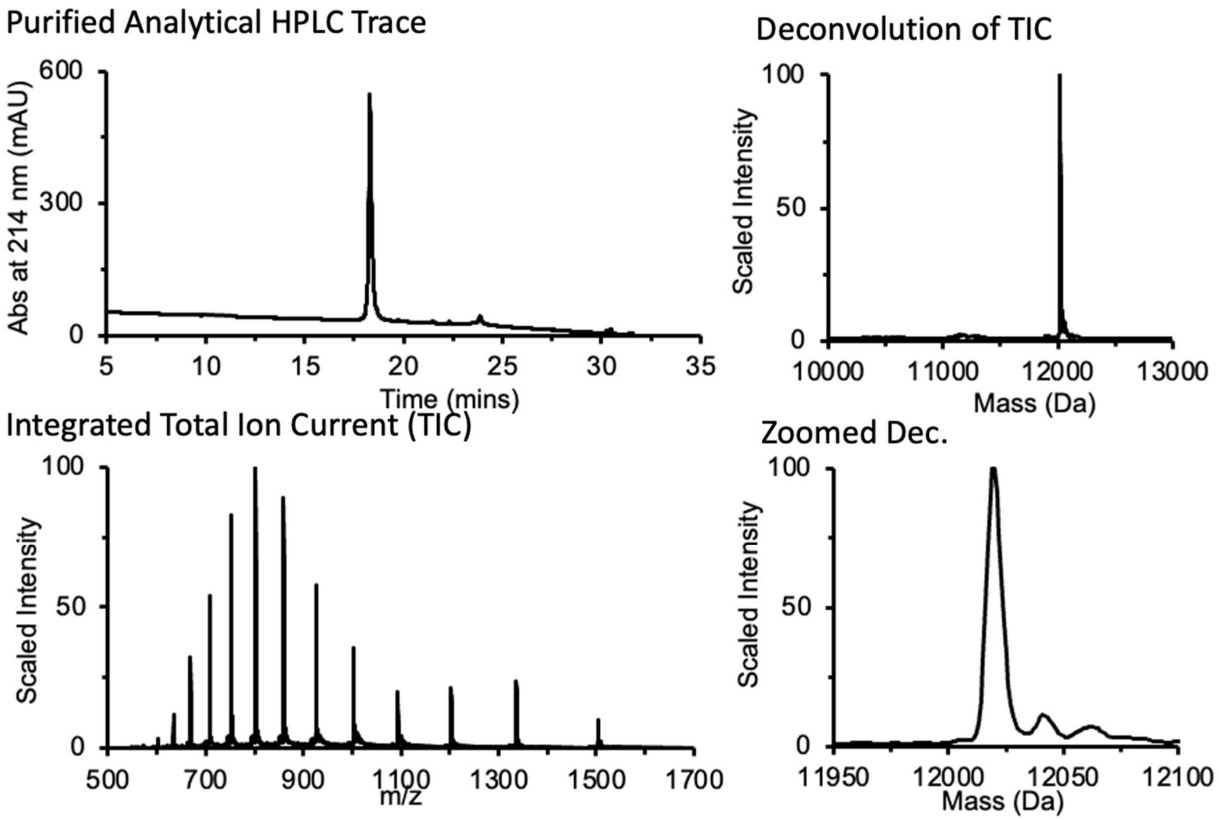

Starting Resin	Crude Resin	Cleaved Resin	Isolated Mass	Theoretical Mass	Purified Mass	Isolated after Purification	Extrapolated Yield
130 mg	n.d.	n.d.	60.0 mg	326.1 mg	60.0 mg	5.6 mg	5.6 mg
0.18 mmol / g (23.4 μmol)	100% of total resin		18% Yield		100% of Isolated Peptide	9% Purification Yield	1.7% Overall Yield

<u>*Cleavage Method*</u>: Method 1.1.1 - Reagent K
<u>*Purification Method*</u>: Method 1.4.1 - 2 Stage Semi-Prep
<u>*Purified HPLC Method*</u>: Method 1.2.1 - 5% to 65% in 30 min on Agilent 1200
<u>*Purified LCMS Method*</u>: Method 1.3.3 - 6550-1 5-95 in 10 min
<u>*Calculated Mass*</u>: 12022 Da
<u>*Observed Mass*</u>: 12022 Da

2.11. YAP1 from AFPS

Protein	Uniprot ID	Start	End	Length	Modifications
Yap1	P46937	163	276	114	N-Term PEG_{12}Biotin, C-Term carboxamide, Nle in place of Met
SSFEIPDDVP LPAGWEMAKT SSGQRYFLNH IDQTTTWQDP RKAMLSQMNV TAPTSPPVQQ NMMNSASGPL PDGWEQAMTQ DGEIYYINHK NKTTSWLDPR LDPRFAMNQR ISQS					

2.11.1. L-YAP1 AFPS Results

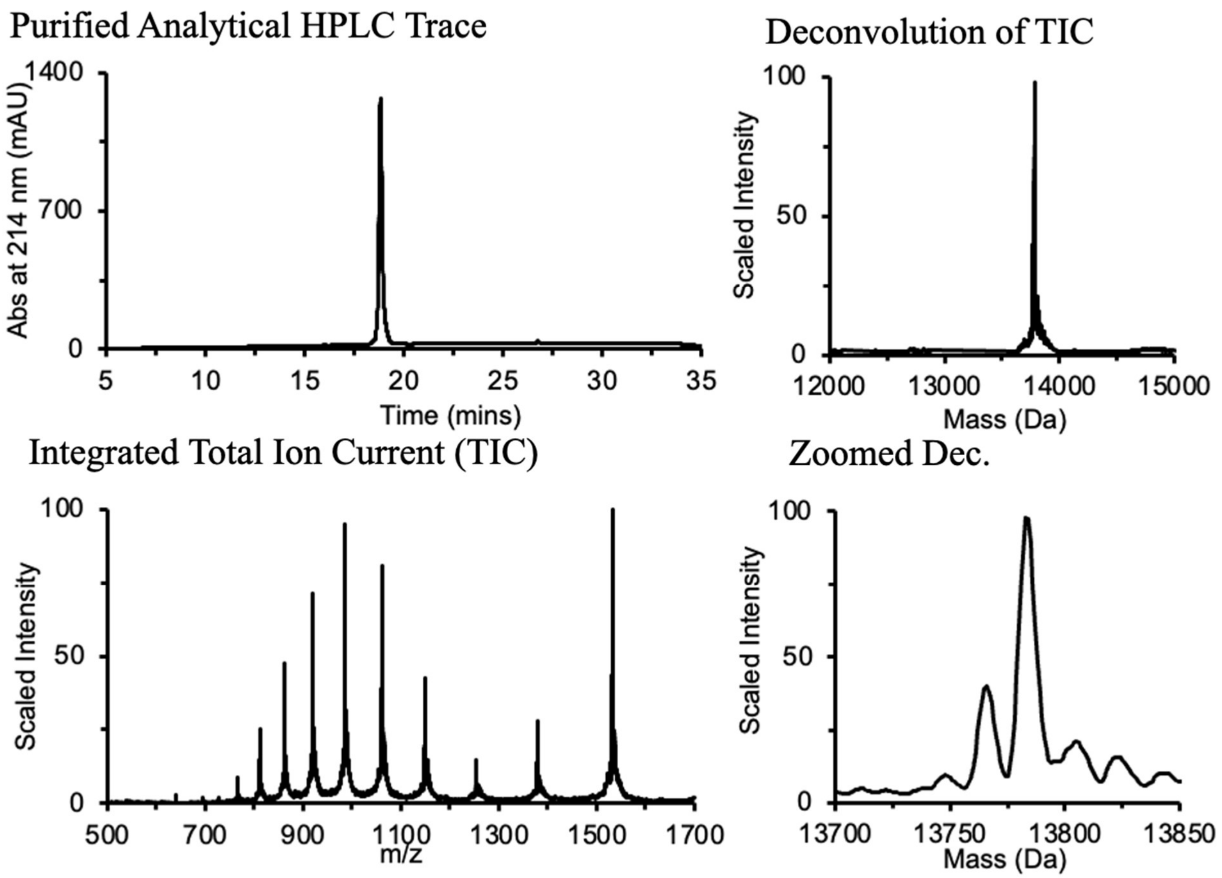

Starting Resin	Crude Resin	Cleaved Resin	Isolated Mass	Theoretical Mass	Purified Mass	Isolated after Purification	Extrapolated Yield
130 mg	n.d.	n.d.	126.3 mg	332.4 mg	50.2 mg	1.1 mg	2.8 mg
0.18 mmol / g (23.4 μmol)	100% of total resin		38% Yield		40% of Isolated Peptide	2% Purification Yield	0.8% Overall Yield

Cleavage Method: Method 1.1.1 - Reagent K
Purification Method: Method 1.4.1 - 2 Stage Semi-Prep
Purified HPLC Method: Method 1.2.1 - 5% to 65% in 30 min on Agilent 1200
Purified LCMS Method: Method 1.3.3 - 6550-1 5-95 in 10 min
Calculated Mass: 13784 Da
Observed Mass: 13784 Da

2.11.2. D-YAP1 AFPS Results

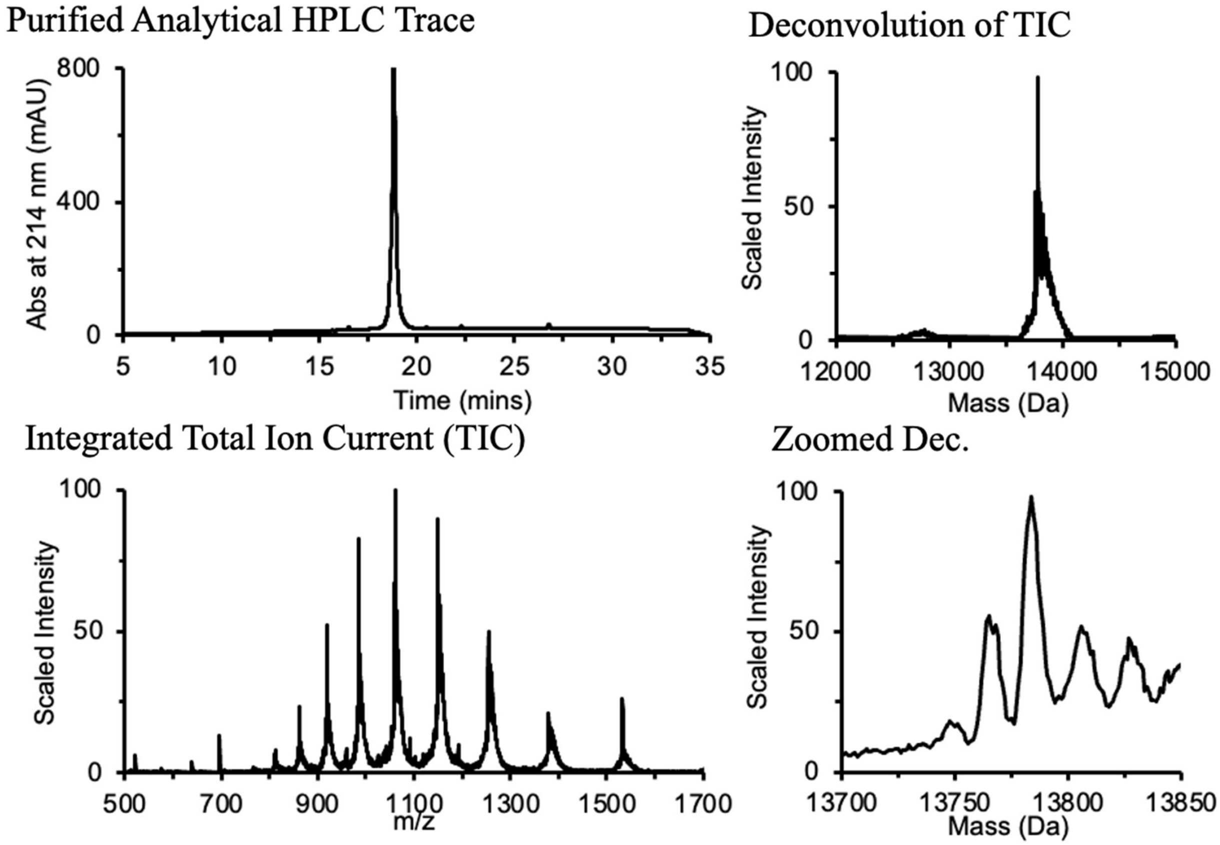

Starting Resin	Crude Resin	Cleaved Resin	Isolated Mass	Theoretical Mass	Purified Mass	Isolated after Purification	Extrapolated Yield
130 mg	n.d.	n.d.	90.4 mg	332.4 mg	50.2 mg	2.6 mg	6.1 mg
0.18 mmol / g (23.4 μmol)	100% of total resin		27% Yield		43% of Isolated Peptide	7% Purification Yield	1.8% Overall Yield

<u>Cleavage Method</u>: Method 1.1.1 - Reagent K
<u>Purification Method</u>: Method 1.4.1 - 2 Stage Semi-Prep
<u>Purified HPLC Method</u>: Method 1.2.1 - 5% to 65% in 30 min on Agilent 1200
<u>Purified LCMS Method</u>: Method 1.3.3 - 6550-1 5-95 in 10 min
<u>Calculated Mass</u>: 13784 Da
<u>Observed Mass</u>: 13784 Da

2.12. NEMO_iZIP from AFPS

Protein	Uniprot ID	Start	End	Length	Modifications
Nemo_iZip	*6MI4	4	124	121	N-Term PEG_{12}Biotin, C-Term carboxamide, Nle in place of Met
SVKELEDKNE ELLSEIAHLK NEVARLKKLL QRCLEENQEL RDAIRQSNQI LRERCEELLH FQASQREEKE FLMCKFQEAR KLVERLGLEK LELEDKNEEL LSEIAHLKNE VARLKKLVGE R					

2.12.1. L-NEMO_iZIP AFPS Results

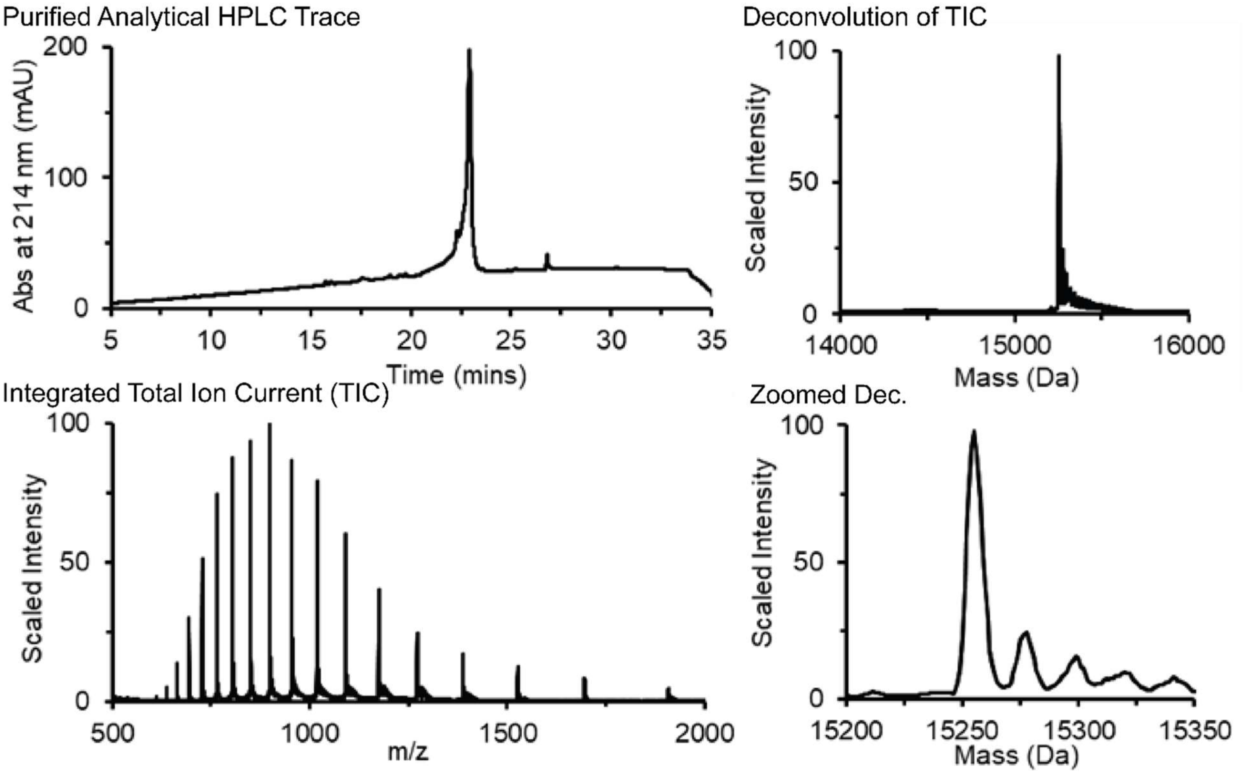

Starting Resin	Crude Resin	Cleaved Resin	Isolated Mass	Theoretical Mass	Purified Mass	Isolated after Purification	Extrapolated Yield
130 mg	n.d.	n.d.	40.3 mg	202.6 mg	40.3 mg	0.9 mg	1.8 mg
0.18 mmol / g (23.4 μmol)	50% of total resin		20% Yield		100% of Isolated Peptide	2% Purification Yield	0.4% Overall Yield

Cleavage Method: Method 1.1.1 - Reagent K
Purification Method: Method 1.4.1 - 2 Stage Semi-Prep
Purified HPLC Method: Method 1.2.1 - 5% to 65% in 30 min on Agilent 1200
Purified LCMS Method: Method 1.3.4 – 6550-2 1-91 in 15 min
Calculated Mass: 15256 Da
Observed Mass: 15256 Da

2.12.2. D-NEMO_iZIP AFPS Results

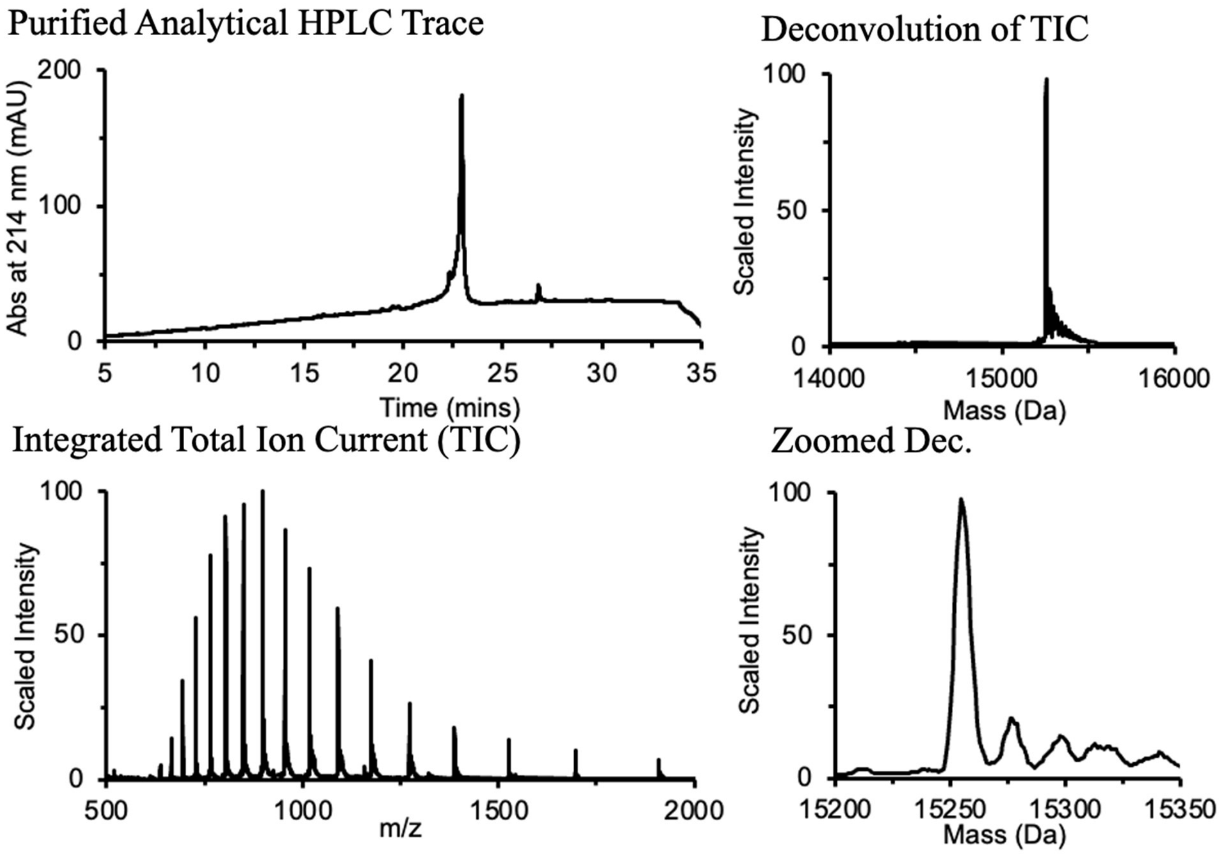

Starting Resin	Crude Resin	Cleaved Resin	Isolated Mass	Theoretical Mass	Purified Mass	Isolated after Purification	Extrapolated Yield
130 mg	n.d.	n.d.	52.4 mg	202.6 mg	52.4 mg	2.0 mg	4.0 mg
0.18 mmol / g (23.4 μmol)	50% of total resin		26% Yield		100% of Isolated Peptide	4% Purification Yield	1.0% Overall Yield

<u>*Cleavage Method*</u>: Method 1.1.1 - Reagent K
<u>*Purification Method*</u>: Method 1.4.1 - 2 Stage Semi-Prep
<u>*Purified HPLC Method*</u>: Method 1.2.1 - 5% to 65% in 30 min on Agilent 1200
<u>*Purified LCMS Method*</u>: Method 1.3.4 – 6550-2 1-91 in 15 min
<u>*Calculated Mass*</u>: 15256 Da
<u>*Observed Mass*</u>: 15256 Da

2.13. Max from AFPS

Protein	Uniprot ID	Start	End	Length	Modifications
Max	P61244	23	102	83	N-Term PEG$_{12}$Biotin, C-Term +GGC + carboxamide, Nle in place of Met
DKRAHHNALE RKRRDHIKDS FHSLRDSVPS LQGEKASRAQ ILDKATEYIQ YMRRKNHTHQ QDIDDLKRQN ALLEQQVRAL GGC					

2.13.1. L-Max AFPS Results

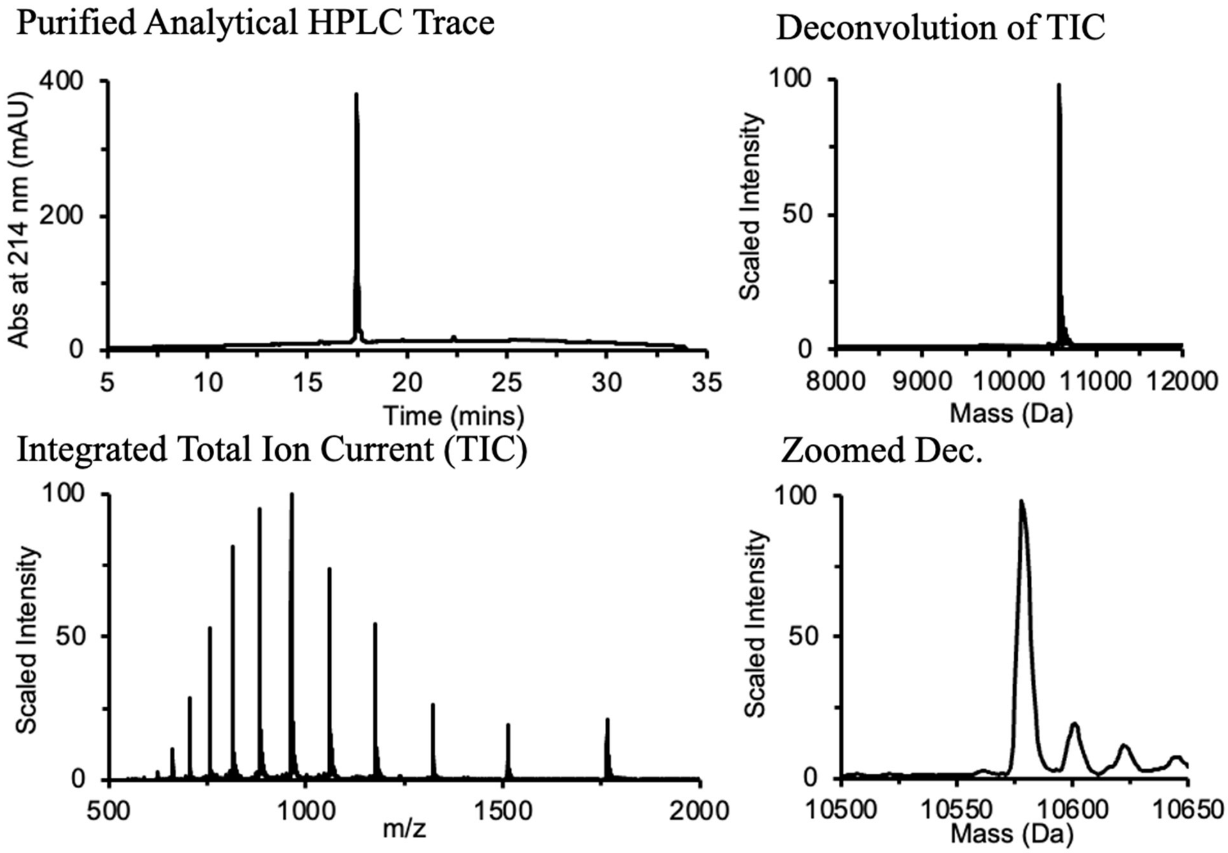

Starting Resin	Crude Resin	Cleaved Resin	Isolated Mass	Theoretical Mass	Purified Mass	Isolated after Purification	Extrapolated Yield
85 mg	nd	nd	208.7 mg	550 mg	161.8 mg	25.8 mg	33.0 mg
0.49 mmol / g (41.6 μmol)	100% of total resin		35% Yield		78% of Isolated Peptide	16% Purification Yield	6.1% Overall Yield

Cleavage Method: Method 1.1.2
Purification Method: Method 1.4.2 - 2 Stage Prep
Purified HPLC Method: Method 1.2.1 - 5% to 65% in 30 min on Agilent 1200
Purified LCMS Method: Method 1.3.1 - 6545 1-91 in 8 min
Calculated Mass: 10580 Da
Observed Mass: 10580 Da

2.13.2. D-Max AFPS Results

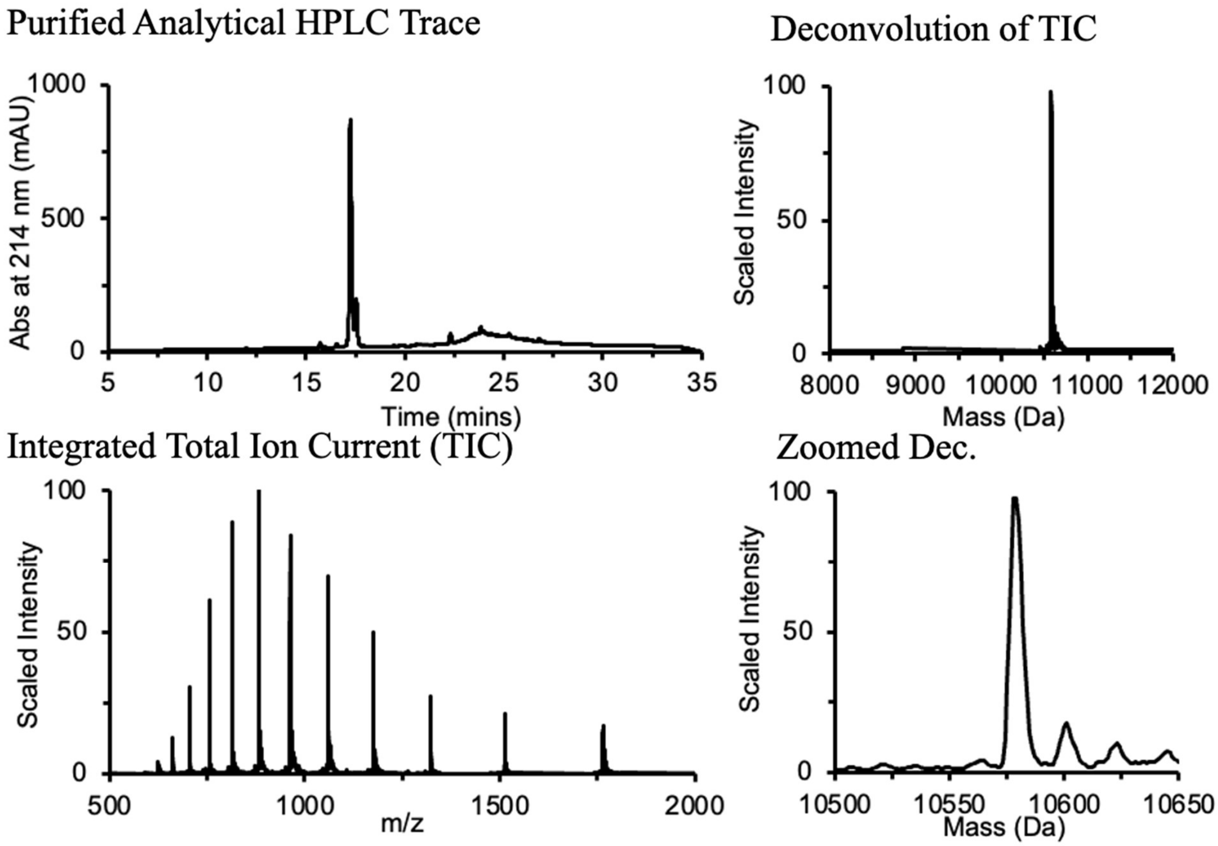

Starting Resin	Crude Resin	Cleaved Resin	Isolated Mass	Theoretical Mass	Purified Mass	Isolated after Purification	Extrapolated Yield
130 mg	n.d.	n.d.	113.2 mg	275.3 mg	113.2 mg	7.0 mg	13.9 mg
0.18 mmol / g (23.4 μmol)	50% of total resin		41% Yield		100% of Isolated Peptide	6% Purification Yield	2.5% Overall Yield

<u>Cleavage Method</u>: Method 1.1.1 - Reagent K
<u>Purification Method</u>: Method 1.4.1 - 2 Stage Semi-Prep
<u>Purified HPLC Method</u>: Method 1.2.1 - 5% to 65% in 30 min on Agilent 1200
<u>Purified LCMS Method</u>: Method 1.3.1 - 6545 1-91 in 8 min
<u>Calculated Mass</u>: 10580 Da
<u>Observed Mass</u>: 10580 Da

2.14. Max-nb from AFPS

Protein	Uniprot ID	Start	End	Length	Modifications
Max-nb	P61244	23	102	83	C-Term +GGC + carboxamide, Nle in place of Met
DKRAHHNALE RKRRDHIKDS FHSLRDSVPS LQGEKASRAQ ILDKATEYIQ YMRRKNHTHQ QDIDDLKRQN ALLEQQVRAL GGC					

2.14.1. L-Max-nb AFPS Results

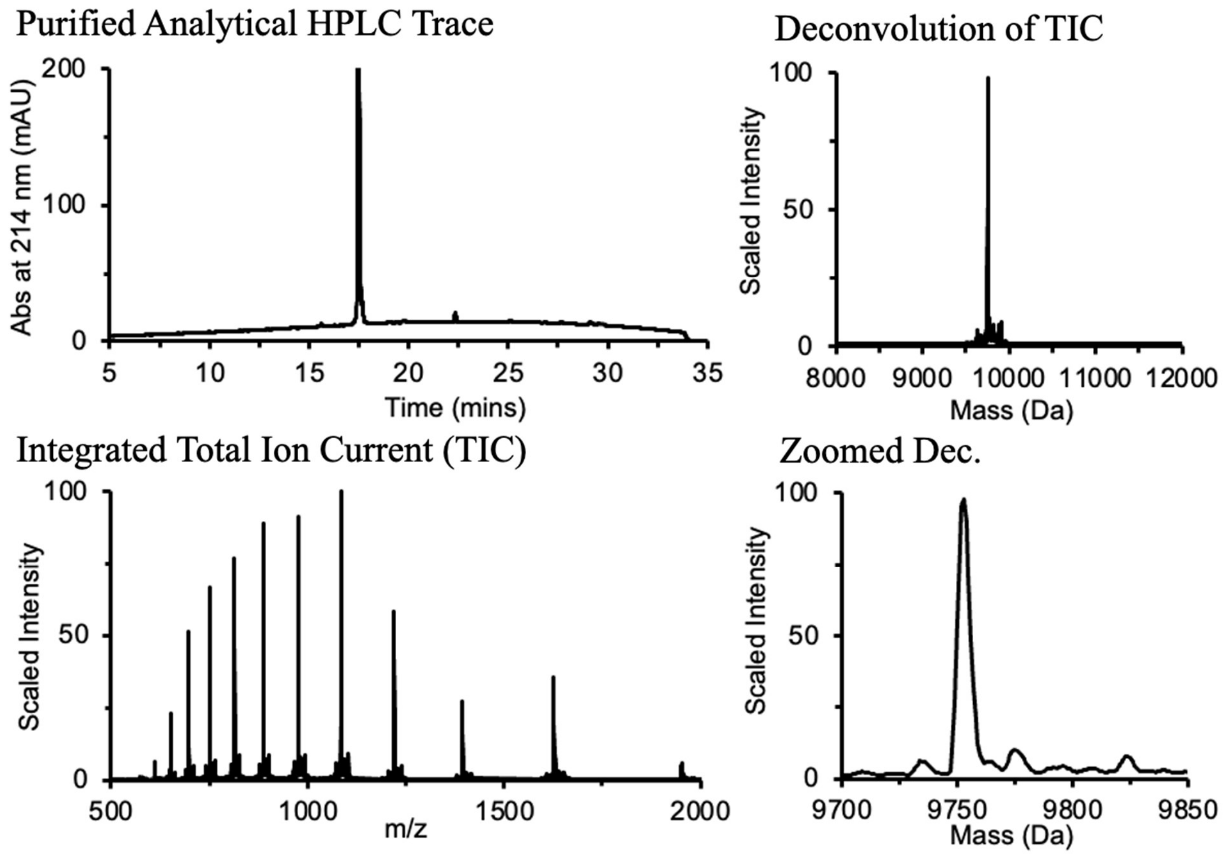

Starting Resin	Crude Resin	Cleaved Resin	Isolated Mass	Theoretical Mass	Purified Mass	Isolated after Purification	Extrapolated Yield
85 mg	n.d.	n.d.	229.4 mg	515 mg	148.7 mg	30.4 mg	46.9 mg
0.49 mmol / g (41.6 μmol)	100% of total resin		45% Yield		65% of Isolated Peptide	20% Purification Yield	9.1% Overall Yield

<u>*Cleavage Method*</u>: Method 1.1.2
<u>*Purification Method*</u>: Method 1.4.2 - 2 Stage Prep
<u>*Purified HPLC Method*</u>: Method 1.2.1 - 5% to 65% in 30 min on Agilent 1200
<u>*Purified LCMS Method*</u>: Method 1.3.1 - 6545 1-91 in 8 min
<u>*Calculated Mass*</u>: 9753 Da
<u>*Observed Mass*</u>: 9753 Da

__2.14.2. D-Max-nb AFPS Results__

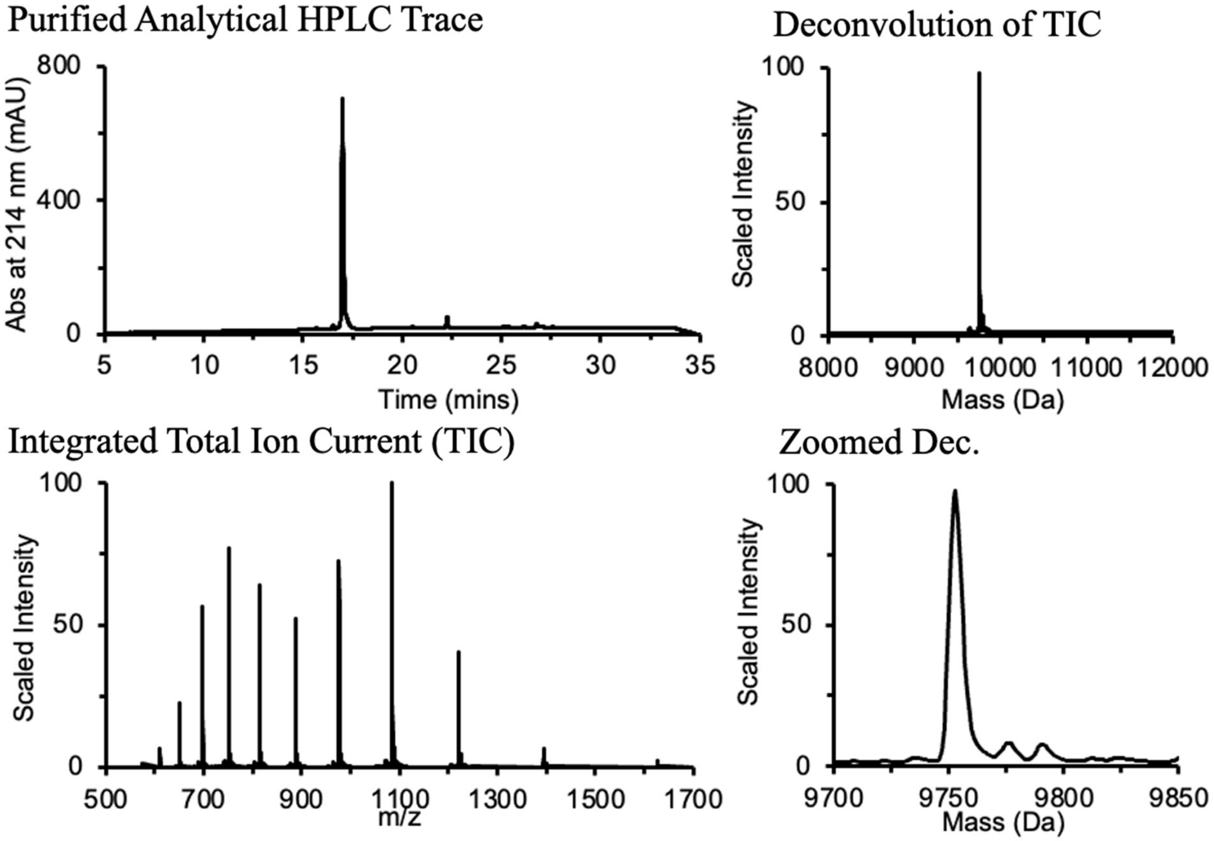

Starting Resin	Crude Resin	Cleaved Resin	Isolated Mass	Theoretical Mass	Purified Mass	Isolated after Purification	Extrapolated Yield
130 mg	n.d.	n.d.	76.8 mg	258.1 mg	76.8 mg	7.3 mg	14.6 mg
0.18 mmol / g (23.4 μmol)	50% of total resin		30% Yield		100% of Isolated Peptide	10% Purification Yield	2.8% Overall Yield

<u>*Cleavage Method*</u>: Method 1.1.1 - Reagent K
<u>*Purification Method*</u>: Method 1.4.1 - 2 Stage Semi-Prep
<u>*Purified HPLC Method*</u>: Method 1.2.1 - 5% to 65% in 30 min on Agilent 1200
<u>*Purified LCMS Method*</u>: Method 1.3.3 - 6550-1 5-95 in 10 min
<u>*Calculated Mass*</u>: 9753 Da
<u>*Observed Mass*</u>: 9753 Da

2.15. Max Oxidative Addition Complex Synthesis

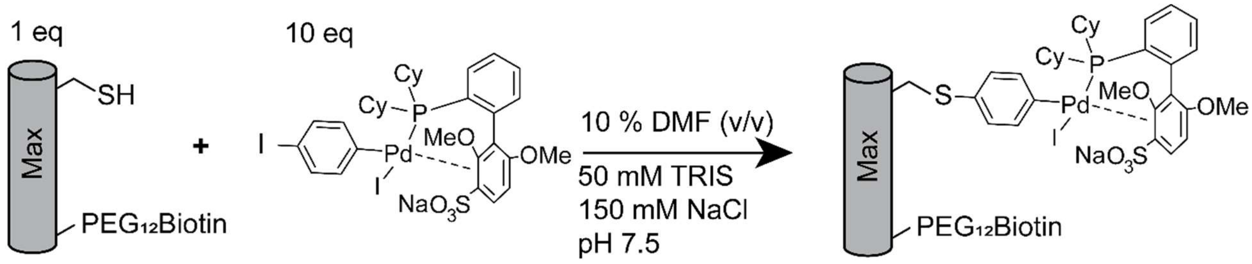

Synthesis of Max-OAC. The protocol for OAC generation was adapted from an earlier report.[5] To a 50 mL Falcon tube was added the purified Max as a lyophilized powder and sufficient reaction buffer (50 mM TRIS HCl, 150 mM NaCl, pH 7.5) was added to prepare a solution at 2 mg / mL (205 μM). Protein concentration was measured with **Method 1.6.1**, and the concentration of the protein stock adjusted to 1.1 mg/mL (113 μM) with addition of reaction buffer. Separately, to a 2.0 mL Eppendorf tube was added 10 equiv of sSPhos reinsertion reagent relative to the protein and dissolved in DMF to 4.8 mg/mL (6 mM). The sSPhos reinsertion reagent in DMF was added to the protein stock, the Falcon tube quickly recapped, and the apparatus vortexed. After incubation for 5 min, the Falcon tube screw cap was removed, and the mixture was transferred to a 20 mL syringe affixed with a 0.22 μM nylon syringe filter. The mixture was forced through the syringe filter into a fresh Falcon tube. The Falcon tube originally containing the un-filtered peptide solution was rinsed with an additional 2 mL of the reaction buffer that was then filtered using the same filtration apparatus into the Falcon tube containing the filtered peptide solution. Any bubbles generated were removed by centrifugation at 3220 × g. The peptide mixture was then purified according to Method 1.4.3.

2.15.1. L-Max Oxidative Addition Complex Results

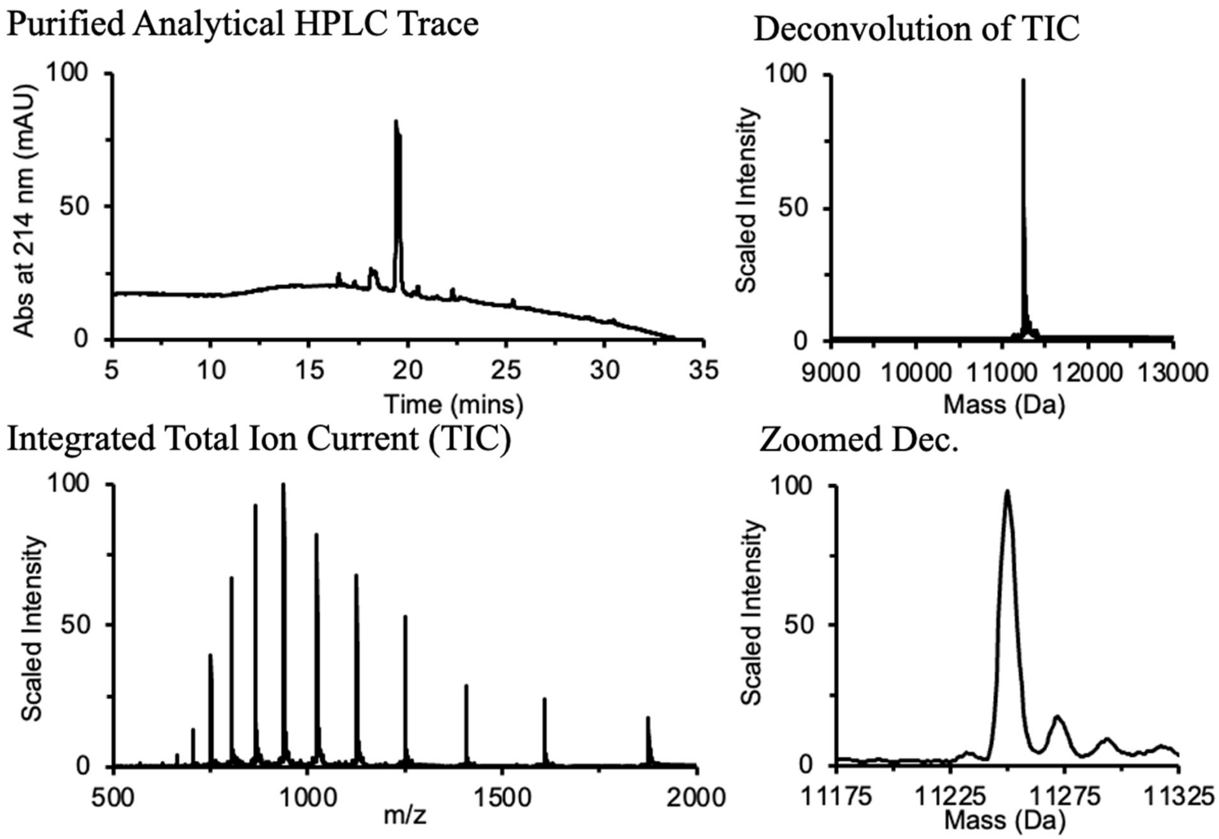

Starting Mass	Purified Mass	Isolated after Purification
11.5 mg	100% of reaction mix	2.2 mg
45% of iso L-Max		18.2% Yield

Purification Method: Method 1.4.3 - 1-Stage Semiprep
Purified HPLC Method: Method 1.2.3 - 5% to 65% in 30 min on Agilent 1290
Purified LCMS Method: Method 1.3.1 - 6545 1-91 in 8 min
Calculated Mass: 11253 Da (Halide ligand to Pd is lost on ionization)
Observed Mass: 11253 Da

2.15.2. D-Max Oxidative Addition Complex Results

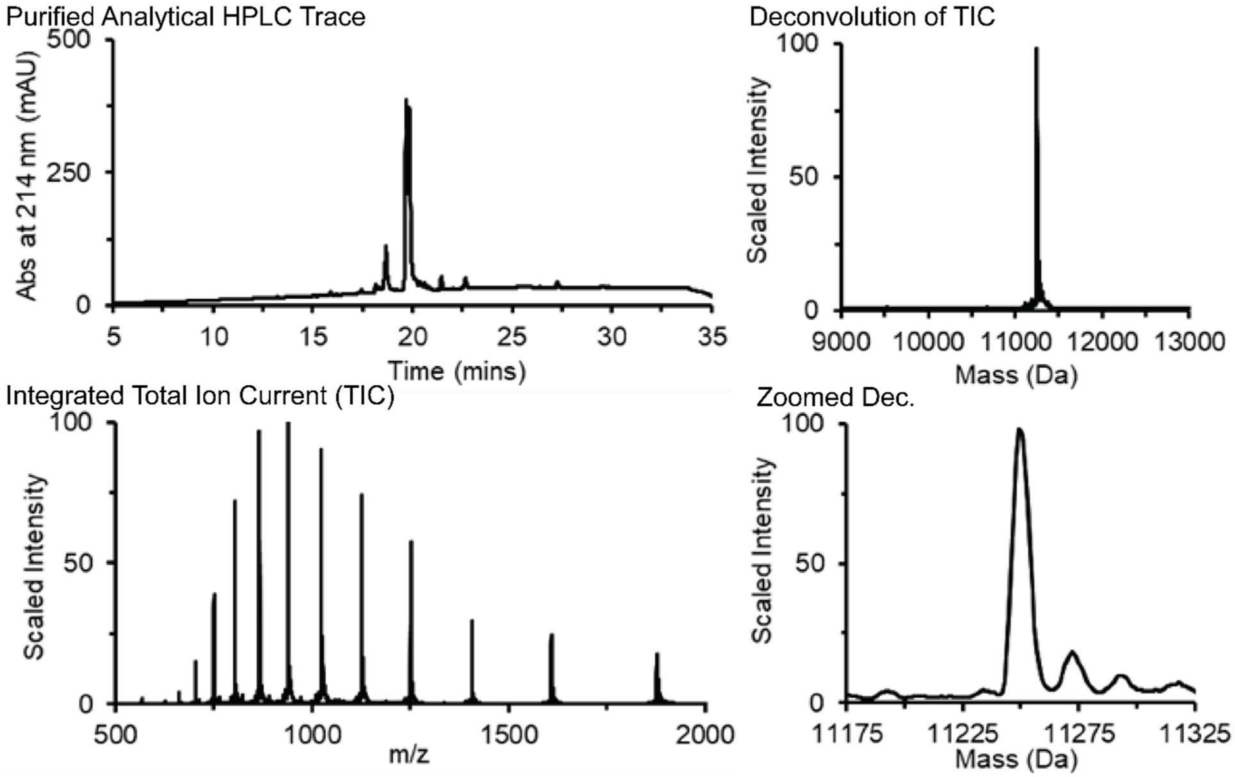

Starting Mass	Purified Mass	Isolated after Purification
6.9 mg	100% of reaction mix	1.1 mg
100% of iso D-Max		15.2% Yield

<u>*Purification Method*</u>: Method 1.4.3 - 1-Stage Semiprep
<u>*Purified HPLC Method*</u>: Method 1.2.3 - 5% to 65% in 30 min on Agilent 1290
<u>*Purified LCMS Method*</u>: Method 1.3.1 - 6545 1-91 in 8 min
<u>*Calculated Mass*</u>: 11253 Da (Halide ligand to Pd is lost on ionization)
<u>*Observed Mass*</u>: 11253 Da

2.16. Max-Max Conjugation

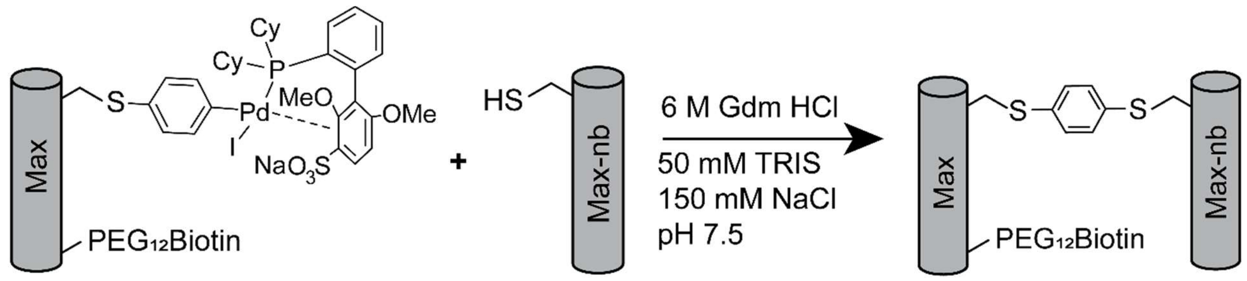

Synthesis of Max-Max. The protocol for protein dimerization was adapted from an earlier report.[5] To a 2.0 mL low-protein binding Eppendorf tube was added 1 equiv of the Max-Pd complex as a lyophilized powder. Separately, to a 2.0 mL low-protein binding Eppendorf tube was added 1 equiv of the Max-nb as a lyophilized powder. Sufficient denaturing buffer (6 M Gdm HCl, 50 mM TRIS HCl, 150 mM NaCl, pH 7.5) was added to the Eppendorf tube containing the Max-nb to produce a solution at 10 mg/mL (1.03 mM). The solution containing the Max-nb was quickly transferred to the Eppendorf tube containing the Max-Pd complex. The now-empty Eppendorf rube was rinsed with 15 μL of denaturing buffer, and the solution added to the reaction mixture in the other Eppendorf tube. After incubation for 30 min, the Eppendorf tube containing the reaction mixture was clarified by centrifugation at 21,000 × g for 10 min, and the supernatant purified with Method 1.4.3.

2.16.1. L-Max-Max-nb Dimerization Results

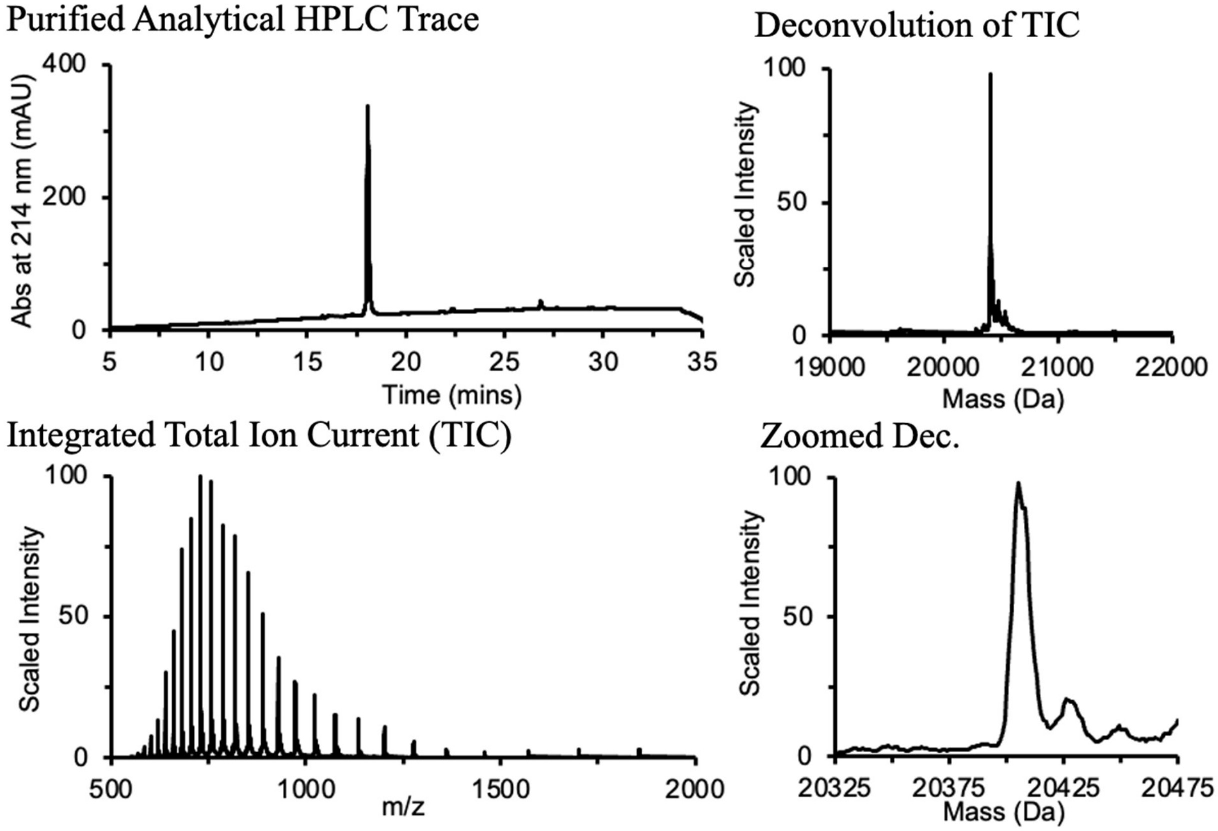

Starting Mass	Purified Mass	Isolated after Purification
2.2 mg	100% of reaction mix	1.2 mg
100% of iso L-Max-OAC		29.5% Yield

<u>*Purification Method*</u>: Method 1.4.3 - 1-Stage Semiprep
<u>*Purified HPLC Method*</u>: Method 1.2.3 - 5% to 65% in 30 min on Agilent 1290
<u>*Purified LCMS Method*</u>: Method 1.3.1 - 6545 1-91 in 8 min
<u>*Calculated Mass*</u>: 20406 Da
<u>*Observed Mass*</u>: 20406 Da

2.16.2. D-Max-Max-nb Dimerization Results

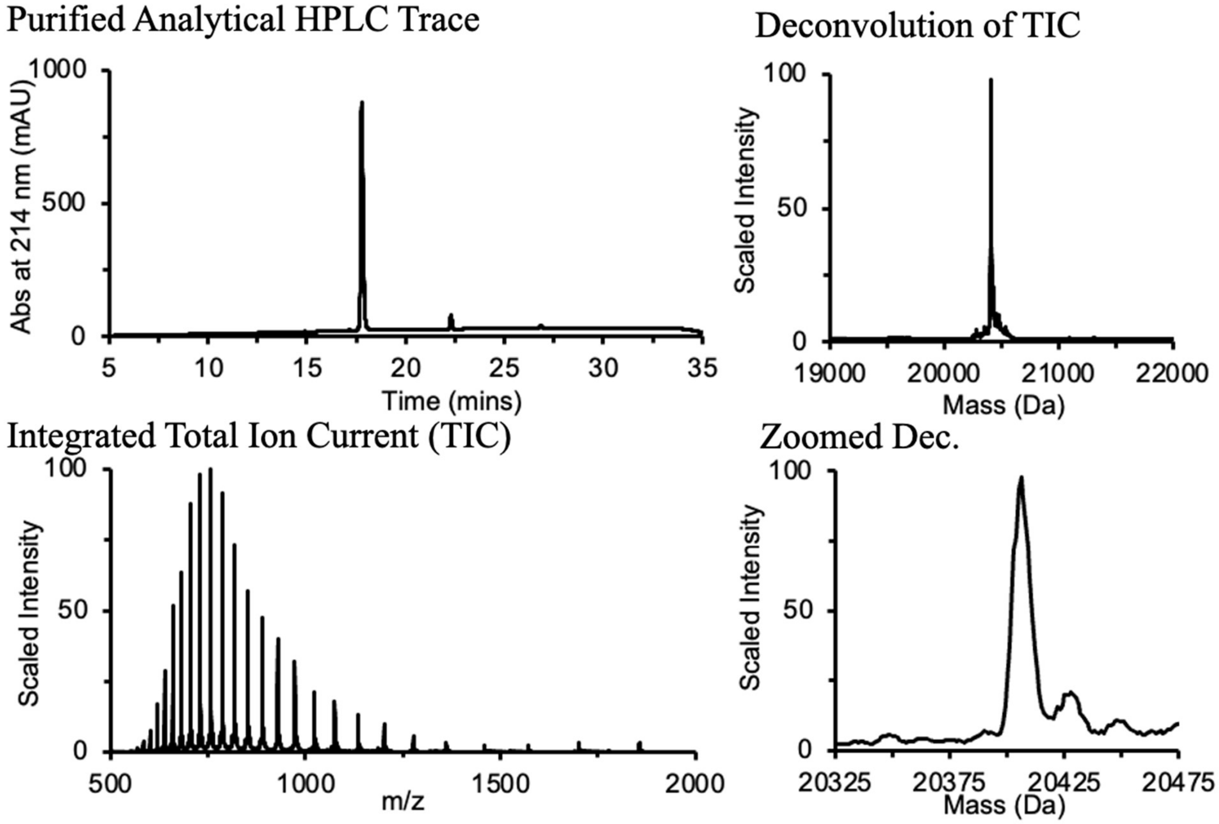

Starting Mass	Purified Mass	Isolated after Purification
1.1 mg	100% of reaction mix	0.7 mg
100% of iso D-Max-OAC		34.4% Yield

Purification Method: Method 1.4.3 - 1-Stage Semiprep
Purified HPLC Method: Method 1.2.3 - 5% to 65% in 30 min on Agilent 1290
Purified LCMS Method: Method 1.3.1 - 6545 1-91 in 8 min
Calculated Mass: 20406 Da
Observed Mass: 20406 Da

2.17. Myc from AFPS

Protein	Uniprot ID	Start	End	Length	Modifications
Myc	P01106	353	427	85	N-Term PEG_{12}Biotin, C-Term +GGC + carboxamide
NVKRRTHNVL ERQRRNELKR SFFALRDQIP ELENNEKAPK VVILKKATAY ILSVQAEEQK LISEEDLLRK RREQLKHKLE QLGGC					

2.17.1. L-Myc AFPS Results

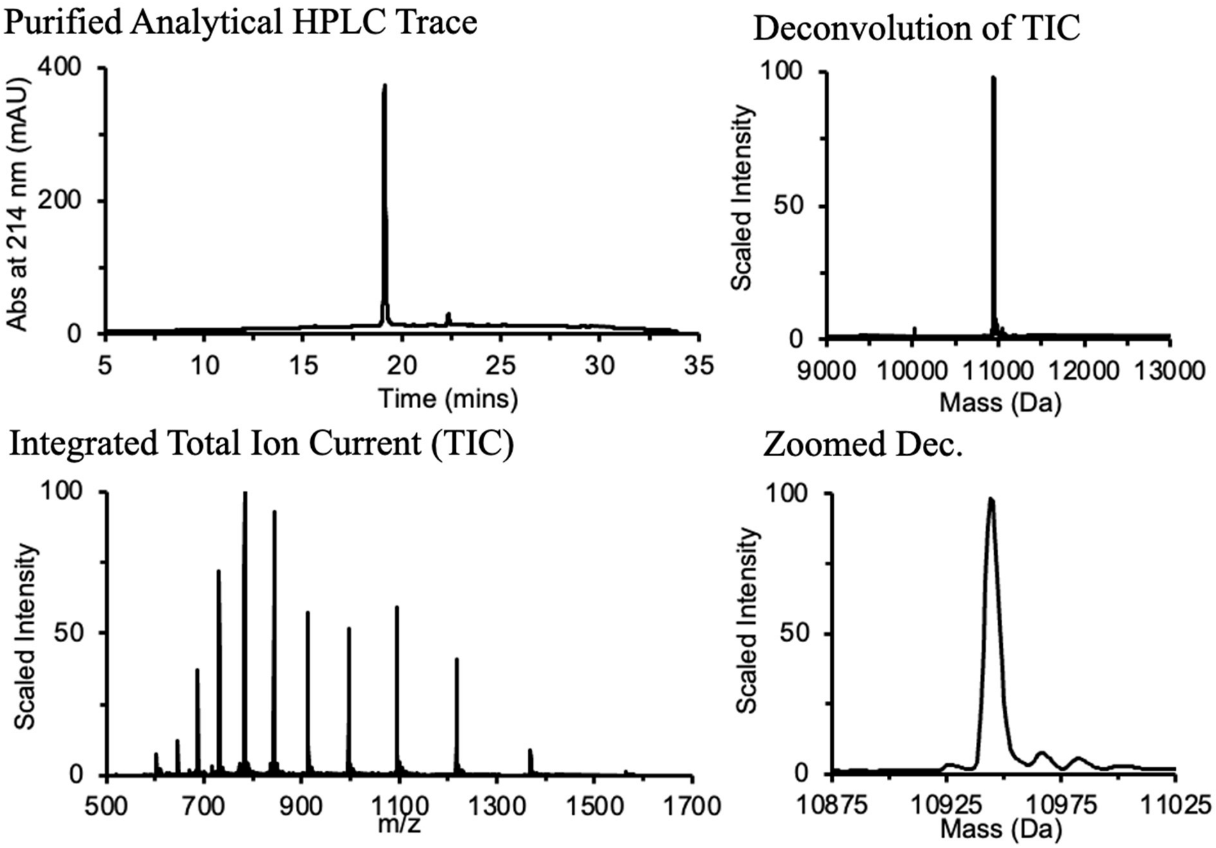

Starting Resin	Crude Resin	Cleaved Resin	Isolated Mass	Theoretical Mass	Purified Mass	Isolated after Purification	Extrapolated Yield
85 mg	n.d.	n.d.	163.7 mg	560 mg	163.7 mg	14.4 mg	14.4
0.49 mmol / g (41.6 μmol)	100% of total resin		29% Yield		100% of Isolated Peptide	9% Purification Yield	2.6% Overall Yield

Cleavage Method: Method 1.1.2
Purification Method: Method 1.4.2 - 2 Stage Prep
Purified HPLC Method: Method 1.2.3 - 5% to 65% in 30 min on Agilent 1290
Purified LCMS Method: Method 1.3.3 - 6550-1 5-95 in 10 min
Calculated Mass: 10945 Da
Observed Mass: 10945 Da

2.17.2. D-Myc AFPS Results

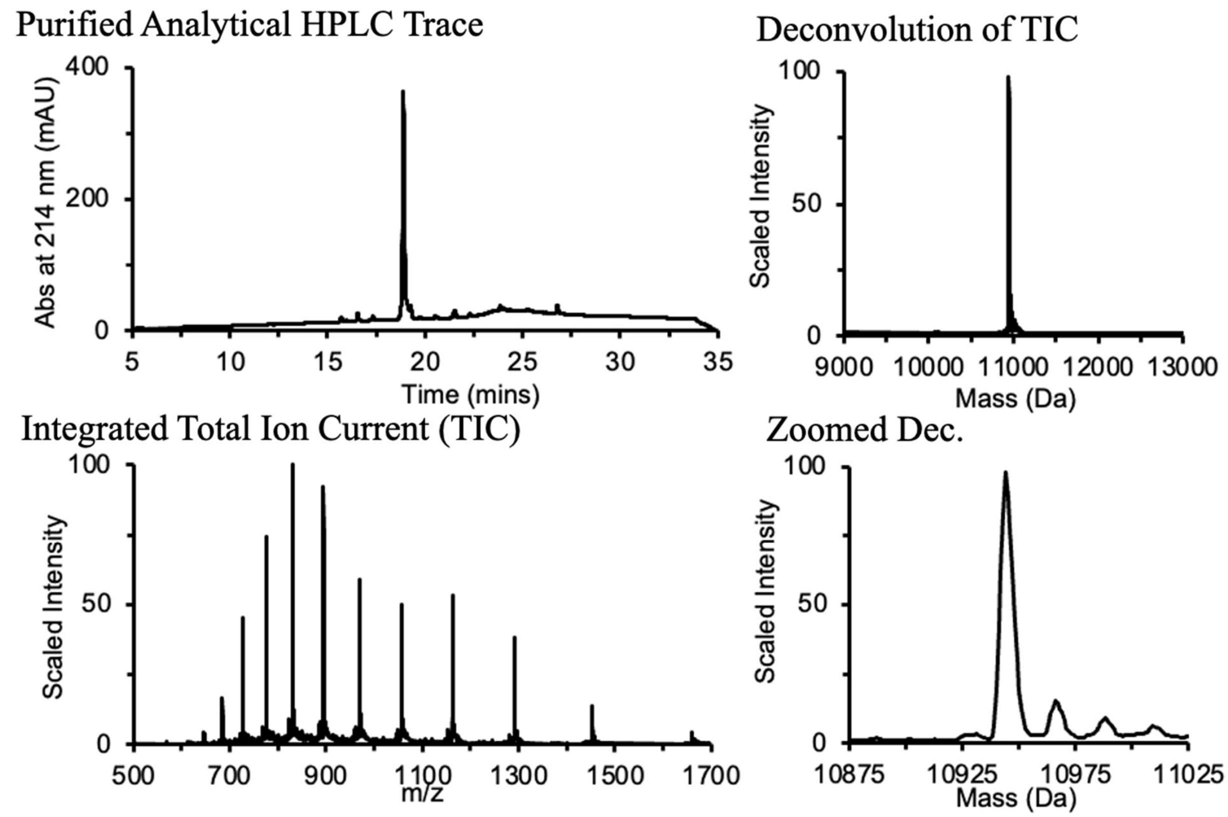

Starting Resin	Crude Resin	Cleaved Resin	Isolated Mass	Theoretical Mass	Purified Mass	Isolated after Purification	Extrapolated Yield
130 mg	n.d.	n.d.	93.8 mg	280.2 mg	93.8 mg	7.1 mg	14.2 mg
0.18 mmol / g (23.4 μmol)	50% of total resin		33% Yield		100% of Isolated Peptide	8% Purification Yield	2.5% Overall Yield

Cleavage Method: Method 1.1.1 - Reagent K
Purification Method: Method 1.4.1 - 2 Stage Semi-Prep
Purified HPLC Method: Method 1.2.3 - 5% to 65% in 30 min on Agilent 1290
Purified LCMS Method: Method 1.3.3 - 6550-1 5-95 in 10 min
Calculated Mass: 10945 Da
Observed Mass: 10945 Da

2.18. Myc Oxidative Addition Complex Synthesis

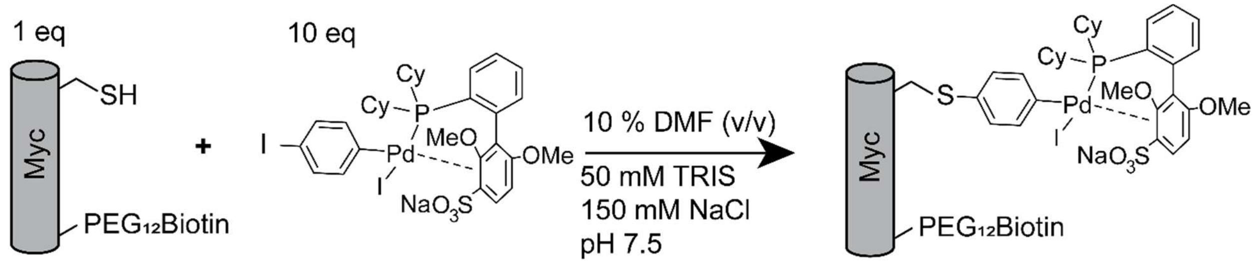

Synthesis of Myc-OAC. The protocol for OAC generation was adapted from an earlier report.[5] To a 50 mL Falcon tube was added the purified Myc as a lyophilized powder and sufficient reaction buffer (50 mM TRIS HCl, 150 mM NaCl, pH 7.5) was added to prepare a solution at 2 mg/mL (205 μM). Protein concentration was measured with Method 1.6.1, and the concentration of the protein stock adjusted to 1.1 mg/mL (113 μM) with addition reaction buffer. Separately, to a 2.0 mL Eppendorf tube was added 10 eq of sSPhos reinsertion reagent relative to the protein and dissolved in DMF to 4.8 mg/mL (6 mM). The sSPhos reinsertion reagent in DMF was added to the protein stock, the Falcon tube quickly recapped, and the apparatus vortexed. After incubation for 5 min, the Falcon tube screw cap was removed, and the mixture was transferred to a 20 mL syringe affixed with a 0.22 μM nylon syringe filter. The mixture was forced through the syringe filter into a fresh Falcon tube. The Falcon tube originally containing the un-filtered peptide solution was rinsed with an additional 2 mL of the reaction buffer that was then filtered using the same filtration apparatus into the Falcon tube containing the filtered peptide solution. Any bubbles generated were removed by centrifugation at 3220 × g. The peptide mixture was then purified according to Method 1.4.3.

2.18.1. L-Myc Oxidative Addition Complex Results

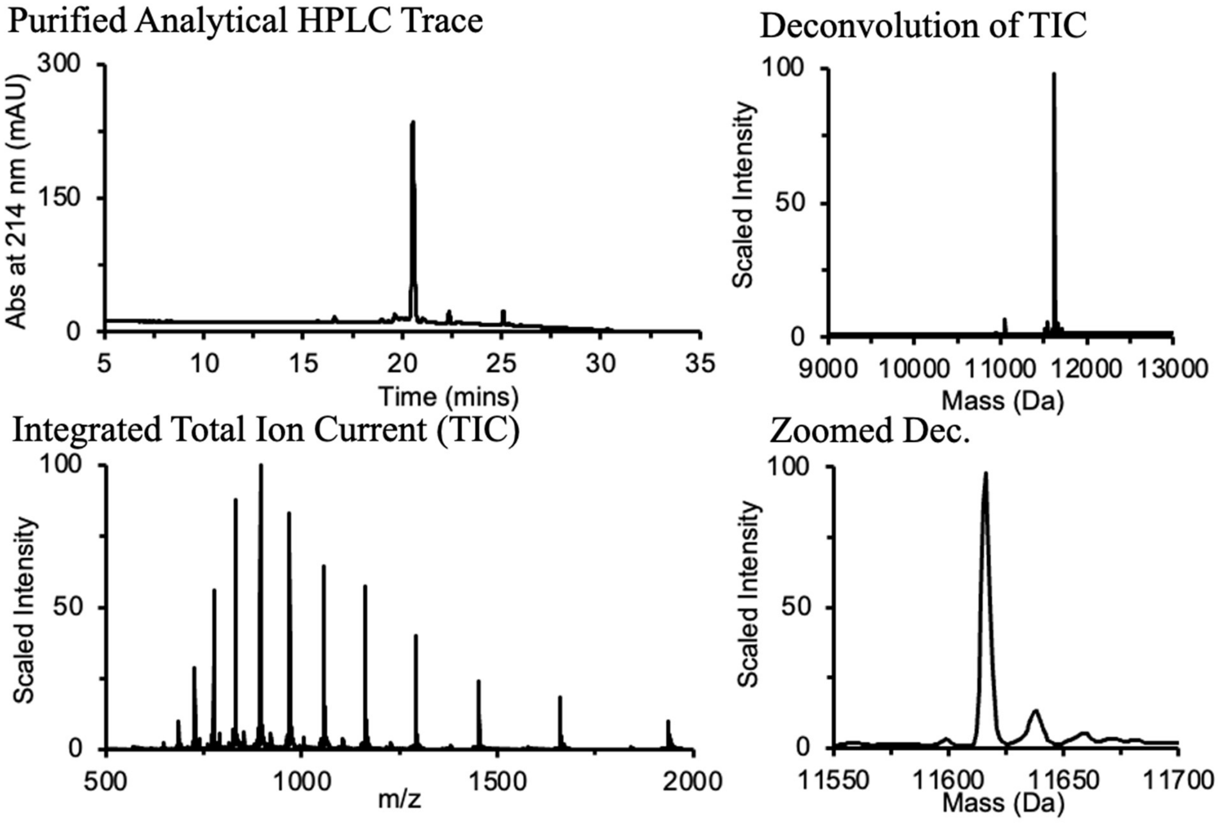

Starting Mass	Purified Mass	Isolated after Purification
10.0 mg	100% of reaction mix	1.2 mg
69% of iso L-Myc		11.5% Yield

<u>*Purification Method*</u>: Method 1.4.3 - 1-Stage Semiprep
<u>*Purified HPLC Method*</u>: Method 1.2.3 - 5% to 65% in 30 min on Agilent 1290
<u>*Purified LCMS Method*</u>: Method 1.3.1 - 6545 1-91 in 8 min
<u>*Calculated Mass*</u>: 11618 Da (Halide ligand to Pd is lost on ionization)
<u>*Observed Mass*</u>: 11618 Da

2.18.2. D-Myc Oxidative Addition Complex Results

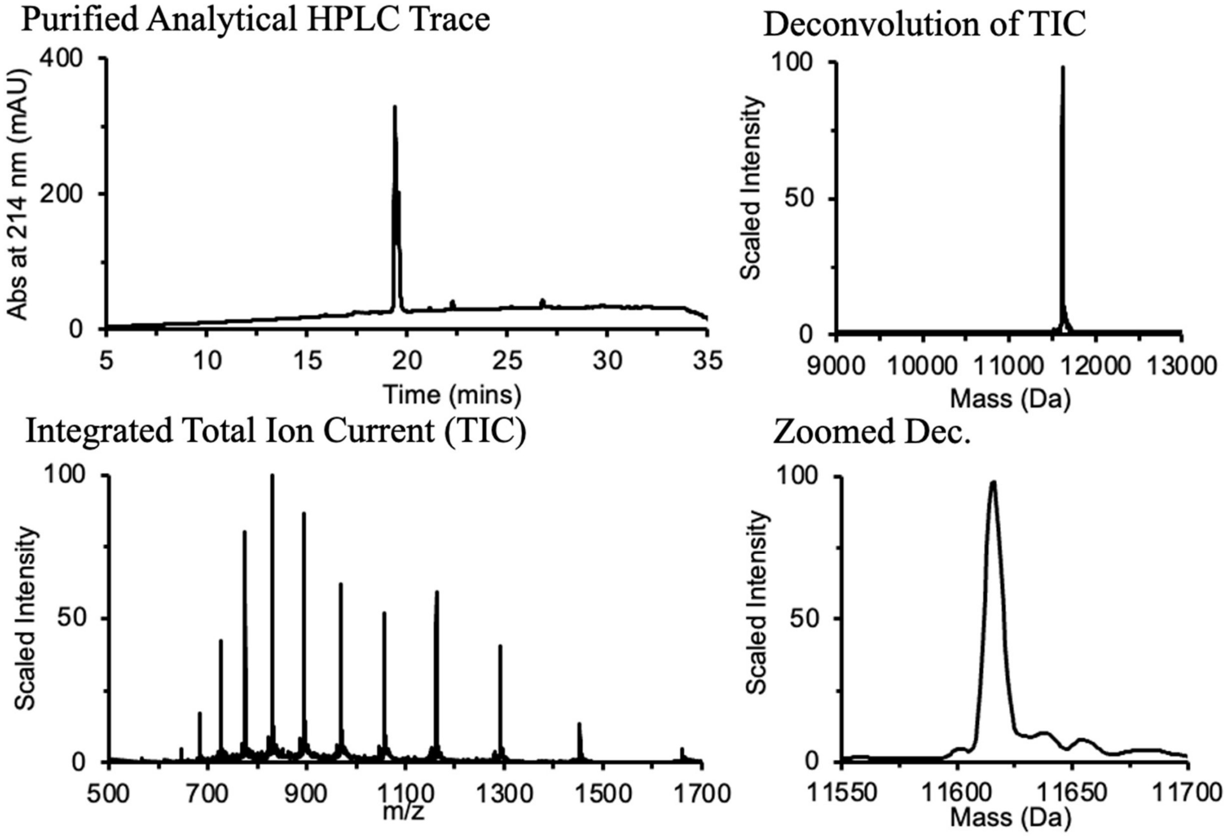

Starting Mass	Purified Mass	Isolated after Purification
7.1 mg	100% of reaction mix	2.1 mg
100% of iso D-Myc		28.2% Yield

<u>Purification Method</u>: Method 1.4.3 - 1-Stage Semiprep
<u>Purified HPLC Method</u>: Method 1.2.3 - 5% to 65% in 30 min on Agilent 1290
<u>Purified LCMS Method</u>: Method 1.3.3 - 6550-1 5-95 in 10 min
<u>Calculated Mass</u>: 11618 Da (Halide ligand to Pd is lost on ionization)
<u>Observed Mass</u>: 11618 Da

2.19. Myc-Max Conjugation

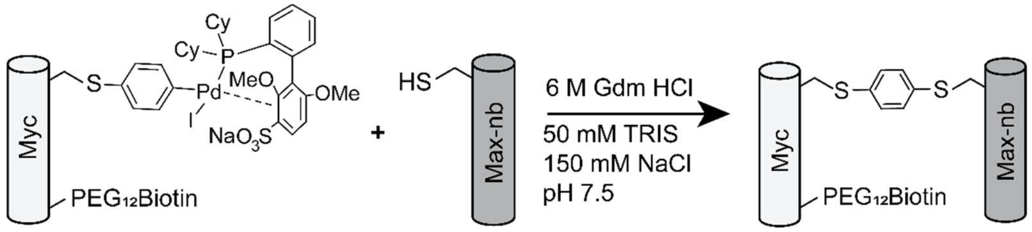

Synthesis of Myc-Max. The protocol for protein dimerization was adapted from an earlier report.[5] To a 2.0 mL low-protein binding Eppendorf tube was added 1 equiv of the Myc-Pd complex as a lyophilized powder. Separately, to a 2.0 mL low-protein binding Eppendorf tube was added 1 equiv of the Max-nb as a lyophilized powder. Sufficient denaturing buffer (6 M Gdm HCl, 50 mM TRIS HCl, 150 mM NaCl, pH 7.5) was added to the Eppendorf tube containing the Max-nb to produce a solution at 10 mg/mL (1.03 mM). The solution containing the Max-nb was quickly transferred to the Eppendorf tube containing the Myc-Pd complex. The now-empty Eppendorf rube was rinsed with 15 μL of denaturing buffer, and the solution added to the reaction mixture in the other Eppendorf tube. After incubation for 30 min, the Eppendorf tube containing the reaction mixture was clarified by centrifugation at 21,000 × g for 10 min, and the supernatant purified with Method 1.4.3.

2.19.1. L-Myc-Max-nb Dimerization Results

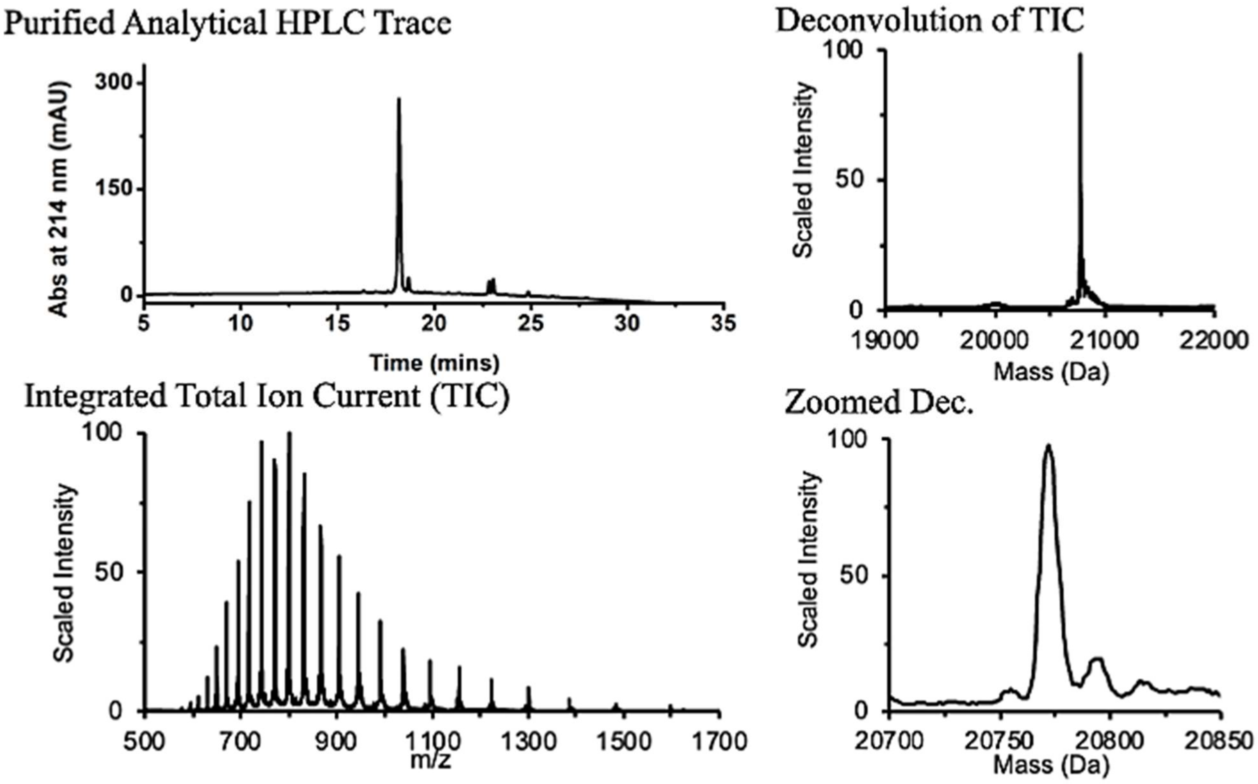

Starting Mass	Purified Mass	Isolated after Purification
1.2 mg	100% of reaction mix	0.6 mg
100% of iso L-Myc-OAC		27% Yield

<u>Purification Method</u>: Method 1.4.3 - 1-Stage Semiprep
<u>Purified HPLC Method</u>: Method 1.2.3 - 5% to 65% in 30 min on Agilent 1290
<u>Purified LCMS Method</u>: Method 1.3.3 - 6550-1 5-95 in 10 min
<u>Calculated Mass</u>: 20778 Da
<u>Observed Mass</u>: 20780 Da

2.19.2. D-Myc-Max-nb Dimerization Results

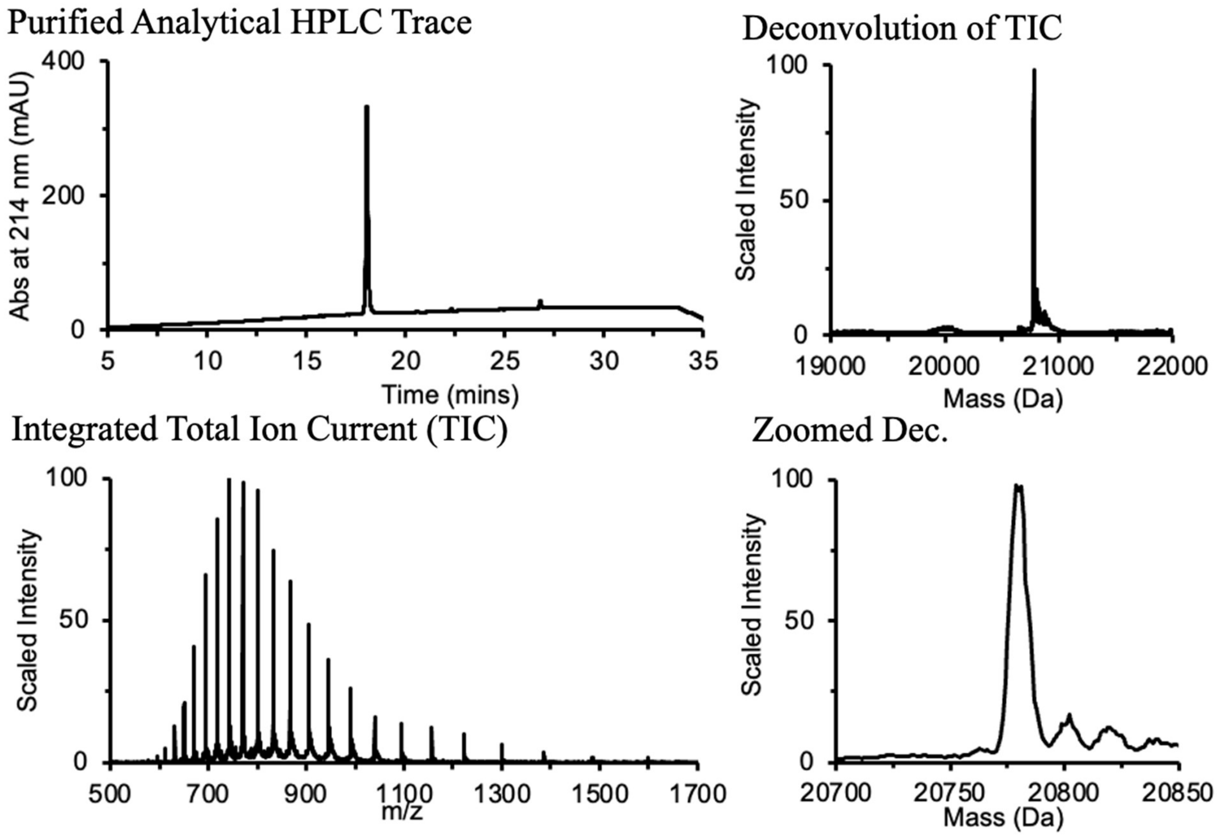

Starting Mass	Purified Mass	Isolated after Purification
2.1 mg	100% of reaction mix	1.3 mg
100% of iso D-Myc-OAC		33.8% Yield

Purification Method: Method 1.4.3 - 1-Stage Semiprep
Purified HPLC Method: Method 1.2.3 - 5% to 65% in 30 min on Agilent 1290
Purified LCMS Method: Method 1.3.3 - 6550-1 5-95 in 10 min
Calculated Mass: 20778 Da
Observed Mass: 20780 Da

2.20. MDM2 Folding Results

Synthetic MDM2 was folded in a similar manner to previous reports via dilution (see Methods).[1] After folding, the protein solution was transferred to an Amicon Ultra 15 mL 3K MWCO spin filtration unit, and the apparatus spun at 3220 × g until the volume of the retentate was approximately 1 mL. The concentrated protein solution was transferred to an Amicon Ultra 0.5 mL 3K MWCO spin filtration unit, and the apparatus spun at 14,000 × g until the volume of the retentate was approximately 100 μL. Separately, a semi-preparative SEC purification apparatus was prepared in the same manner as described in the **Methods** section and the concentrated protein solution injected in total. The peak corresponding to the major product was collected and concentrated to 100 μL protein with an Amicon Ultra 0.5 mL 3K MWCO spin filtration unit. The concentration of the solution was measured with ***Method 1.6.1***. The protein stock was separated into 1 nmol aliquots, flash frozen, and stored at −80 °C.

2.20.1. Dilution Folding of L-MDM2

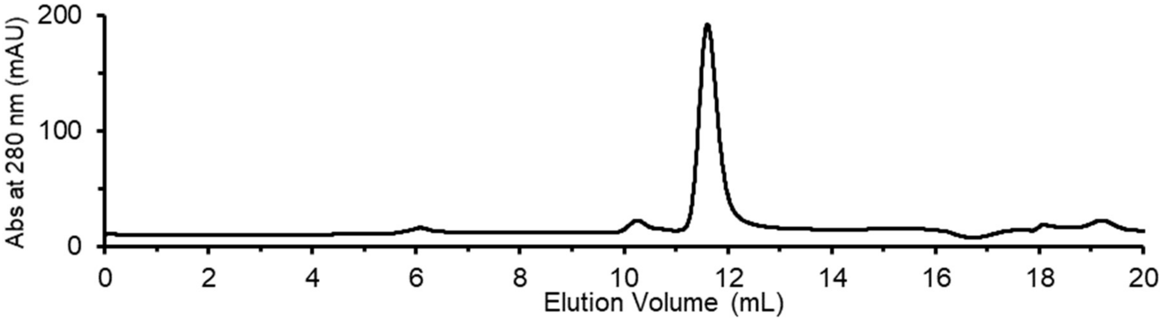

Starting Weight	Starting mol	Isolated Volume	Isolated [Protein] (mg/mL)	Isolated [Protein] (μM)	Isolated mol	Extrapolated Yield
1.2 mg	93.2 nmol	950 μL	0.11 mg / mL	10.3 μM	9.7 nmol	48.7 nmol
100% of Isolated Peptide	10.5% Folding Yield					

2.20.2. Dilution Folding of D-MDM2

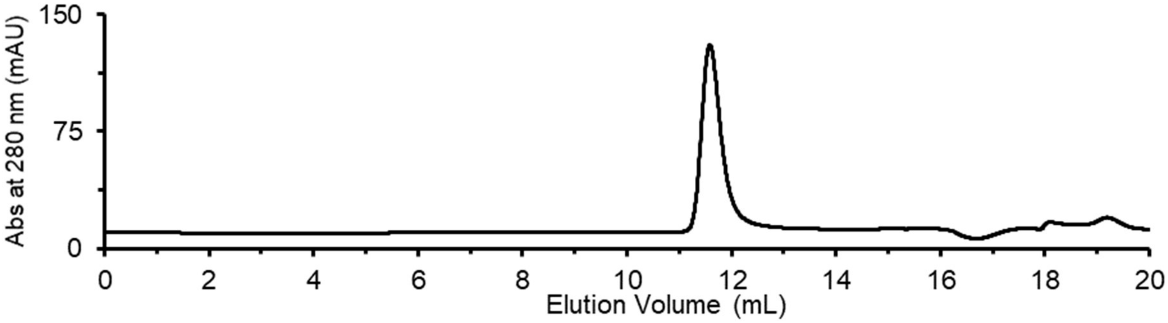

Starting Weight	Starting mol	Isolated Volume	Isolated [Protein] (mg/mL)	Isolated [Protein] (μM)	Isolated mol	Extrapolated Yield
0.6 mg	48 nmol	100 μL	0.49 mg / mL	45.5 μM	4.5 nmol	75.2 nmol
24% of Isolated Peptide	9.4% Folding Yield					

2.21. ERG Folding Results

Synthetic ERG was folded according to the protocol described in the Methods section, and the resulting folded protein concentration measured with ***Method 1.6.1***.

2.21.1. SEC Folding of L-ERG

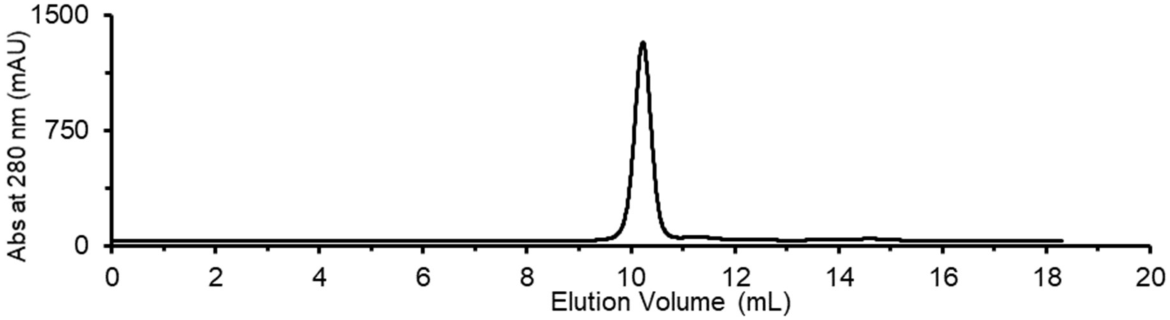

Starting Weight	Starting mol	Isolated Volume	Isolated [Protein] (mg/mL)	Isolated [Protein] (μM)	Isolated mol	Extrapolated Yield
1.0 mg	74.0 nmol	938 μL	0.4 mg / mL	30.6 μM	28.7 nmol	361.5 nmol
15.9% of Isolated Peptide	38.8% Folding Yield					

2.21.2. SEC Folding of D-ERG

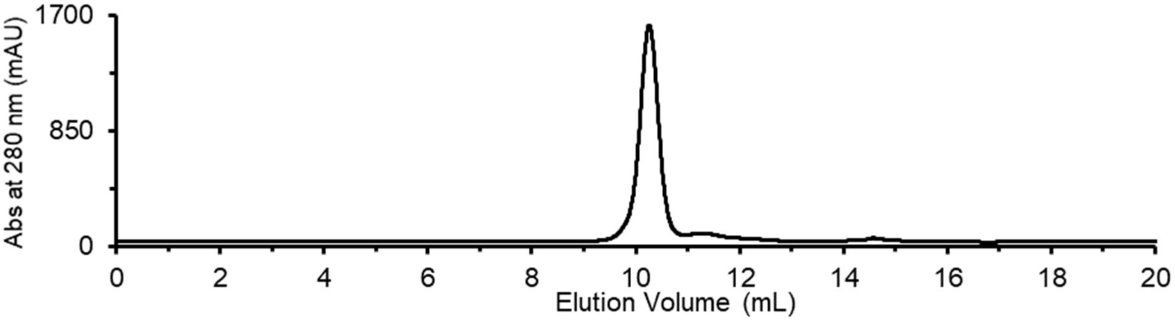

Starting Weight	Starting mol	Isolated Volume	Isolated [Protein] (mg/mL)	Isolated [Protein] (μM)	Isolated mol	Extrapolated Yield
1.0 mg	74.0 nmol	839 μL	0.5 mg / mL	44.9 μM	37.6 nmol	521.5 nmol
14.4% of Isolated Peptide	50.9% Folding Yield					

2.22. Barnase Folding Results

Synthetic Barnase was folded according to the protocol described in the Methods section, and the major peak corresponding to the major product was collected and concentrated to 100 μL protein with an Amicon Ultra 0.5 mL 3K MWCO spin filtration unit. The concentration was measured with ***Method 1.6.1***. The protein stock was separated into 1 nmol aliquots, flash frozen, and stored at −80 °C.

2.22.1. SEC Folding of L-Barnase

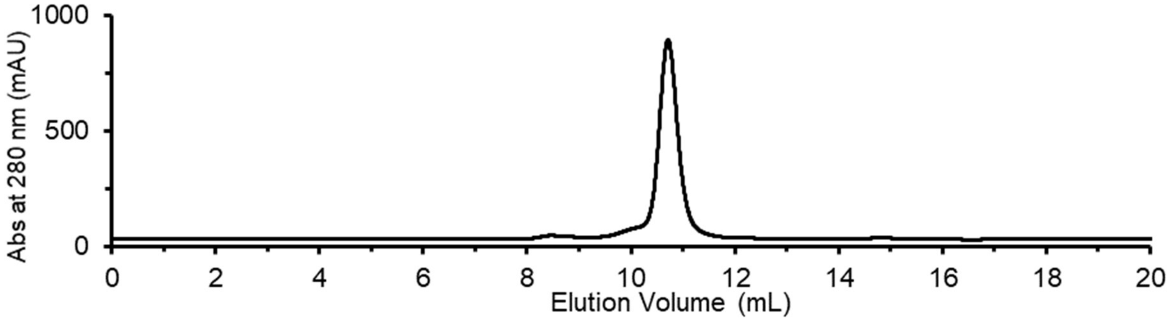

Starting Weight	Starting mol	Isolated Volume	Isolated [Protein] (mg/mL)	Isolated [Protein] (μM)	Isolated mol	Extrapolated Yield
0.6 mg	39.9 nmol	100 μL	0.8 mg / mL	58.2 μM	5.8 nmol	32.2 nmol
37.5% of Isolated Peptide	14.6% Folding Yield					

2.22.2. SEC Folding of D-Barnase

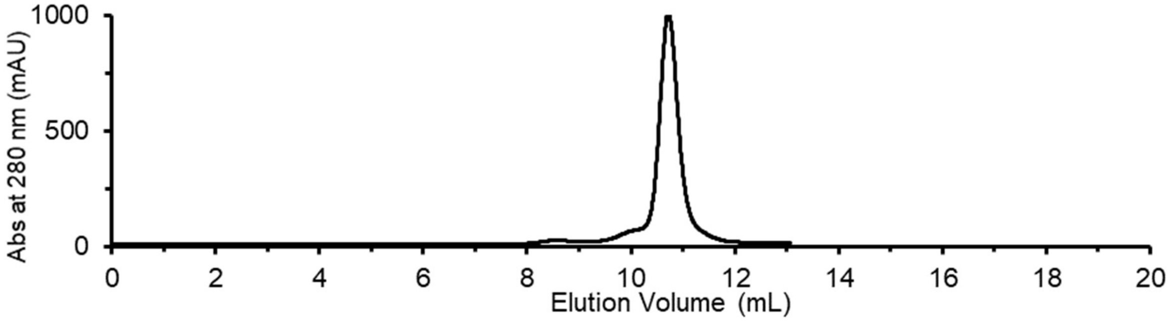

Starting Weight	Starting mol	Isolated Volume	Isolated [Protein] (mg/mL)	Isolated [Protein] (μM)	Isolated mol	Extrapolated Yield
0.6 mg	39.9 nmol	100 μL	1.4 mg / mL	105.9 μM	10.6 nmol	81.8 nmol
26.1% of Isolated Peptide	26.5% Folding Yield					

2.23. IRAK2 Folding Results

Synthetic IRAK2 was folded according to the protocol described in the Methods section, and the resulting folded protein concentration measured with ***Method 1.6.1***.

2.23.1. SEC Folding of L-IRAK2

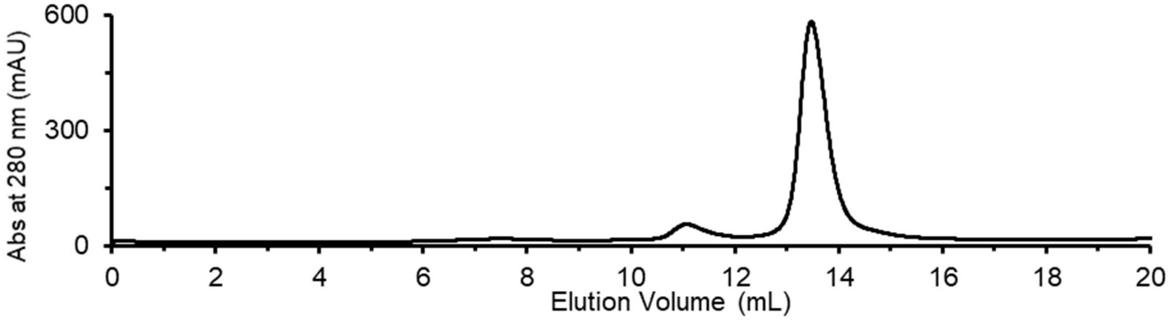

Starting Weight	Starting mol	Isolated Volume	Isolated [Protein] (mg/mL)	Isolated [Protein] (μM)	Isolated mol	Extrapolated Yield
0.6 mg	39.1 nmol	838 μL	0.1 mg / mL	8.4 μM	7.0 nmol	48.2 nmol
29.3% of Isolated Peptide	17.9% Folding Yield					

As there was a larger molecular weight shoulder that was removed upon the SEC purification, additional analytical SEC of the purified SEC fraction was recorded to verify its removal. The SEC purification performed as described in the Methods section, with minor modifications: the injected sample was 1 nmol of folded L-IRAK2 not denatured, the chromatogram was recorded at 214 nm for increased sensitivity, and the running buffer was 50 mM TRIS, 150 mM NaCl, 5% Glycerol (v/v), pH 7.5.

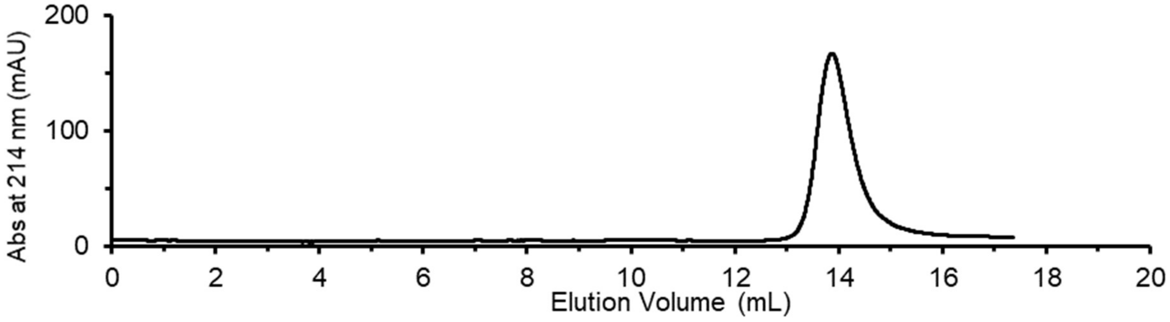

2.23.2. SEC Folding of D-IRAK2

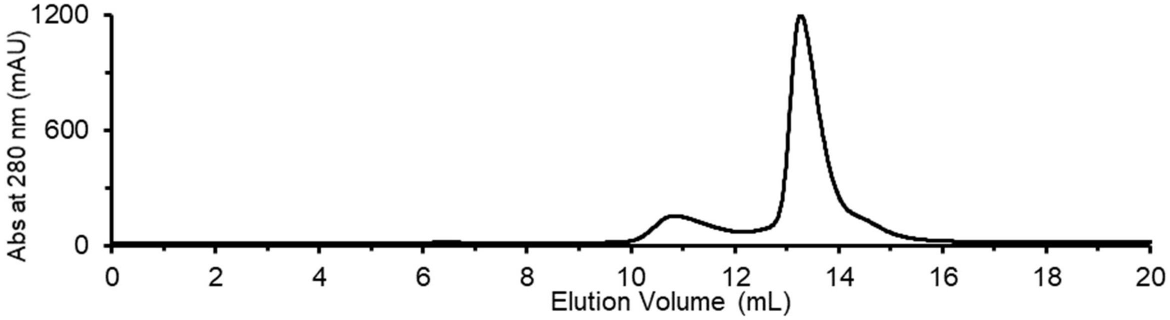

Starting Weight	Starting mol	Isolated Volume	Isolated [Protein] (mg/mL)	Isolated [Protein] (μM)	Isolated mol	Extrapolated Yield
0.6 mg	39.1 nmol	1.15 mL	0.2 mg / mL	15.2 μM	17.4 nmol	66.6 nmol
100% of Isolated Peptide	44.6% Folding Yield					

As there was a larger molecular weight shoulder that was removed upon the SEC purification, additional analytical SEC of the purified SEC fraction was recorded to verify its removal. The SEC purification performed as described in the Methods section, with minor modifications: the injected sample was 1 nmol of folded D-IRAK2 not denatured, the chromatogram was recorded at 214 nm for increased sensitivity, and the running buffer was 50 mM TRIS, 150 mM NaCl, 5% Glycerol (v/v), pH 7.5.

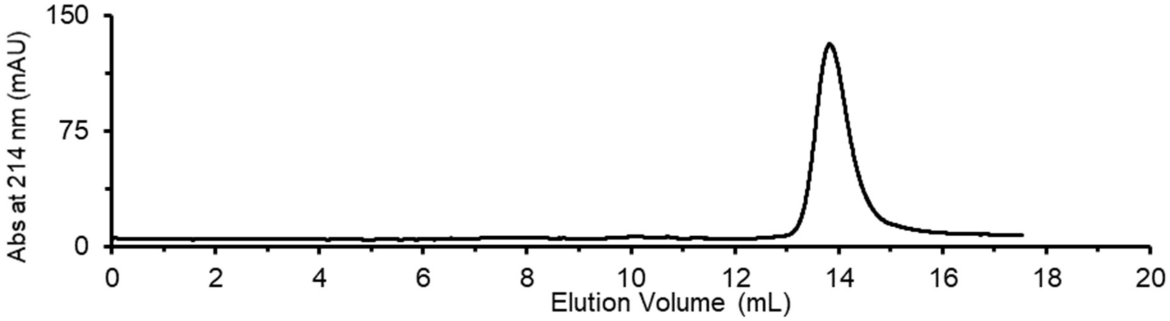

2.24. CHIP Folding Results

Synthetic CHIP was folded according to the protocol described in the Methods section, and the resulting folded protein concentration measured with ***Method 1.6.1***.

2.24.1. SEC Folding of L-CHIP

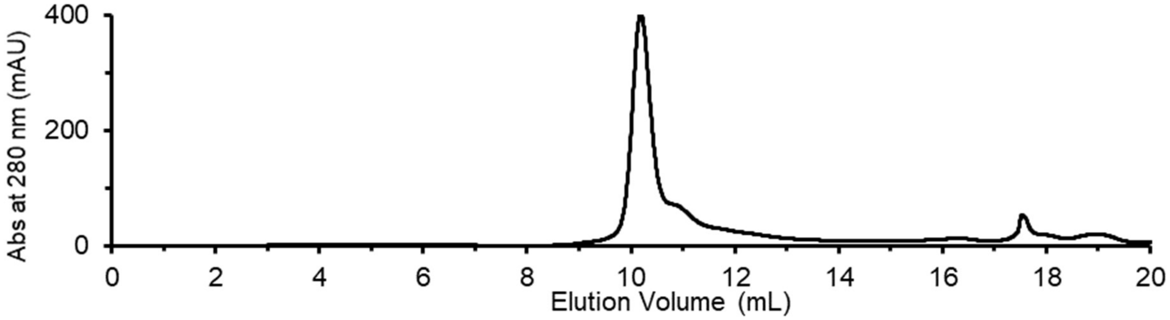

Starting Weight	Starting mol	Isolated Volume	Isolated [Protein] (mg/mL)	Isolated [Protein] (μM)	Isolated mol	Extrapolated Yield
0.6 mg	32.2 nmol	1012 μL	0.2 mg / mL	14.2 μM	14.3 nmol	44.0 nmol
100% of Isolated Peptide	44.6% Folding Yield					

2.24.2. SEC Folding of D-CHIP

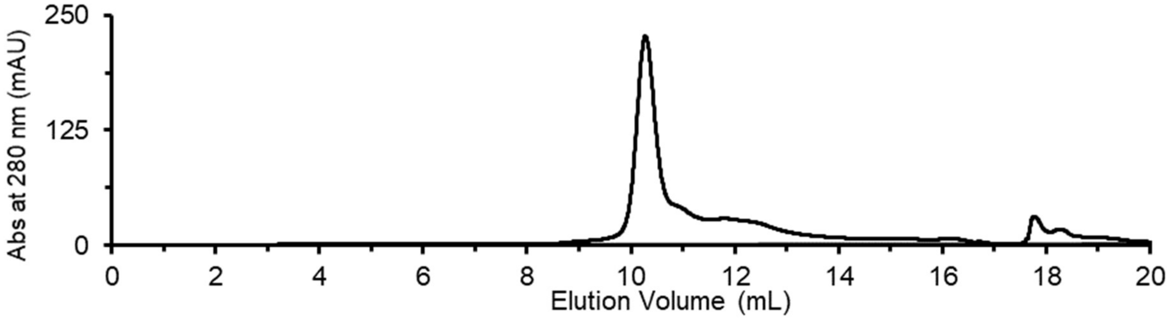

Starting Weight	Starting mol	Isolated Volume	Isolated [Protein] (mg/mL)	Isolated [Protein] (μM)	Isolated mol	Extrapolated Yield
1.6 mg	85.8 nmol	896 μL	0.4 mg / mL	23.5 μM	21.0 nmol	21.0 nmol
100% of Isolated Peptide	24.5% Folding Yield					

2.25. NEMO Folding Results

Synthetic NEMO was folded according to the protocol described in the Methods section, and the resulting folded protein concentration measured with ***Method 1.6.2***. Because of NEMO's low extinction coefficient at 280 nm, the SEC chromatogram was monitored at 230 nm.

2.25.1. SEC Folding of L-NEMO

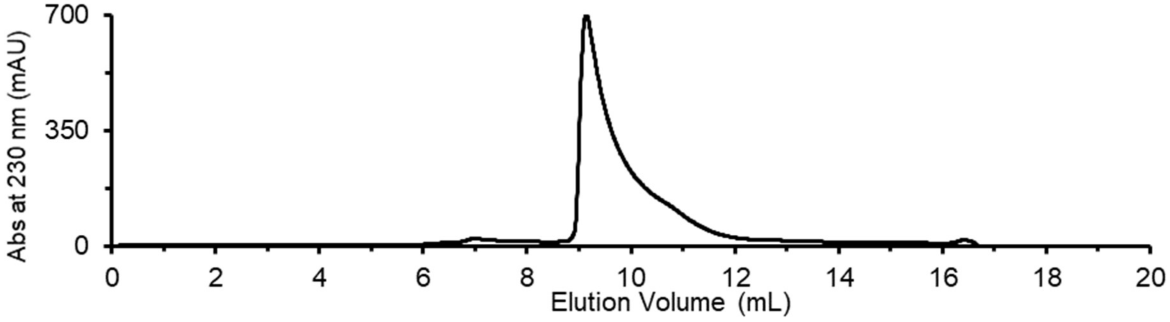

Starting Weight	Starting mol	Isolated Volume	Isolated [Protein] (mg/mL)	Isolated [Protein] (μM)	Isolated mol	Extrapolated Yield
1.6 mg	149.0 nmol	633 μL	0.4 mg / mL	41.5 μM	26.3 nmol	92.6 nmol
100.0% of Isolated Peptide	17.6% Folding Yield					

2.25.2. SEC Folding of D-NEMO

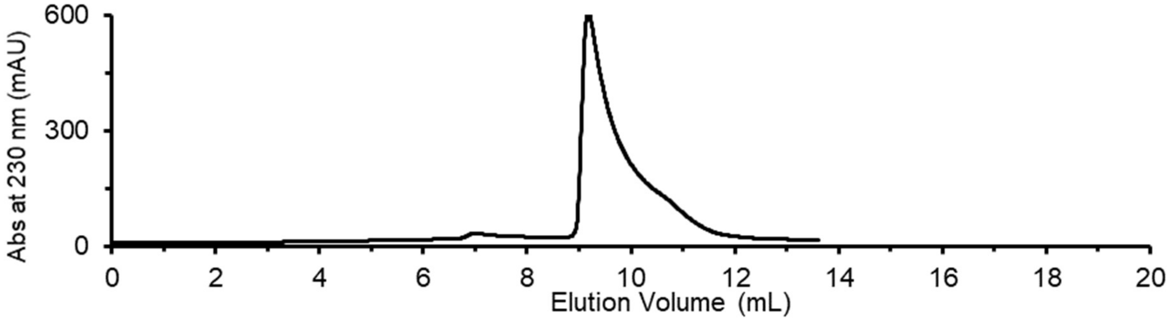

Starting Weight	Starting mol	Isolated Volume	Isolated [Protein] (mg/mL)	Isolated [Protein] (μM)	Isolated mol	Extrapolated Yield
2.5 mg	232 nmol	1200 μL	0.3 mg / mL	30.7 μM	36.8 nmol	191.5 nmol
38.5% of Isolated Peptide	15.8% Folding Yield					

2.26. FKBP12 Folding Results

Synthetic FKBP12 was folded according to the protocol described in the Methods section, and the resulting folded protein concentration measured with ***Method 1.6.1***.

*2.26.1. SEC Folding of L-FKBP12**

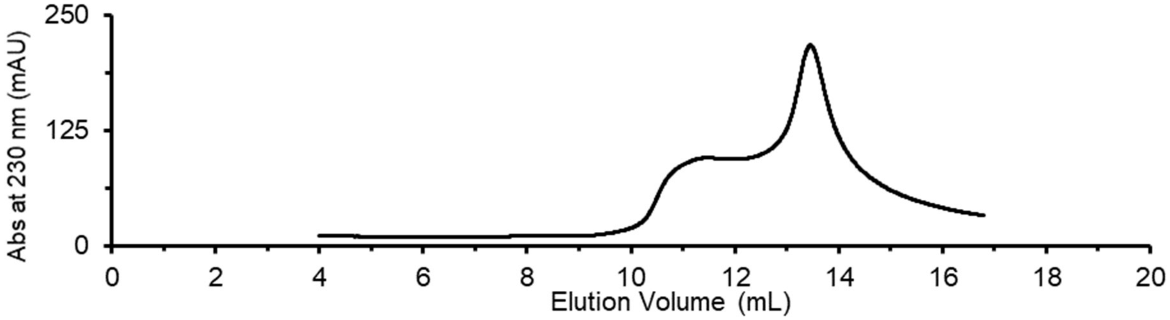

Starting Weight	Starting mol	Isolated Volume	Isolated [Protein] (mg/mL)	Isolated [Protein] (μM)	Isolated mol	Extrapolated Yield
2.4 mg	165.2 nmol	1000 μL	0.3 mg / mL	20.1 μM	20.1 nmol	62.6 nmol
100% of Isolated Peptide	12.2% Folding Yield					

*An error occurred during initiation of the UV recorder, and data was only collected an unknown amount of time after the run had started. As a result, the absolute elution time of the above peak is unknown. An additional buffer of 4 mL is added to the beginning of the run time to account for the recorder error. This value of 4 mL was estimated as the offset to account for the time required to restart the analysis software, and results in a peak elution time that is in good agreement with the subsequent analytical SEC trace.

As there was a larger molecular weight shoulder that was removed upon the SEC purification, additional analytical SEC of the purified SEC fraction was recorded to verify its removal. The SEC purification performed as described in the Methods section, with minor modifications: the injected sample was 1 nmol of folded L-FKBP12 not denatured, the chromatogram was recorded at 214 nm for increased sensitivity, and the running buffer was 50 mM TRIS, 150 mM NaCl, 5% Glycerol (v/v), pH 7.5.

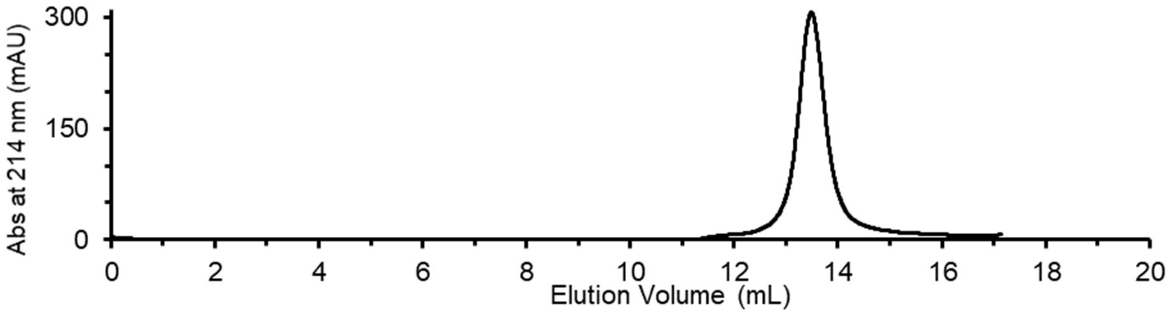

2.26.2. SEC Folding of D-FKBP12

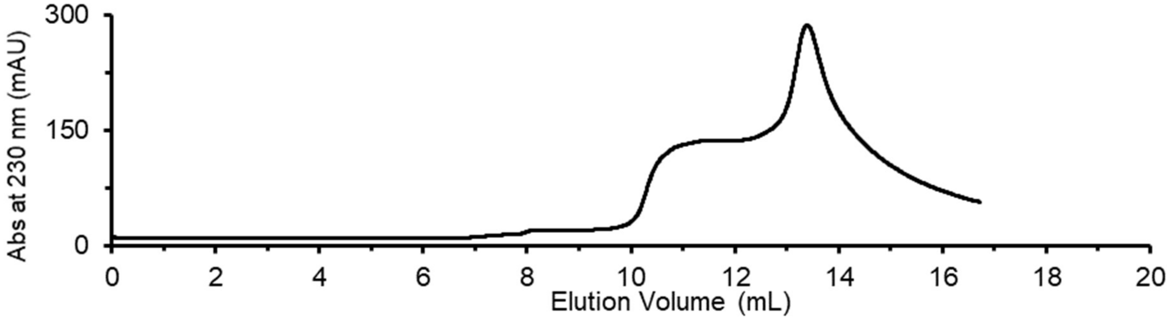

Starting Weight	Starting mol	Isolated Volume	Isolated [Protein] (mg/mL)	Isolated [Protein] (μM)	Isolated mol	Extrapolated Yield
2.2 mg	151.4 nmol	870 μL	0.4 mg / mL	29.2 μM	25.4 nmol	63.6 nmol
100% of Isolated Peptide	16.8% Folding Yield					

As there was a larger molecular weight shoulder that was removed upon the SEC purification, additional analytical SEC of the purified SEC fraction was recorded to verify its removal. The SEC purification performed as described in the Methods section, with minor modifications: the injected sample was 1 nmol of folded D-FKBP12 not denatured, the chromatogram was recorded at 214 nm for increased sensitivity, and the running buffer was 50 mM TRIS, 150 mM NaCl, 5% Glycerol (v/v), pH 7.5.

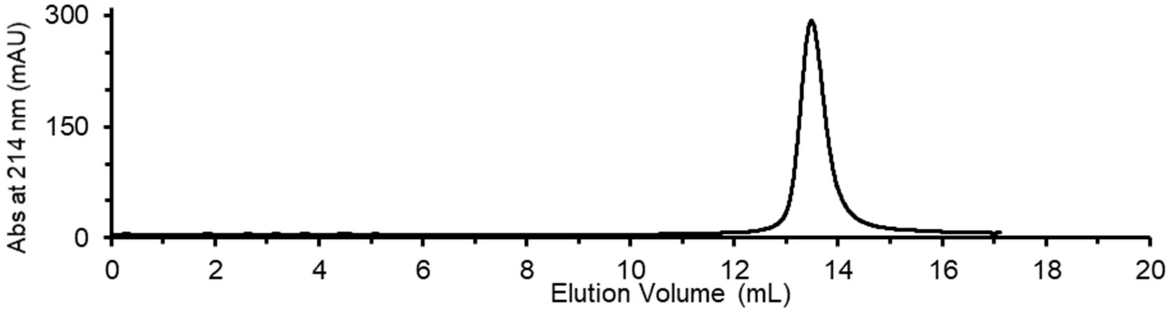

2.27. BCL11a Folding Results

Synthetic BCL11a was folded according to the protocol described in the Methods section, and the resulting folded protein concentration measured with ***Method 1.6.1***. The denaturing buffer and running buffer were adjusted to include $ZnCl_2$. Denaturing buffer: 8 M urea, 100 mM TRIS HCl, 150 mM NaCl, 5 mM $ZnCl_2$, pH 7.5 Running Buffer: 100 mM TRIS HCl, 150 mM NaCl, 0.5 mM $ZnCl_2$, pH 7.5.

2.27.1. SEC Folding of L-BCL11a

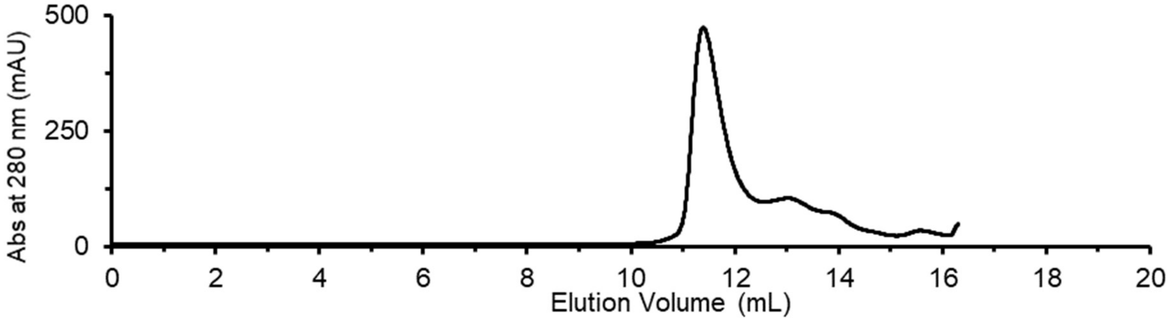

Starting Weight	Starting mol	Isolated Volume	Isolated [Protein] (mg/mL)	Isolated [Protein] (μM)	Isolated mol	Extrapolated Yield
2.5 mg	169.4 nmol	782 μL	0.7 mg / mL	55.3 μM	43.3 nmol	180.1 nmol
48.1% of Isolated Peptide	25.5% Folding Yield					

As there was a lower molecular weight shoulder that was removed upon the SEC purification, additional analytical SEC of the purified SEC fraction was recorded to verify its removal. The SEC purification performed as described in the Methods section, with minor modifications: the injected sample was 1 nmol of folded L-BCL11a not denatured, the chromatogram was recorded at 214 nm for increased sensitivity, and the running buffer was 100 mM TRIS, 150 mM NaCl, 0.5 mM $ZnCl_2$, pH 7.5.

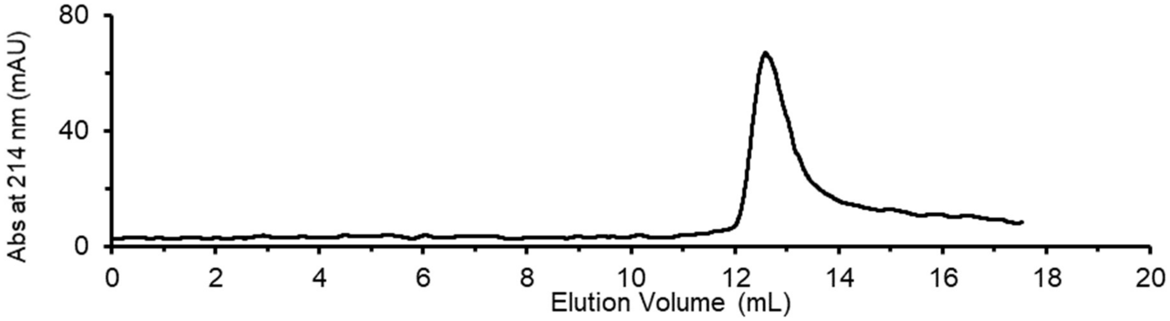

2.27.2. SEC Folding of D-BCL11a

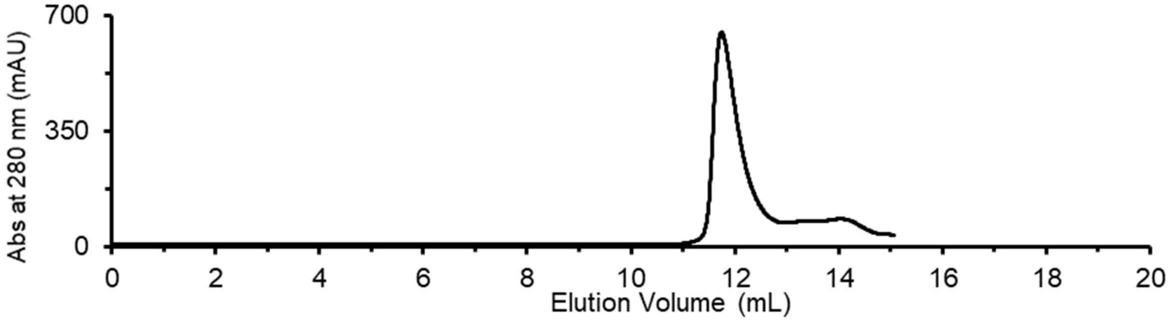

Starting Weight	Starting mol	Isolated Volume	Isolated [Protein] (mg/mL)	Isolated [Protein] (μM)	Isolated mol	Extrapolated Yield
2.4 mg	162.6 nmol	785 μL	0.4 mg / mL	36.4 μM	28.6 nmol	66.7 nmol
42.9% of Isolated Peptide	17.6% Folding Yield					

As there was a lower molecular weight shoulder that was removed upon the SEC purification, additional analytical SEC of the purified SEC fraction was recorded to verify its removal. The SEC purification performed as described in the Methods section, with minor modifications: the injected sample was 1 nmol of folded D-BCL11a not denatured, the chromatogram was recorded at 214 nm for increased sensitivity, and the running buffer was 100 mM TRIS, 150 mM NaCl, 0.5 mM $ZnCl_2$, pH 7.5.

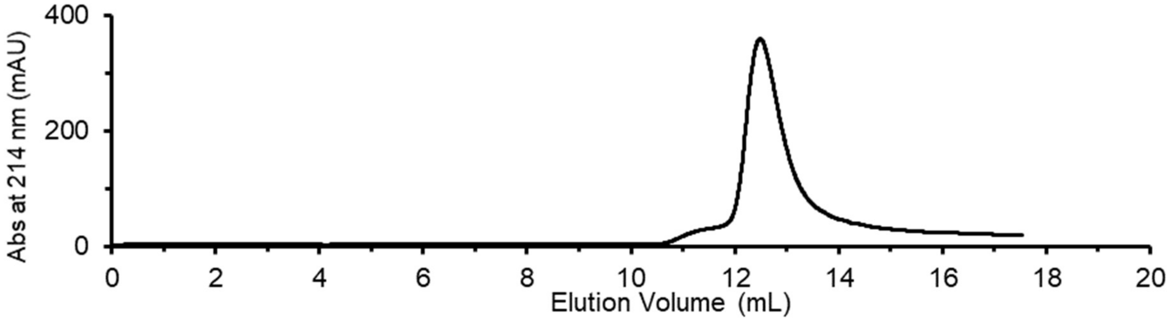

2.28. YAP1 Folding Results

Synthetic YAP1 was folded according to the protocol described in the Methods section, and the resulting folded protein concentration measured with ***Method 1.6.1***.

2.28.1. SEC Folding of L-YAP1

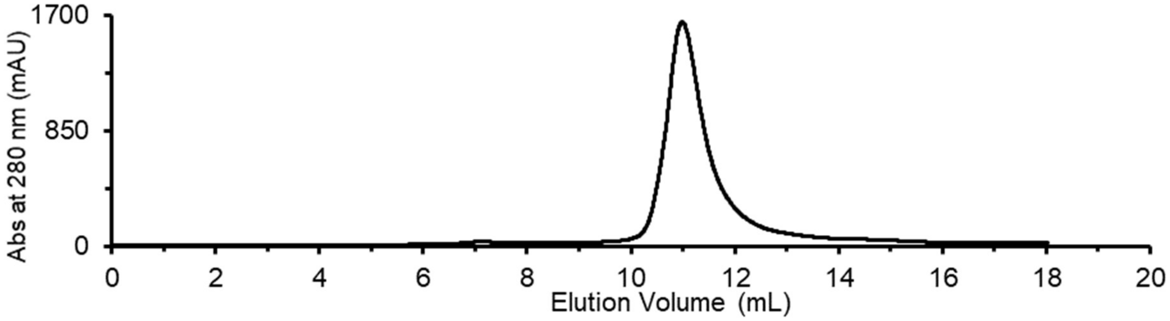

Starting Weight	Starting mol	Isolated Volume	Isolated [Protein] (mg/mL)	Isolated [Protein] (μM)	Isolated mol	Extrapolated Yield
1.1 mg	73.1 nmol	786 μL	0.7 mg / mL	54.0 μM	42.4 nmol	106.7 nmol
100% of Isolated Peptide	58.0% Folding Yield					

2.28.2. SEC Folding of D-YAP1

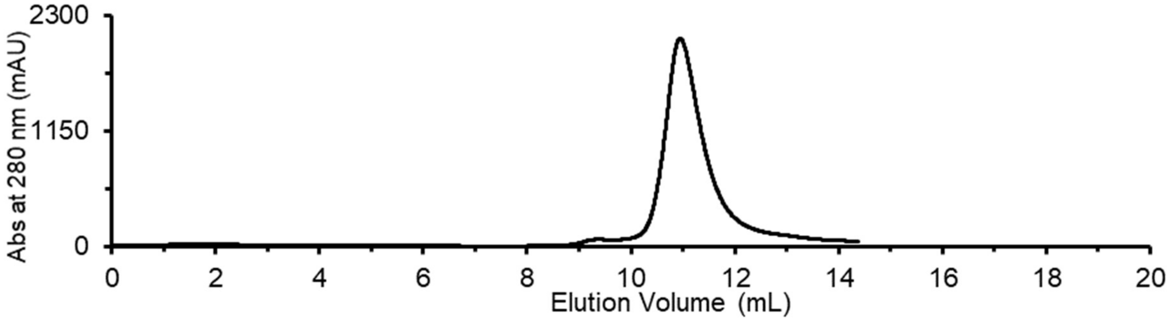

Starting Weight	Starting mol	Isolated Volume	Isolated [Protein] (mg/mL)	Isolated [Protein] (μM)	Isolated mol	Extrapolated Yield
2.6 mg	172.9 nmol	650 μL	1.0 mg / mL	72.1 μM	46.9 nmol	109.5 nmol
100% of Isolated Peptide	27.1% Folding Yield					

2.29. NEMO_iZIP Folding Results

Synthetic NEMO_iZIP was folded according to the protocol described in the Methods section, and the resulting folded protein concentration measured with ***Method 1.6.2***. Because of NEMO_iZIP's low extinction coefficient at 280 nm, the SEC chromatogram was monitored at 230 nm.

2.29.1. SEC Folding of L-NEMO_iZIP

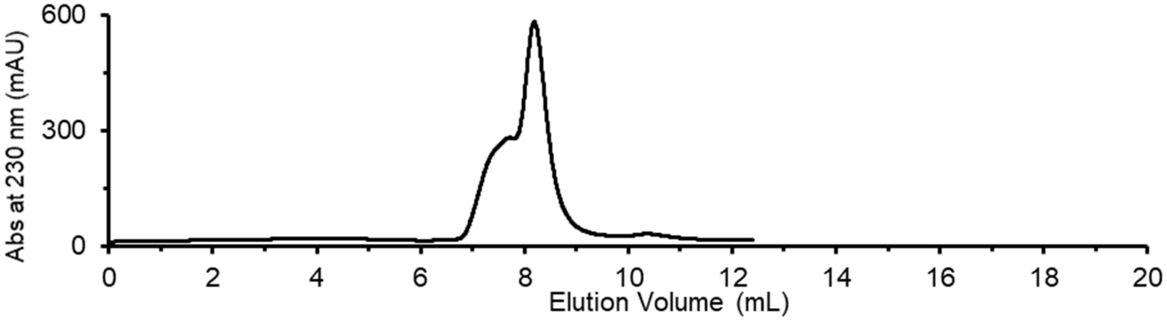

Starting Weight	Starting mol	Isolated Volume	Isolated [Protein] (mg/mL)	Isolated [Protein] (μM)	Isolated mol	Extrapolated Yield
0.9 mg	49.1 nmol	468 μL	0.2 mg / mL	15.7 μM	7.3 nmol	14.7 nmol
100% of Isolated Peptide	14.9% Folding Yield					

As there was a larger molecular weight shoulder that was removed upon the SEC purification, additional analytical SEC of the purified SEC fraction was recorded to verify its removal. The SEC purification performed as described in the Methods section, with minor modifications: the injected sample was 1 nmol of folded NEMO_iZIP not denatured, the chromatogram was recorded at 214 nm for increased sensitivity, and the running buffer was 50 mM TRIS, 150 mM NaCl, 5% Glycerol (v/v), pH 7.5.

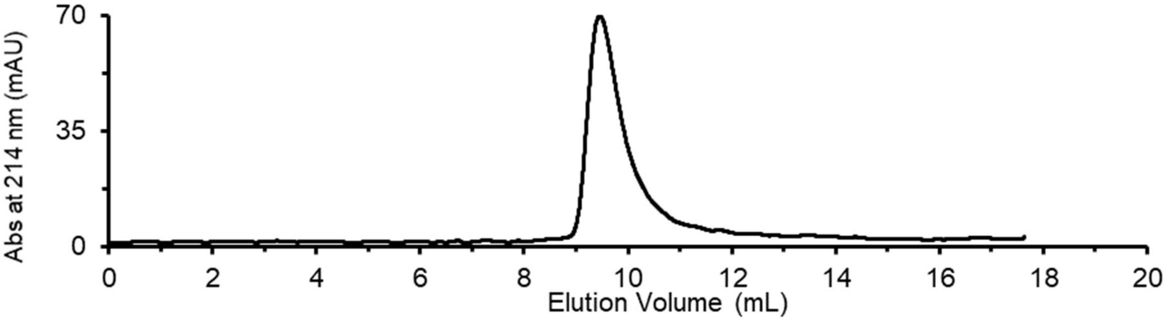

2.29.2. SEC Folding of D-NEMO_iZIP

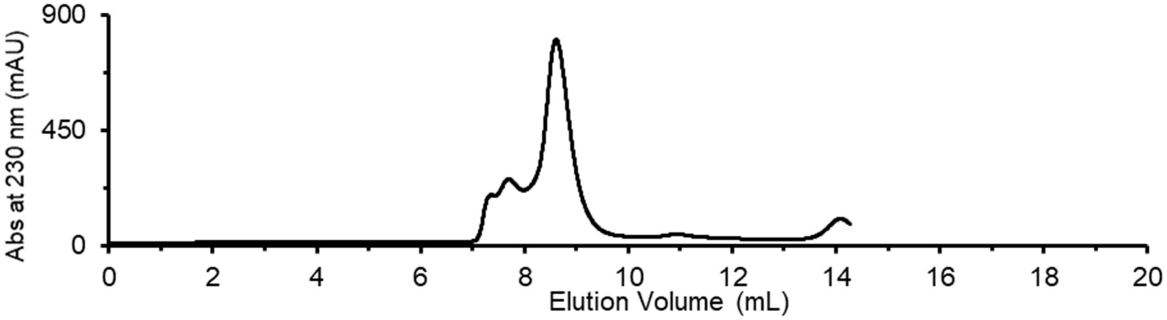

Starting Weight	Starting mol	Isolated Volume	Isolated [Protein] (mg/mL)	Isolated [Protein] (μM)	Isolated mol	Extrapolated Yield
2.0 mg	109.1 nmol	688 μL	0.2 mg / mL	15.5 μM	10.6 nmol	21.3 nmol
100% of Isolated Peptide	9.8% Folding Yield					

As there was a larger molecular weight shoulder that was removed upon the SEC purification, additional analytical SEC of the purified SEC fraction was recorded to verify its removal. The SEC purification performed as described in the Methods section, with minor modifications: the injected sample was 1 nmol of folded NEMO_iZIP not denatured, the chromatogram was recorded at 214 nm for increased sensitivity, and the running buffer was 50 mM TRIS, 150 mM NaCl, 5% Glycerol (v/v), pH 7.5.

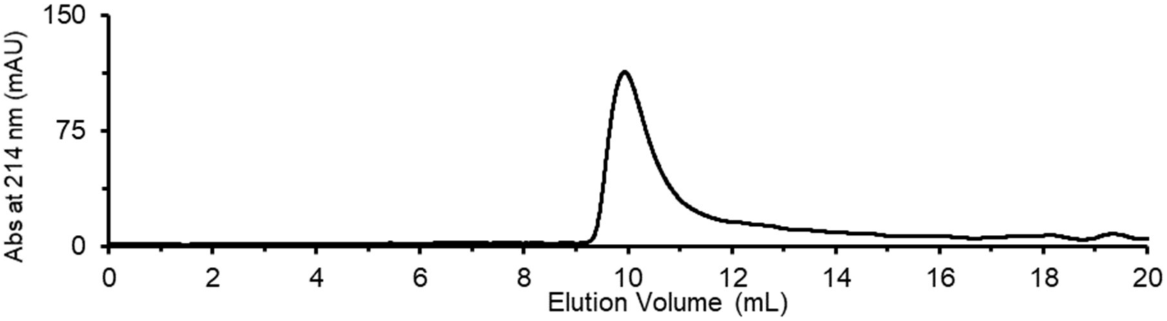

2.30. Max-Max Folding Results

Synthetic Max-Max was folded according to the protocol described in the Methods section, and the resulting folded protein concentration measured with ***Method 1.6.1***.

2.30.1. SEC Folding of L-Max-Max

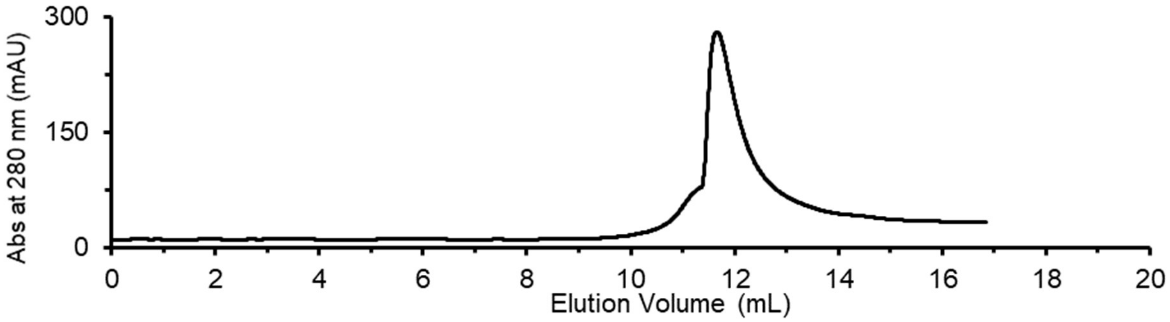

Starting Weight	Starting mol	Isolated Volume	Isolated [Protein] (mg/mL)	Isolated [Protein] (μM)	Isolated mol	Extrapolated Yield
1.2 mg	46.8 nmol	1074 μL	0.15 mg / mL	7.4 μM	7.9 nmol	
100% of Isolated Peptide	16.9% Folding Yield					

2.30.2. SEC Folding of D-Max-Max

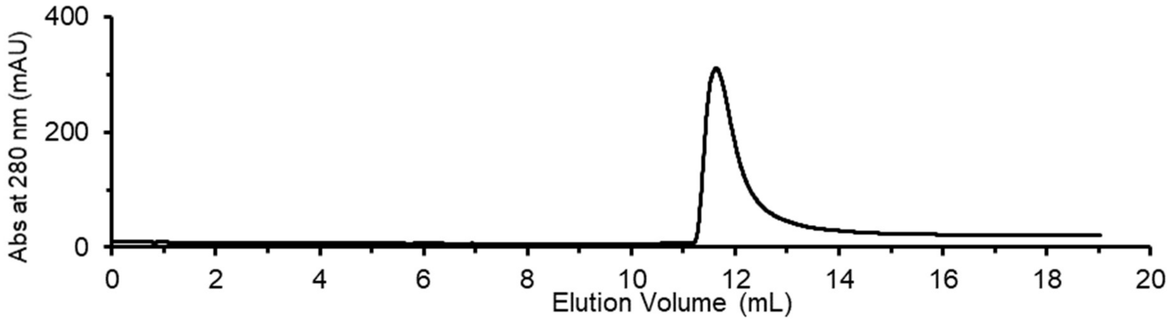

Starting Weight	Starting mol	Isolated Volume	Isolated [Protein] (mg/mL)	Isolated [Protein] (μM)	Isolated mol	Extrapolated Yield
0.7 mg	27.3 nmol	1237 μL	0.16 mg / mL	7.8 μM	9.6 nmol	
100% of Isolated Peptide	35.3% Folding Yield					

2.31. Myc-Max Folding Results

Synthetic Max-Max was folded according to the Methods section, and the resulting folded protein concentration measured with ***Method 1.6.1***.

2.31.1. SEC Folding of L-Myc-Max

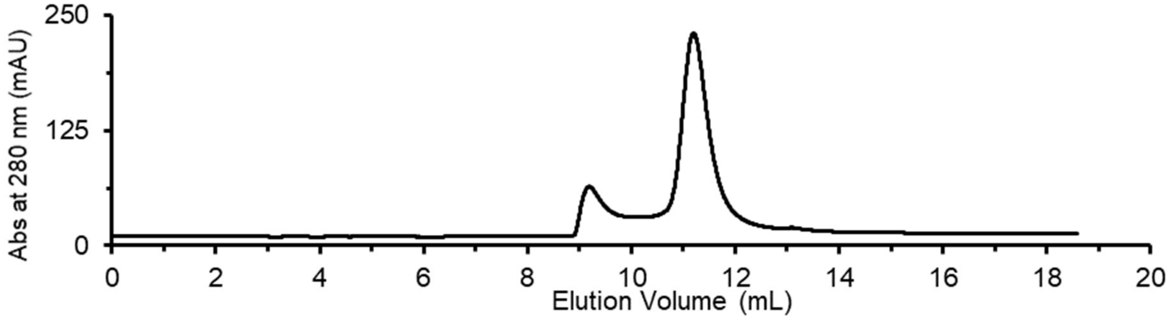

Starting Weight	Starting mol	Isolated Volume	Isolated [Protein] (mg/mL)	Isolated [Protein] (μM)	Isolated mol	Extrapolated Yield
0.6 mg	23.2 nmol	866 μL	0.3 mg / mL	14.5 μM	12.6 nmol	
100% of Isolated Peptide	54.4% Folding Yield					

2.31.2. SEC Folding of D-Myc-Max

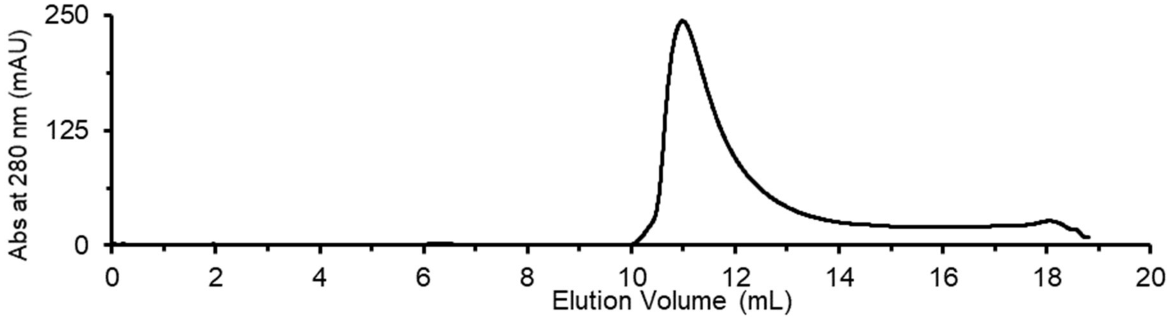

Starting Weight	Starting mol	Isolated Volume	Isolated [Protein] (mg/mL)	Isolated [Protein] (μM)	Isolated mol	Extrapolated Yield
1.3 mg	50.2 nmol	1128 μL	0.38 mg / mL	18.0 μM	20.4 nmol	
100% of Isolated Peptide	40.6% Folding Yield					

3. Supplementary Notes

3.1. Crystal Structure Collection Methods

To obtain the structures of the protein-peptide complexes, briefly, 10 mM peptides stock in 90% DMSO were added to the protein stocks to a final 1:1.25 protein:peptide molar ratio and screened against commercially available crystallization screens. Crystals were obtained by sitting hanging drop vapor diffusion methods at room temperature, with their crystallization conditions detailed in the Data Collection and Refinement Statistics tables below. Crystals were cryo-protected with glycerol or ethylene glycol followed by flash-freezing in liquid nitrogen. Diffraction datasets were collected at 100 K at a variety of sources as described in the tables. Data was processed in XDS,[68] autoPROC,[69] and AIMLESS.[70] Molecular replacement solutions were obtained using PHASER[71] with previously deposited high resolution PDB structures as search models. Complete models were built through iterative cycles of manual model building in COOT[72] and structure refinement using REFMAC[73] and PHENIX.[74] All the structure model figures in the paper were prepared using PyMOL (The PyMOL Molecular Graphics System, Version 2.4, Schrödinger, LLC), and ChimeraX.[75] The atomic coordinates and structure factors have been deposited in the Protein Data Bank, www.pdb.org.

3.2. MDM2 Cocrystal Structures

3.2.1. MDM2-H101 Data Collection and Refinement Statistics

**Title**	**MDM2_D-H101**
**PDB Entry**	**8F0Z**
Description	Structure of the MDM2 P53 binding domain in complex with D-H101, an all-D Helicon Polypeptide
Wavelength (Å)	1.03319
Resolution range (Å)	40.87-1.61 (1.64-1.61)
Space group	P 21 21 2
Unit cell: a,b,c (Å), α, β, γ (°)	40.87 81.36 34.98 90 90 90
Total reflections	200195(10013)
Unique reflections	15696(774)
Multiplicity	12.8(12.9)
Completeness (%)	99.6(100.0)
Mean I/sigma(I)	19.3(2.1)
Wilson B-factor (Å^2)	27.45
R-merge	0.067(1.18)
R-meas	0.072(1.28)
R-pim	0.028(0.48)
CC1/2	0.998(0.774)
Reflections used in refinement	15649 (1542)
Reflections used for R-free	756 (74)
R-work	0.2209 (0.3047)
R-free	0.2423 (0.3188)
Number of non-hydrogen atoms	1006
macromolecules	923
ligands	16
solvent	67
Protein residues	104
RMS(bonds) (Å)	0.009
RMS(angles) (°)	1.33
Ramachandran favored (%)	100
Ramachandran allowed (%)	0
Ramachandran outliers (%)	0
Rotamer outliers (%)	4.40
Clashscore	7.97
Average B-factor (Å^2)	35.27
macromolecules	34.61
ligands	31.76
solvent	45.08

Title	MDM2_D-H101
**PDB Entry**	**8F0Z**
Crystallization conditions	Crystals for MDM2 (6 mg/mL) were grown using 3.5 M Sodium Formate pH 7.0 as well solution, and cryopreserved with 3.5 M Sodium Formate pH 7.0, 30% Glycerol.
Beamline and Acknowledgements	APS, 23-ID-B: GM/CA@APS has been funded by the National Cancer Institute (ACB-12002) and the National Institute of General Medical Sciences (AGM-12006, P30GM138396). This research used resources of the Advanced Photon Source, a U.S. Department of Energy (DOE) Office of Science User Facility operated for the DOE Office of Science by Argonne National Laboratory under Contract No. DE-AC02-06CH11357.

3.2.2. MDM2-D-H102 Data Collection and Refinement Statistics

**Title**	**MDM2_D-H102**
**PDB Entry**	**8F10**
Description	Structure of the MDM2 P53 binding domain in complex with D-H102, an all-D Helicon Polypeptide
Wavelength (Å)	0.97933
Resolution range (Å)	42.17-1.28 (1.30-1.28)
Space group	P 1 21 1
Unit cell: a,b,c (Å), α, β, γ (°)	28.15 39.92 42.36 90 95.44 90
Total reflections	99690(439)
Unique reflections	20614(333)
Multiplicity	4.8(1.3)
Completeness (%)	85.3(29.1)
Mean I/sigma(I)	18.2(2.1)
Wilson B-factor (Å^2)	10.14
R-merge	0.039(0.30)
R-meas	0.049(0.42)
R-pim	0.029(0.30)
CC1/2	0.999(0.909)
Reflections used in refinement	20091 (736)
Reflections used for R-free	968 (38)
R-work	0.1587 (0.2479)
R-free	0.1708 (0.2064)
Number of non-hydrogen atoms	1062
macromolecules	908
ligands	49
solvent	105
Protein residues	102
RMS(bonds) (Å)	0.013
RMS(angles) (°)	1.33
Ramachandran favored (%)	97.65
Ramachandran allowed (%)	2.35
Ramachandran outliers (%)	0
Rotamer outliers (%)	0
Clashscore	10.35
Average B-factor (Å^2)	17.25
macromolecules	15.16
ligands	26.08
solvent	31.17

**Title**	**MDM2_D-H102**
**PDB Entry**	**8F10**
Crystallization conditions	Crystals for MDM2 (6 mg/mL) were grown using 0.01 M tri-Sodium citrate, 33% w/v PEG 6000 as well solution, and cryopreserved with 0.01 M tri-Sodium citrate, 33% w/v PEG 6000, 20% Glycerol.
Beamline and Acknowledgements	NSLS II, 17-ID-2 FMX: The FMX beamline is part of the Center for BioMolecular Structure (CBMS) which is primarily supported by the NIH, NIGMS through a Center Core P30 grant (grant no. P30GM133893), and by the US Department of Energy (DOE), Office of Biological and Environmental Research (contract no. KP1607011). NSLS-II is supported in part by the US DOE, Office of Science, Office of Basic Energy Sciences Program (contract nos. DE-SC0012704; KC0401040).

3.2.3. MDM2-D-H103 Data Collection and Refinement Statistics

**Title**	**MDM2_D-H103**
**PDB Entry**	**8F12**
Description	Structure of the MDM2 P53 binding domain in complex with D-H103, an all-D Helicon Polypeptide
Wavelength (Å)	1.03317
Resolution range (Å)	49.11-1.86(1.90-1.86)
Space group	P 43 21 2
Unit cell: a,b,c (Å), α, β, γ (°)	60.31 60.31 84.59 90 90 90
Total reflections	354507(19344)
Unique reflections	13715(817)
Multiplicity	25.8(23.7)
Completeness (%)	99.8(98.2)
Mean I/sigma(I)	18.0(2.2)
Wilson B-factor ($Å^2$)	25.46
R-merge	0.113(1.58)
R-meas	0.117(1.64)
R-pim	0.031(0.46)
CC1/2	0.999(0.851)
Reflections used in refinement	13668 (1318)
Reflections used for R-free	661 (54)
R-work	0.2172 (0.2821)
R-free	0.2422 (0.3280)
Number of non-hydrogen atoms	1073
macromolecules	971
ligands	25
solvent	77
Protein residues	111
RMS(bonds) (Å)	0.014
RMS(angles) (°)	1.38
Ramachandran favored (%)	96.77
Ramachandran allowed (%)	3.23
Ramachandran outliers (%)	0
Rotamer outliers (%)	1.03
Clashscore	4.01
Average B-factor ($Å^2$)	37.11
macromolecules	35.83
ligands	54.29
solvent	47.68

**Title**	**MDM2_D-H103**
**PDB Entry**	**8F12**
Crystallization conditions	Crystals for MDM2 (6 mg/mL) were grown using 3.2 M Ammonium sulfate, 0.1 M Citrate pH 5.0 as well solution, and cryopreserved with 3.2 M Ammonium sulfate, 0.1 M Citrate pH 5.0, 20% Glycerol.
Beamline and Acknowledgements	APS, 23-ID-B: GM/CA@APS has been funded by the National Cancer Institute (ACB-12002) and the National Institute of General Medical Sciences (AGM-12006, P30GM138396). This research used resources of the Advanced Photon Source, a U.S. Department of Energy (DOE) Office of Science User Facility operated for the DOE Office of Science by Argonne National Laboratory under Contract No. DE-AC02-06CH11357.

3.2.4. MDM2-D-H103-Alt Data Collection and Refinement Statistics

**Title**	**MDM2_D-H103-Alt**
**PDB Entry**	**8F13**
Description	Structure of the MDM2 P53 binding domain in complex with D-H103, an all-D Helicon Polypeptide, alternative C-terminus
Wavelength (Å)	1.03319
Resolution range (Å)	42.45-1.40(1.42-1.40)
Space group	P 43 21 2
Unit cell: a,b,c (Å), α, β, γ (°)	60.29 60.29 84.90 90 90 90
Total reflections	645852(13067)
Unique reflections	31020(1334)
Multiplicity	20.8(9.8)
Completeness (%)	98.4(86.0)
Mean I/sigma(I)	23.0(2.3)
Wilson B-factor (Å^2)	19.45
R-merge	0.083(1.27)
R-meas	0.087(1.42)
R-pim	0.025(0.61)
CC1/2	0.996(0.771)
Reflections used in refinement	30774 (2630)
Reflections used for R-free	1526 (121)
R-work	0.1744 (0.3414)
R-free	0.2078 (0.3765)
Number of non-hydrogen atoms	1175
macromolecules	974
ligands	51
solvent	150
Protein residues	112
RMS(bonds) (Å)	0.009
RMS(angles) (°)	1.44
Ramachandran favored (%)	97.85
Ramachandran allowed (%)	2.15
Ramachandran outliers (%)	0
Rotamer outliers (%)	6.25
Clashscore	7.27
Average B-factor (Å^2)	29.64
macromolecules	26.74
ligands	37.87
solvent	45.69

**Title**	**MDM2_D-H103-Alt**
**PDB Entry**	**8F13**
Crystallization conditions	Crystals for MDM2 (6 mg/mL) were grown using 0.2 M Potassium sodium tartrate, 0.1 M tri-Sodium citrate pH 5.6, 2.0 M Ammonium sulfate as well solution, and cryopreserved with 0.2 M Potassium sodium tartrate, 0.1 M tri-Sodium citrate pH 5.6, 2.0 M Ammonium sulfate, 30% EG.
Beamline and Acknowledgements	APS, 23-ID-B: GM/CA@APS has been funded by the National Cancer Institute (ACB-12002) and the National Institute of General Medical Sciences (AGM-12006, P30GM138396). This research used resources of the Advanced Photon Source, a U.S. Department of Energy (DOE) Office of Science User Facility operated for the DOE Office of Science by Argonne National Laboratory under Contract No. DE-AC02-06CH11357.

3.3.CHIP Co-Crystal Structures

3.3.1. CHIP-D-H201 Data Collection and Refinement Statistics

**Title**	**CHIP06_D-H201**
**PDB Entry**	**8F14**
Description	Structure of the STUB1 TPR domain in complex with D-H201, an all-D Helicon Polypeptide
Wavelength (Å)	0.89685
Resolution range (Å)	92.33-1.69(1.72-1.69)
Space group	C 2 2 21
Unit cell: a,b,c (Å), α, β, γ (°)	46.16 90.12 92.33 90 90 90
Total reflections	296534(14797)
Unique reflections	21997(1103)
Multiplicity	13.5(13.4)
Completeness (%)	100.0(100.0)
Mean I/sigma(I)	20.2(2.2)
Wilson B-factor ($Å^2$)	23.75
R-merge	0.069(1.22)
R-meas	0.075(1.32)
R-pim	0.028(0.50)
CC1/2	0.999(0.850)
Reflections used in refinement	21945 (2153)
Reflections used for R-free	1106 (116)
R-work	0.1967 (0.2927)
R-free	0.2351 (0.3368)
Number of non-hydrogen atoms	1422
macromolecules	1256
ligands	32
solvent	134
Protein residues	149
RMS(bonds) (Å)	0.011
RMS(angles) (°)	1.20
Ramachandran favored (%)	98.46
Ramachandran allowed (%)	1.54
Ramachandran outliers (%)	0
Rotamer outliers (%)	2.63
Clashscore	2.78
Average B-factor ($Å^2$)	34.98
macromolecules	34.11
ligands	39.38
solvent	42.10

**Title**	**CHIP06_D-H201**
**PDB Entry**	**8F14**
Crystallization conditions	Crystals for CHIP.06 (20 mg/mL) were grown using 0.2 M Lithium Sulfate, 0.1 M Bis-Tris pH 6.5, 25% w/v PEG 3350 as well solution, and cryopreserved with 0.2 M Lithium Sulfate, 0.1 M Bis-Tris pH 6.5, 25% w/v PEG 3350, 20% EG.
Beamline and Acknowledgements	Diamond I03: Diamond Light Source for beamtime, and the staff of beamlines I03 for assistance with crystal testing and data collection.

3.3.2. CHIP-D-H202 Data Collection and Refinement Statistics

**Title**	**CHIP06_D-H202**
**PDB Entry**	**8F15**
Description	Structure of the STUB1 TPR domain in complex with D-H202, an all-D Helicon Polypeptide
Wavelength (Å)	1.00000
Resolution range (Å)	49.02-1.73(1.76-1.73)
Space group	P 43
Unit cell: a,b,c (Å), α, β, γ (°)	50.56 50.56 199.85 90 90 90
Total reflections	721070(40585)
Unique reflections	51998(2848)
Multiplicity	13.9(14.3)
Completeness (%)	100.0(100.0)
Mean I/sigma(I)	16.6(2.2)
Wilson B-factor (Å^2)	17.46
R-merge	0.102(1.24)
R-meas	0.110(1.33)
R-pim	0.042(0.50)
CC1/2	0.999(0.785)
Reflections used in refinement	51994 (5201)
Reflections used for R-free	2477 (287)
R-work	0.1800 (0.2395)
R-free	0.2096 (0.2729)
Number of non-hydrogen atoms	4113
macromolecules	3702
ligands	79
solvent	332
Protein residues	448
RMS(bonds) (Å)	0.014
RMS(angles) (°)	1.43
Ramachandran favored (%)	98.73
Ramachandran allowed (%)	1.27
Ramachandran outliers (%)	0
Rotamer outliers (%)	3.78
Clashscore	3.91
Average B-factor (Å^2)	27.58
macromolecules	26.55
ligands	30.81
solvent	38.32

**Title**	**CHIP06_D-H202**
**PDB Entry**	**8F15**
Crystallization conditions	Crystals for CHIP.06 (20 mg/mL) were grown using 0.1 M TRIS pH 8, 30% v/v PEG 400 as well solution and was cryopreserved with 0.1 M TRIS pH 8, 30% v/v PEG 400, 25% EG.
Beamline and Acknowledgements	Spring8 BL45XU: RIKEN Structural Biology beamline I BL45XU Spring-8 in Japan.

3.3.3. CHIP-D-H203 Data Collection and Refinement Statistics

**Title**	**CHIP06_D-H203**
**PDB Entry**	**8F16**
Description	Structure of the STUB1 TPR domain in complex with D-H203, an all-D Helicon Polypeptide
Wavelength (Å)	1.18057
Resolution range (Å)	45.41-1.56(1.59-1.56)
Space group	P 21 21 21
Unit cell: a,b,c (Å), α, β, γ (°)	50.56 66.88 103.28 90 90 90
Total reflections	315958(16165)
Unique reflections	50341(2511)
Multiplicity	6.3(6.4)
Completeness (%)	99.5(100.0)
Mean I/sigma(I)	22.4(2.2)
Wilson B-factor (Å^2)	22.78
R-merge	0.030(0.68)
R-meas	0.036(0.80)
R-pim	0.019(0.43)
CC1/2	1.000(0.893)
Reflections used in refinement	50091 (5005)
Reflections used for R-free	2436 (257)
R-work	0.1979 (0.2629)
R-free	0.2302 (0.3036)
Number of non-hydrogen atoms	2942
macromolecules	2556
ligands	60
solvent	326
Protein residues	301
RMS(bonds) (Å)	0.011
RMS(angles) (°)	1.25
Ramachandran favored (%)	99.23
Ramachandran allowed (%)	0.77
Ramachandran outliers (%)	0
Rotamer outliers (%)	3.39
Clashscore	5.83
Average B-factor (Å^2)	28.91
macromolecules	27.74
ligands	34.66
solvent	37.03

**Title**	**CHIP06_D-H203**
**PDB Entry**	**8F16**
Crystallization conditions	Crystals for CHIP.06 (20 mg/mL) were grown using 0.1 M BICINE pH 8.5, 15% w/v PEG 1,500 as well solution, and were cryopreserved with 0.1 M BICINE pH 8.5, 15% w/v PEG 1,500, 25% EG.
Beamline and Acknowledgements	CLS, 08B1: Part or all of the research described in this paper was performed using beamline CMCF-BM at the Canadian Light Source, a national research facility of the University of Saskatchewan, which is supported by the Canada Foundation for Innovation (CFI), the Natural Sciences and Engineering Research Council (NSERC), the National Research Council (NRC), the Canadian Institutes of Health Research (CIHR), the Government of Saskatchewan, and the University of Saskatchewan.

3.3.4. CHIP-D-H204 Data Collection and Refinement Statistics

**Title**	**CHIP06_D-H204**
**PDB Entry**	**8F17**
Description	Structure of the STUB1 TPR domain in complex with D-H204, an all-D Helicon Polypeptide
Wavelength (Å)	1.18057
Resolution range (Å)	46.01-2.21(2.28-2.21)
Space group	P 1 21 1
Unit cell: a,b,c (Å), α, β, γ (°)	38.03 72.71 59.79 90 96.31 90
Total reflections	53489(4781)
Unique reflections	16124(1388)
Multiplicity	3.3(3.4)
Completeness (%)	99.0(98.6)
Mean I/sigma(I)	16.9(2.3)
Wilson B-factor (Å^2)	38.8
R-merge	0.032(0.40)
R-meas	0.045(0.56)
R-pim	0.032(0.40)
CC1/2	0.999(0.883)
Reflections used in refinement	15189 (1493)
Reflections used for R-free	764 (84)
R-work	0.2166 (0.2997)
R-free	0.2523 (0.3046)
Number of non-hydrogen atoms	2426
macromolecules	2338
ligands	36
solvent	52
Protein residues	292
RMS(bonds) (Å)	0.003
RMS(angles) (°)	0.51
Ramachandran favored (%)	97.64
Ramachandran allowed (%)	2.36
Ramachandran outliers (%)	0
Rotamer outliers (%)	1.40
Clashscore	4.70
Average B-factor (Å^2)	50.27
macromolecules	50.33
ligands	50.91
solvent	47.19

Title	CHIP06_D-H204
**PDB Entry**	**8F17**
Crystallization conditions	Crystals for CHIP.06 (20 mg/mL) were grown using 0.15 M Sodium chloride, 0.1 M TRIS pH 8, 8% w/v PEG 6000 as well solution, and was cryopreserved with 0.15 M Sodium chloride, 0.1 M TRIS pH 8, 8% w/v PEG 6000, 25% EG.
Beamline and Acknowledgements	CLS, 08B1: Part or all of the research described in this paper was performed using beamline CMCF-BM at the Canadian Light Source, a national research facility of the University of Saskatchewan, which is supported by the Canada Foundation for Innovation (CFI), the Natural Sciences and Engineering Research Council (NSERC), the National Research Council (NRC), the Canadian Institutes of Health Research (CIHR), the Government of Saskatchewan, and the University of Saskatchewan.

4. Supplementary References

1. N. Hartrampf, A. Saebi, M. Poskus, Z. P. Gates, A. J. Callahan, A. E. Cowfer, S. Hanna, S. Antilla, C. K. Schissel, A. J. Quartararo, X. Ye, A. J. Mijalis, M. D. Simon, A. Loas, S. Liu, C. Jessen, T. E. Nielsen, B. L. Pentelute, Synthesis of proteins by automated flow chemistry. *Science* **368**, 980 (2020).
2. B. R. Kelemen, T. A. Klink, M. A. Behike, S. R. Eubanks, P. A. Leland, R. T. Raines, Hypersensitive substrate for ribonucleases. *Nucleic Acids Research*. **27**, 3696–3701 (1999).
3. J. Yin, P. D. Straight, S. M. McLoughlin, Z. Zhou, A. J. Lin, D. E. Golan, N. L. Kelleher, R. Kolter, C. T. Walsh, Genetically encoded short peptide tag for versatile protein labeling by Sfp phosphopantetheinyl transferase. *Proceedings of the National Academy of Sciences*. **102**, 15815–15820 (2005).
4. A. H. Barczewski, M. J. Ragusa, D. F. Mierke, M. Pellegrini, The IKK-binding domain of NEMO is an irregular coiled coil with a dynamic binding interface. *Scientific Reports*. **9**, 2950 (2019).
5. M. Jbara, S. Pomplun, C. K. Schissel, S. W. Hawken, A. Boija, I. Klein, J. Rodriguez, S. L. Buchwald, B. L. Pentelute, Engineering Bioactive Dimeric Transcription Factor Analogs via Palladium Rebound Reagents. *J. Am. Chem. Soc.* **143**, 11788–11798 (2021).
6. W. Kabsch, XDS. *Acta Crystallographica Section D*. **66**, 125–132 (2010).
7. M. D. Winn, C. C. Ballard, K. D. Cowtan, E. J. Dodson, P. Emsley, P. R. Evans, R. M. Keegan, E. B. Krissinel, A. G. W. Leslie, A. McCoy, S. J. McNicholas, G. N. Murshudov, N. S. Pannu, E. A. Potterton, H. R. Powell, R. J. Read, A. Vagin, K. S. Wilson, Overview of the ıt CCP4 suite and current developments. *Acta Crystallographica Section D*. **67**, 235–242 (2011).
8. P. R. Evans, G. N. Murshudov, How good are my data and what is the resolution? *Acta Crystallographica Section D*. **69**, 1204–1214 (2013).
9. A. J. McCoy, R. W. Grosse-Kunstleve, P. D. Adams, M. D. Winn, L. C. Storoni, R. J. Read, Phaser crystallographic software. *Journal of Applied Crystallography*. **40**, 658–674 (2007).
10. P. Emsley, B. Lohkamp, W. G. Scott, K. Cowtan, Features and development of Coot. *Acta Crystallographica Section D*. **66**, 486–501 (2010).
11. G. N. Murshudov, P. Skubák, A. A. Lebedev, N. S. Pannu, R. A. Steiner, R. A. Nicholls, M. D. Winn, F. Long, A. A. Vagin, REFMAC5 for the refinement of macromolecular crystal structures. *Acta Crystallographica Section D*. **67**, 355–367 (2011).
12. D. Liebschner, P. V. Afonine, M. L. Baker, G. Bunkóczi, V. B. Chen, T. I. Croll, B. Hintze, L.-W. Hung, S. Jain, A. J. McCoy, N. W. Moriarty, R. D. Oeffner, B. K. Poon, M. G. Prisant, R. J. Read, J. S. Richardson, D. C. Richardson, M. D. Sammito, O. V. Sobolev, D. H. Stockwell, T. C. Terwilliger, A. G. Urzhumtsev, L. L. Videau, C. J. Williams, P. D. Adams, Macromolecular structure determination using X-rays, neutrons and electrons: recent developments in Phenix. *Acta Crystallographica Section D*. **75**, 861–877 (2019).
13. T. D. Goddard, C. C. Huang, E. C. Meng, E. F. Pettersen, G. S. Couch, J. H. Morris, T. E. Ferrin, UCSF ChimeraX: Meeting modern challenges in visualization and analysis. *Protein Science*. **27**, 14–25 (2018).
